# *Cirbp* suppression compromises DHODH-mediated ferroptosis defense and attenuates hypothermic cardioprotection in an aged donor transplantation model

**DOI:** 10.1172/JCI175645

**Published:** 2024-03-12

**Authors:** Yifan Zhu, Chenyu Jiang, Jian He, Chen He, Xingliang Zhou, Xu Huang, Yi Shen, Liwei Wu, Yongnan Li, Bei Feng, Yi Yan, Jun Li, Hao Zhang, Yiwei Liu

**Affiliations:** 1 Heart Center and Shanghai Institute of Pediatric Congenital Heart Disease, Shanghai Children’s Medical Center, National Children’s Medical Center,; 2 Department of Cardiothoracic Surgery, Shanghai Children’s Medical Center, National Children’s Medical Center, and; 3 Shanghai Clinical Research Center for Rare Pediatric Diseases, Shanghai Children’s Medical Center, National Children’s Medical Center, Shanghai Jiaotong University School of Medicine, Shanghai, China.; 4 State Key Laboratory of Oncogenes and Related Genes, Center for Single-Cell Omics, School of Public Health, Shanghai Jiao Tong University School of Medicine, Shanghai, China.; 5 Department of Cardiology, The Guangxi Zhuang Autonomous Region Workers’ Hospital, Nanning, China.; 6 Department of Cardiovascular Surgery, The Second Affiliated Hospital of Lanzhou University, Lanzhou, China.; 7 Department of Cardiology, Shanghai General Hospital, Shanghai Jiaotong University School of Medicine, Shanghai, China.

**Keywords:** Transplantation, Cardiovascular disease

## Abstract

Hypothermia is commonly used to protect donor hearts during transplantation. However, patients transplanted with aged donor hearts still have severe myocardial injury and decreased survival rates, but the underlying mechanism remains unknown. Because aged hearts are not considered suitable for donation, the number of patients awaiting heart transplants is increasing. In this study, we examined whether hypothermic cardioprotection was attenuated in aged donor hearts during transplantation and evaluated potential therapeutic targets. Using a rat heart transplantation model, we found that hypothermic cardioprotection was impaired in aged donor hearts but preserved in young donor hearts. RNA-Seq showed that cold-inducible RNA-binding protein (*Cirbp*) expression was decreased in aged donor hearts, and these hearts showed severe ferroptosis after transplantation. The young donor hearts from *Cirbp*-KO rats exhibited attenuated hypothermic cardioprotection, but *Cirbp* overexpression in aged donor hearts ameliorated hypothermic cardioprotection. Cardiac proteomes revealed that dihydroorotate dehydrogenase (DHODH) expression was significantly decreased in *Cirbp*-KO donor hearts during transplantation. Consequently, DHODH-mediated ubiquinone reduction was compromised, thereby exacerbating cardiac lipid peroxidation and triggering ferroptosis after transplantation. A cardioplegic solution supplemented with CIRBP agonists improved hypothermic cardioprotection in aged donor hearts, indicating that this method has the potential to broaden the indications for using aged donor hearts in transplantation.

## Abstract 2

Aging-induced depression of CIRBP in donor hearts compromises DHODH-mediated ferroptosis defense and attenuates cardioprotective effects of hypothermia during heart transplantation.

## Graphical abstract

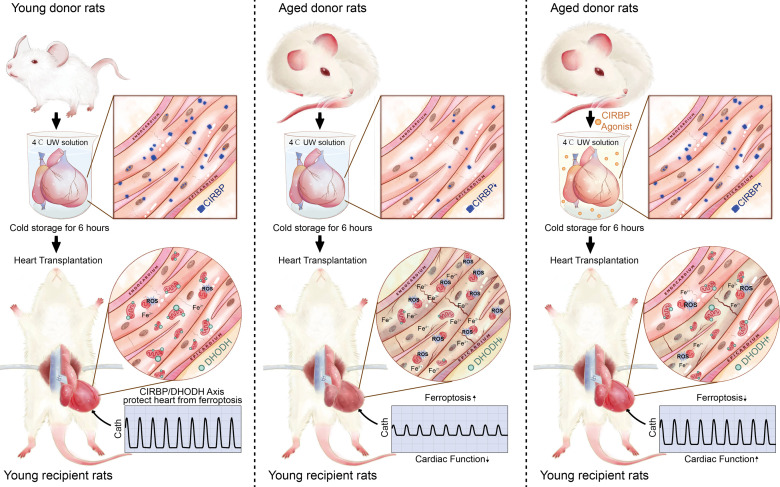

## Introduction

Currently, advanced heart failure places a constant and heavy burden on clinical and public health systems (1). Treatment options for heart failure include medication, ventricular assistive devices, and heart transplantation. However, for patients with end-stage heart failure, heart transplantation is the ultimate therapy. With the increasing incidence of end-stage heart failure, the number of patients awaiting heart transplantation has increased considerably (2). In the first few years of heart transplantation, the maximum donor age was set at 35 years (3, 4). However, strict adherence to conventional donor criteria has led to a severe scarcity of available donor hearts, resulting in protracted waiting times for desired organs and significantly increased mortality for those on waiting lists (2). As a result, approximately 50% of patients on waiting lists do not receive a heart transplant (5). Faced with such a dilemma, the International Society for Heart and Lung Transplantation has been challenged to find ways to broaden the indications for the use of marginal donor hearts that are considered unsuitable for transplantation, especially those from aged donors (6, 7). In recent decades, the global median donor age has progressively increased from 30 to 45 years in Europe (6), but aged hearts, which are from donors older than 45 years of age, are still rarely considered suitable because previous studies have shown that patients transplanted with aged donor hearts have an increased risk of primary allograft dysfunction and a decreased survival rate after transplantation (8, 9), but the underlying mechanism is still largely unknown.

We posited that the root of this mechanism might lie in a protein that responds to both aging and another form of physiological stress: hypothermia. During heart transplantation, the donor heart needs to be transported to the recipient patient within 6 hours, and ischemic injury to the donor heart is inevitable (10). The degree of ischemic injury to the donor heart is an important factor that determines the early survival rate of recipient patients after heart transplantation (6). One common strategy to protect the donor heart from myocardial injury during transplantation is hypothermia. The general consensus is that a low temperature confers cardioprotection by reducing metabolic activity and oxygen demand. However, recent studies have revealed additional mechanisms of hypothermic cardioprotection; for example, cold shock proteins exert protective effects at low temperatures. As a member of the cold shock protein family, cold-inducible RNA-binding protein (CIRBP) has been reported to play an important role in hypothermic cardioprotection during cardiopulmonary bypass. It was shown that CIRBP could be activated by cold stress, translocate from the nucleus to the cytoplasm to regulate its target mRNAs, and protect cardiomyocytes from ischemic injury (11). Thus, CIRBP might also be an essential effector of hypothermic cardioprotection during heart transplantation. In addition to hypothermia, aging also regulates the expression of CIRBP. A previous study showed that cellular CIRBP expression decreased gradually with increasing age (12). Therefore, we hypothesized that aging-induced suppression of CIRBP attenuated hypothermic cardioprotection in aged donor hearts, leading to severe myocardial injury during heart transplantation.

In this study, we established a rat heart transplantation model in which hypothermia was used to protect the donor heart. The results showed that aging-induced suppression of *Cirbp* expression impaired the cardioprotective effect of hypothermia by attenuating dihydroorotate dehydrogenase–mediated (DHODH-mediated) ferroptosis defense, resulting in exacerbated ferroptosis in aged donor hearts after transplantation. Importantly, we found that supplementing the cardioplegic solution with a *Cirbp* agonist improved the cardioprotection of aged donor hearts and alleviated ferroptosis after transplantation. This approach may broaden the indications for the use of aged donor hearts for transplantation and potentially benefit the many patients on waiting lists.

## Results

### The hypothermic cardioprotection of aged donor hearts is attenuated during cold storage in transplantation.

**Figure 1:**
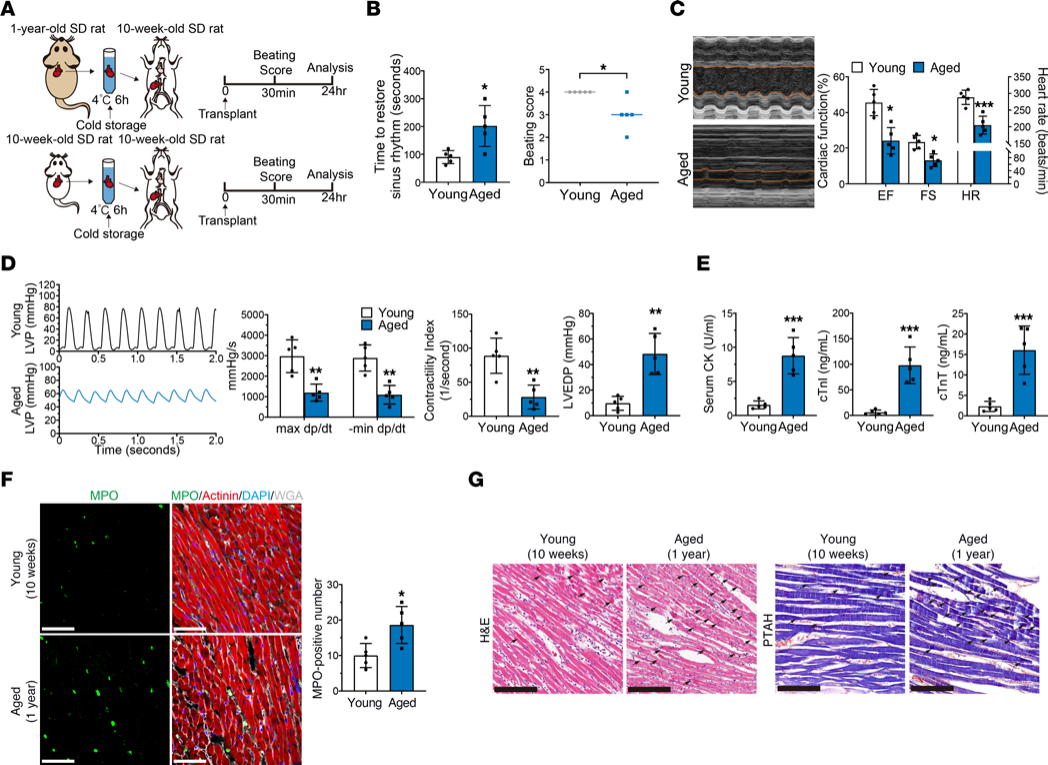
Hypothermic cardioprotection is attenuated in aged donor hearts during cold storage in transplantation. (**A**) Experimental design. Young and aged donor hearts were harvested and transplanted into young rats (*n* = 5 in each group). Hypothermia was used to protect donor hearts during cold storage. SD, Sprague-Dawley. (**B**) Assessment of cardiac resuscitation after transplantation. (**C**) Cardiac function of donor hearts measured by echocardiography after transplantation. (**D**) Representative left ventricular pressure (LVP) traces and cardiac function parameters of donor hearts determined via cardiac catheterization after transplantation. (**E**) Serum cardiac enzyme levels in recipients after transplantation. (**F**) MPO staining of donor hearts after transplantation. Scale bars: 50 μm. WGA, wheat germ agglutinin. (**G**) H&E and PTAH staining of donor hearts after transplantation. Arrows indicate myocardial contraction band necrosis. Scale bars: 100 μm (H&E); 50 μm (PTAH). Quantitative data are shown as the mean ± standard deviation with individual values presented as a dot plot. Statistical significance of the beating score was determined with the Mann-Whitney *U* test. Statistical significance of the other parameters was determined with the Student’s *t* test (*P < 0.05, **P < 0.01, and ***P < 0.001).

To simulate the process of heart transplantation, we established a heart transplantation rat model as previously described (13). Prior to transplantation, cardiac function was comparable between young (10-week-old) and aged (1-year-old) rats (Supplemental Figure 1; supplemental material available online with this article; https://doi.org/10.1172/JCI175645DS1). Donor hearts were harvested from the 2 groups, and arrest was induced with a cardioplegic solution (University of Wisconsin [UW] solution). Then, the donor hearts were immersed in UW solution, and hypothermia (4°C) was applied to protect the donor hearts. After 6 hours of cold storage, the donor hearts were transplanted into young rats (Figure 1A). Compared with those of young donor hearts, aged donor hearts required a significantly longer time to restore sinus rhythm and had a significantly decreased beating score after transplantation (Figure 1B), indicating that cardiac resuscitation was unsatisfactory in the aged donor hearts. On day 1 after transplantation, echocardiography showed that the aged donor hearts had lower left ventricular ejection fraction (EF), fractional shortening (FS), and heart rate (HR) values than did the young donor hearts (Figure 1C). Moreover, cardiac catheterization was performed and showed that aged donor hearts had reductions in maximum (max) dp/dt (rate of left ventricular pressure change with time), minimum (min) dp/dt, and the contractility index (CI) and increased left ventricular end-diastolic pressure (LVEDP) compared with young donor hearts (Figure 1D). Consistently, 7 days after transplantation, aged donor hearts also exhibited significantly decreased cardiac function compared with young donor hearts (Supplemental Figure 2). Moreover, 14 days after transplantation, the cardiac function of aged donor hearts was still lower than that of young donor hearts, although some cardiac functional parameters were not significantly different between the 2 groups (Supplemental Figure 3). These results suggested that aged donor hearts had more severe impairment of cardiac function after transplantation.

Furthermore, the levels of serum cardiac enzymes, including creatine kinase (CK), cardiac troponin I (cTnI), and cardiac troponin T (cTnT), were significantly higher in recipients that were transplanted with aged donor hearts than in those that were transplanted with young donor hearts (Figure 1E). In addition, myeloperoxidase (MPO) staining revealed more neutrophil infiltration in aged donor hearts than in young donor hearts (Figure 1F). Hematoxylin and eosin (H&E) and phosphotungstic acid–hematoxylin (PTAH) staining also revealed that aged donor hearts had more frequent sarcolemmal rupture, reduced clarity of nuclei in myocardial fibers, and prominent myocardial contraction band necrosis compared with young donor hearts (Figure 1G). These results indicated the occurrence of profound myocardial injury and a severe inflammatory response in aged donor hearts after transplantation.

Taken together, our findings suggested that the cardioprotective effects of hypothermia were attenuated in aged donor hearts during cold storage, leading to decreased cardiac function after transplantation.

### Ferroptosis is the major form of cell death in aged donor hearts after transplantation.

**Figure 2:**
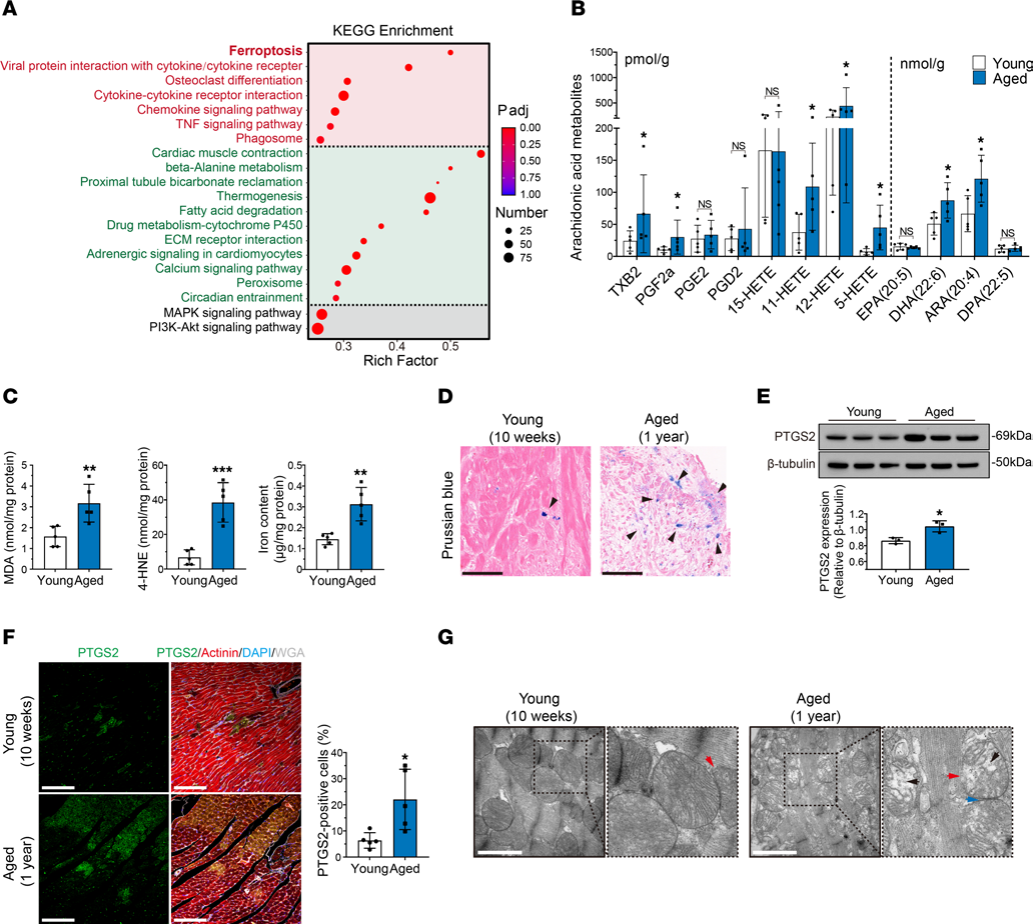
Ferroptosis is exacerbated in aged donor hearts after transplantation. (**A**) KEGG pathway enrichment analysis of upregulated (red) and downregulated (green) genes between the 2 groups. The young donor hearts were the denominators for comparison. The rich factor indicates the ratio of differentially expressed gene numbers annotated in this pathway term to all gene numbers annotated in this pathway term. *P* adj, adjusted *P* value. (**B**) Levels of arachidonic acid metabolites in donor hearts after transplantation. (**C**) Peroxidation parameters and iron overload in donor hearts after transplantation. (**D**) Prussian blue staining of donor hearts after transplantation. Arrowheads indicate iron deposition in myocardium. Scale bars: 50 μm. (**E**) Western blotting and quantification of PTGS2 in donor hearts after transplantation. (**F**) Immunofluorescence staining for PTGS2 in donor hearts after transplantation. Scale bars: 100 μm. (**G**) Transmission electron microscopy (TEM) images of donor hearts after transplantation. Arrowheads indicate mitochondrial shrinkage (blue), crista loss (black), and membrane rupture (red). Scale bars: 1 μm. Original magnification (enlarged insets), ×100,000. Quantitative data are shown as the mean ± standard deviation, with individual values presented as a dot plot. *P < 0.05, **P < 0.01, and ***P < 0.001, by 2-sided Student’s *t* test.

To clarify the major form of cell death in aged donor hearts after transplantation, we collected cardiac tissues from young and aged donor hearts on day 1 after transplantation and analyzed postoperative gene expression by RNA-Seq. A total of 3,415 genes showed significant differences between the 2 groups (fold change <0.67 or >1.5; *P* < 0.05). Principal component analysis (PCA) revealed obvious separation between the clusters in the 2 groups (Supplemental Figure 4). Interestingly, Kyoto Encyclopedia of Genes and Genomes (KEGG) pathway enrichment analysis of differentially expressed genes between the 2 groups revealed that the ferroptosis signaling pathway was significantly perturbed in aged donor hearts (Figure 2A).

Ferroptosis is the consequence of lipid peroxidation, which is triggered by the accumulation of polyunsaturated fatty acids; this process is characterized by dense and compact mitochondria with a loss of cristae (14). Thus, we performed ultraperformance liquid chromatography tandem mass spectrometry (UPLC-MS/MS) analysis to evaluate the arachidonic acid metabolite, a kind of polyunsaturated fatty acid, in young and aged donor hearts after transplantation. The results showed that the levels of arachidonic acid metabolites were higher in aged donor hearts than in young donor hearts, and for several of the metabolites, the differences were significant (Figure 2B). Furthermore, we analyzed the levels of lipid peroxidation and iron overload after transplantation and found that malondialdehyde (MDA), cardiac 4-hydroxy-2-nonenal (4-HNE), and iron levels were significantly increased in aged donor hearts compared with young donor hearts (Figure 2C). Prussian blue staining also showed that iron deposition was increased in aged donor hearts compared with young donor hearts (Figure 2D). Moreover, Western blot analysis and immunofluorescence staining revealed that the expression of cardiac prostaglandin endoperoxide synthase 2 (PTGS2), which is a putative molecular marker of ferroptosis (15), was significantly higher in aged donor hearts than in young donor hearts after transplantation (Figure 2, E and F). Finally, electron microscopy revealed that mitochondrial shrinkage was more apparent in aged donor hearts than in young donor hearts and was accompanied by exacerbated cristae loss and increased membrane rupture (Figure 2G). The Seahorse assay also revealed that mitochondrial function was significantly lower in aged donor hearts than in young donor hearts (Supplemental Figure 5). These results demonstrated that aged donor hearts exhibited severe ferroptosis after transplantation. Importantly, we further evaluated the levels of other forms of cell death, such as apoptosis and pyroptosis, in young and aged donor hearts after transplantation, and the results showed that these forms of cell death were comparable between the 2 groups (Supplemental Figure 6).

**Figure 3:**
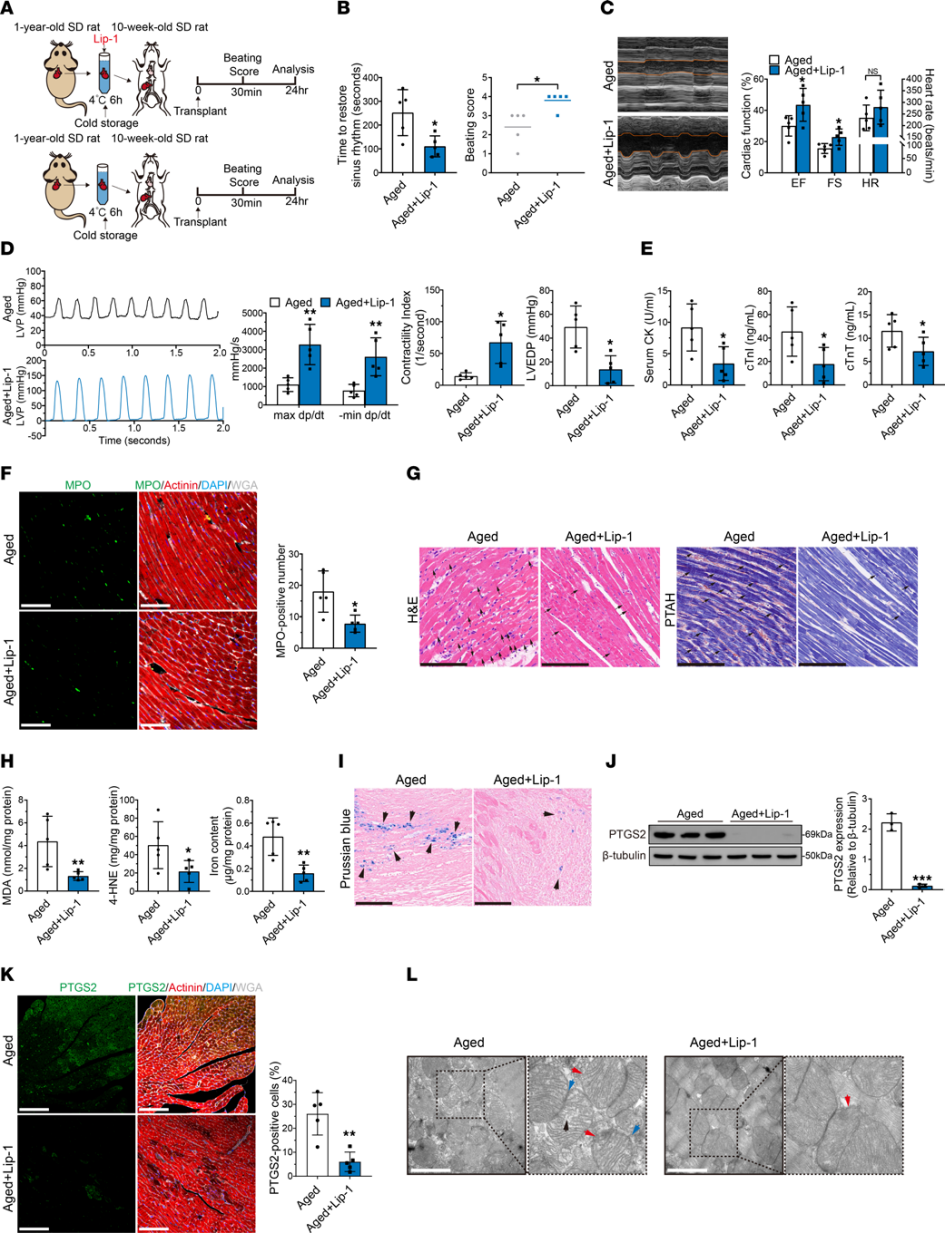
Cardioplegic solution supplemented with liproxstatin-1 could improve cardioprotection of aged donor heart during cold storage in transplantation. (**A**) Experimental design. Aged donor hearts were harvested and transplanted into young rats. UW solution was used to induce cardiac arrest in the control rats, and UW solution supplemented with liproxstatin-1 (Lip-1) was used in the experimental group (*n* = 5 in each group). Hypothermia was used to protect the donor heart during cold storage. (**B**) Assessment of cardiac resuscitation after transplantation. (**C**) Cardiac function of donor hearts measured by echocardiography after transplantation. (**D**) Representative LVP traces and cardiac function parameters of donor hearts determined via cardiac catheterization after transplantation. (**E**) Serum cardiac enzymes in recipients after transplantation. (**F**) MPO staining of donor hearts after transplantation. Scale bars: 50 μm. (**G**) H&E and PTAH staining of donor hearts after transplantation. Arrows indicate myocardial contraction band necrosis. Scale bars: 100 μm (H&E); 50 μm (PTAH). (**H**) Peroxidation parameters and iron overload in donor hearts after transplantation. (**I**) Prussian blue staining of donor hearts after transplantation. Arrowheads indicate iron deposition in myocardium. Scale bars: 50 μm. (**J**) Western blotting and quantification of PTGS2 in donor hearts after transplantation. (**K**) Immunofluorescence staining of PTGS2 in donor hearts after transplantation. Scale bars: 100 μm. (**L**) TEM images of donor hearts after transplantation. Arrowheads indicate mitochondrial shrinkage (blue), crista loss (black), and membrane rupture (red). Scale bars: 1 μm. Original magnification (enlarged insets), ×100,000. Quantitative data are shown as the mean ± standard deviation, with individual values presented as a dot plot. Statistical significance of the beating score was determined with the Mann-Whitney *U* test. *P < 0.05, **P < 0.01, and ***P < 0.001, by 2-sided Student’s *t* test.

To explore whether ferroptosis impairs cardiac function in aged donor hearts after transplantation, we added a ferroptosis inhibitor (liproxstatin-1, 10 μM) to the UW solution and evaluated its cardioprotective effects (Figure 3A). Compared with those in the control group, the addition of liproxstatin-1 significantly shortened the time needed to restore sinus rhythm and improved the beating score of aged donor hearts after transplantation (Figure 3B). Notably, echocardiography and cardiac catheterization showed that postoperative cardiac function was significantly improved in aged donor hearts, as indicated by the significant increases in EF, FS, max dp/dt, min dp/dt, and CI and a decrease in LVEDP (Figure 3, C and D). Moreover, the addition of liproxstatin-1 to the cardioplegic solution significantly alleviated the changes in serum cardiac enzymes in the recipients of aged donor hearts (Figure 3E). MPO, H&E, and PTAH staining revealed that neutrophil infiltration and myocardial contraction band necrosis were alleviated after the addition of liproxstatin-1 (Figure 3, F and G). Furthermore, the addition of liproxstatin-1 significantly decreased the levels of MDA, 4-HNE, and iron in aged donor hearts after transplantation (Figure 3H). Prussian blue staining also showed that iron deposition was decreased in aged donor hearts after liproxstatin-1 supplementation (Figure 3I). Finally, liproxstatin-1 significantly decreased the expression of PTGS2 in aged donor hearts (Figure 3, J and K), and electron microscopy showed that the ferroptosis-related mitochondrial changes in aged donor hearts were mitigated by the addition of liproxstatin-1 (Figure 3L). These results indicated that liproxstatin-1 inhibited ferroptosis in aged donor hearts, which significantly improved cardiac function after transplantation. Consistently, we added another ferroptosis inhibitor (ferrostatin-1, 10 μM) to the cardioplegic solution and found that postoperative cardiac function was significantly improved in aged donor hearts as well (Supplemental Figure 7).

In conclusion, these data suggest that ferroptosis was the major form of cell death in aged donor hearts after transplantation caused by the attenuation of hypothermic cardioprotection during cold storage.

### Decreased Cirbp expression underlies the attenuated hypothermic cardioprotection of aged donor hearts during cold storage in transplantation.

**Figure 4:**
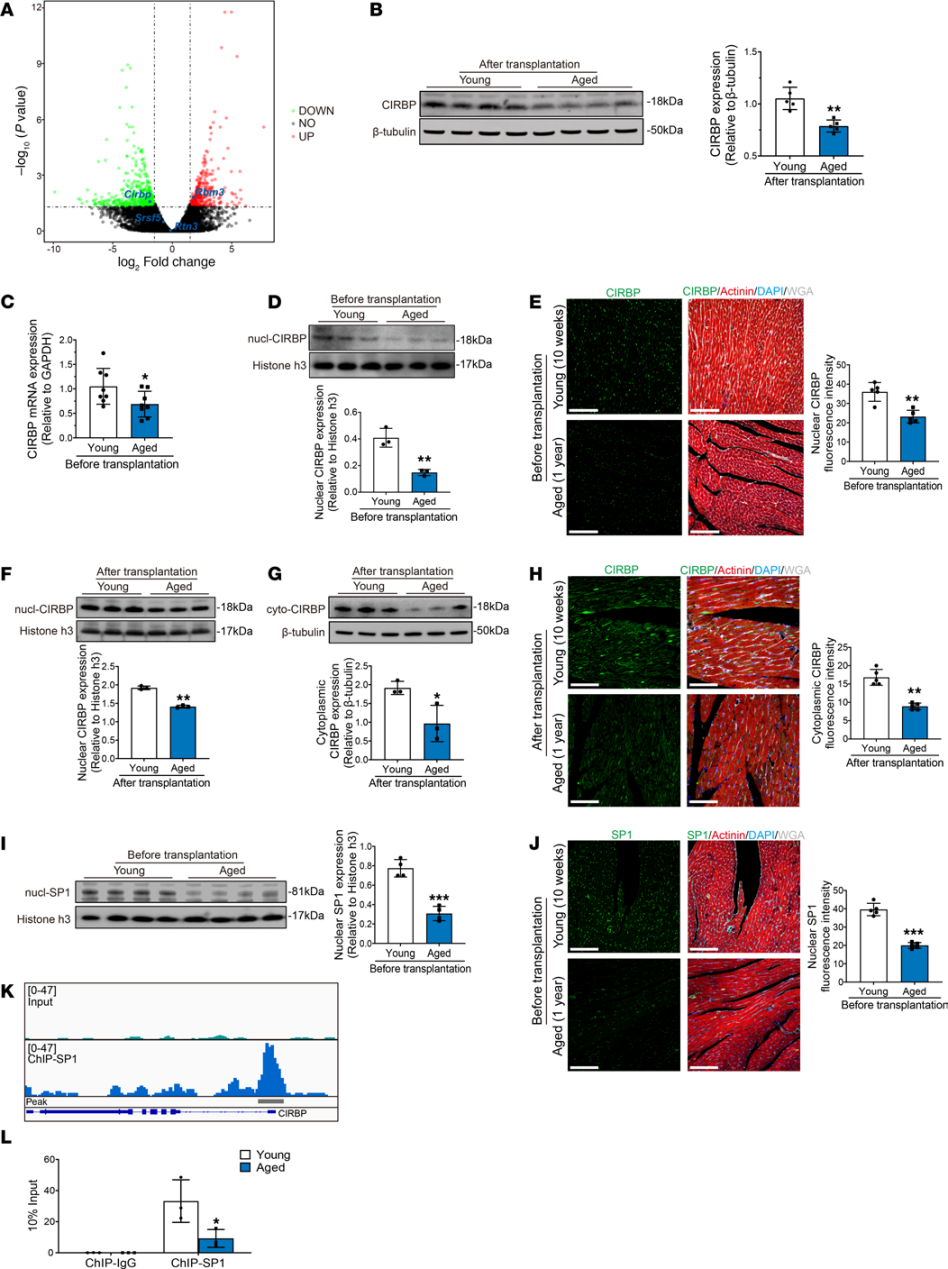
Decreased expression of SP1 suppresses CIRBP expression in aged donor heart. (**A**) Volcano plots showing the differentially expressed genes between young and aged donor hearts after transplantation. Green dots represent genes whose expression was significantly decreased (DOWN) in aged donor hearts. Red dots represent genes whose expression was significantly increased (UP) in aged donor hearts. Gray dots represent genes whose expression did not reach statistical significance (NO). (**B**) Western blotting and quantification of CIRBP in young and aged donor hearts after transplantation. (**C**) Real-time qPCR analysis of *Cirbp* mRNA expression in young and aged donor hearts before transplantation. (**D**) Western blotting and quantification of nuclear CIRBP in young and aged donor hearts before transplantation. (**E**) Immunofluorescence staining for CIRBP in young and aged donor hearts before transplantation and fluorescence intensity of nuclear CIRBP. Scale bars: 100 μm. (**F**) Western blotting and quantitation of nuclear CIRBP in young and aged donor hearts after transplantation. (**G**) Western blotting and quantitation of cytoplasmic CIRBP in young and aged donor hearts after transplantation. (**H**) Immunofluorescence staining for CIRBP in young and aged donor hearts after transplantation and fluorescence intensity of CIRBP. Scale bars: 50 μm. (**I**) Western blotting and quantitation of nuclear SP1 in young and aged donor hearts before transplantation. (**J**) Immunofluorescence staining for SP1 in young and aged donor hearts before transplantation and fluorescence intensity of SP1. Scale bars: 100 μm. (**K**) Integrative Genomics Viewer (IGV) screenshot of SP1 ChIP-Seq data for the *Cirbp* gene. (**L**) The degree to which SP1 binds the *Cirbp* promoter region was determined by ChIP-qPCR (*n* = 3 in each group). Quantitative data are shown as the mean ± standard deviation, with individual values presented as a dot plot. *P < 0.05, **P < 0.01, and ***P < 0.001, by 2-sided Student’s *t* test.

Previous studies have shown that cold shock proteins play a critical role in cellular protection under low temperature conditions (16). Because hypothermic cardioprotection was impaired in aged donor hearts, we hypothesized that this effect could be attributed to aging-induced suppression of a certain cold shock protein. Thus, we analyzed the expression levels of cold shock proteins by RNA-Seq. Among the major cold shock proteins, including CIRBP, RNA-binding motif protein 3 (RBM3), reticulon 3 (RTN3), and serine and arginine rich splicing factor 5 (SRSF5), only the expression of *Cirbp* was significantly downregulated in aged donor hearts compared with young donor hearts (Figure 4A). Western blot analysis further verified that postoperative CIRBP expression was significantly lower in aged donor hearts than in young donor hearts (Figure 4B). Therefore, we selected CIRBP as the target gene in subsequent studies.

It has been reported that CIRBP translocates from the nucleus to the cytoplasm in response to cold stress and subsequently exerts its biological effects (11, 17). Thus, we analyzed cardiac CIRBP expression at baseline (before transplantation) and its response to cold stress in young and aged donor hearts during transplantation. At baseline, real-time quantitative PCR (qPCR) revealed that cardiac *Cirbp* mRNA levels were significantly lower in aged donor hearts than in young donor hearts (Figure 4C). Furthermore, Western blotting and immunofluorescence staining revealed that the nuclear abundance of CIRBP was significantly lower in aged donor hearts at baseline than in young donor hearts (Figure 4, D and E). Notably, we found that aged human donor hearts also had significantly decreased nuclear abundance of CIRBP at baseline compared with young human donor hearts (Supplemental Figure 8, A and B and Supplemental Table 1). Next, we evaluated the abundance of nuclear and cytoplasmic CIRBP in young and aged donor hearts after transplantation. We found that both nuclear and cytoplasmic CIRBP levels were significantly lower in aged donor hearts than in young donor hearts after transplantation (Figure 4, F and G). Immunofluorescence staining also revealed that more CIRBP had translocated from the nucleus to the cytoplasm in young donor hearts than in aged donor hearts after transplantation (Figure 4H).

Specificity protein 1 (SP1) is a transcription factor that regulates the transcription of the *Cirbp* gene in the nucleus (18), and a study showed that cellular SP1 expression decreases with age (19). Therefore, we hypothesized that the decrease in *Cirbp* expression in aged donor hearts was due to the decrease in SP1 expression. To investigate this hypothesis, we measured cardiac SP1 expression at baseline in young and aged donor hearts by Western blotting and immunofluorescence staining and found significantly lower nuclear levels of SP1 in aged donor hearts than in young donor hearts (Figure 4, I and J). Furthermore, the nuclear abundance of SP1 was also lower in aged human donor hearts than in young human donor hearts (Supplemental Figure 8, C and D). Next, we performed a chromatin immunoprecipitation (ChIP) assay to evaluate the binding of SP1 to the *Cirbp* promoter region in aged donor hearts. The ChIP-Seq results showed considerable enrichment of SP1 at its binding site within the promoter region in the 5′-UTR of *Cirbp* (Figure 4K), and ChIP-qPCR showed that aging caused a significant decrease in SP1 binding to the *Cirbp* promoter region (Figure 4L). In addition, we used adeno-associated virus serotype 9 (AAV9) to induce overexpression of myocardial *Sp1* in aged rats (AAV9-*Sp1*) (Supplemental Figure 9A). Western blot analysis revealed that CIRBP expression was significantly increased in aged donor hearts harvested from rats treated with AAV9-*Sp1* compared with those harvested from vector-treated rats (AAV9-null) (Supplemental Figure 9B), indicating that the decrease in SP1 expression was responsible for CIRBP suppression in aged donor hearts. Moreover, *Sp1*-overexpressing aged donor hearts exhibited less myocardial ferroptosis and improved cardiac function after transplantation, suggesting that the SP1/CIRBP axis could mediate hypothermic cardioprotection during cold storage (Supplemental Figure 9, C–L).

Notably, we further evaluated the dynamic changes in the expression of SP1 and CIRBP in donor hearts from older rats, and the results showed that donor hearts from rats older than 1 year also had decreased SP1 and CIRBP levels (Supplemental Figure 10), indicating that *Cirbp* suppression progressed in the donor heart with increasing age.

Collectively, these results showed that aging-induced decreases in SP1 expression reduced its binding to the *Cirbp* promoter, leading to decreased *Cirbp* expression at baseline as well as decreased CIRBP translocation from the nucleus to the cytoplasm during cold storage in heart transplantation.

### The hypothermic cardioprotection of young Cirbp-KO donor hearts is attenuated during cold storage in transplantation.

**Figure 5:**
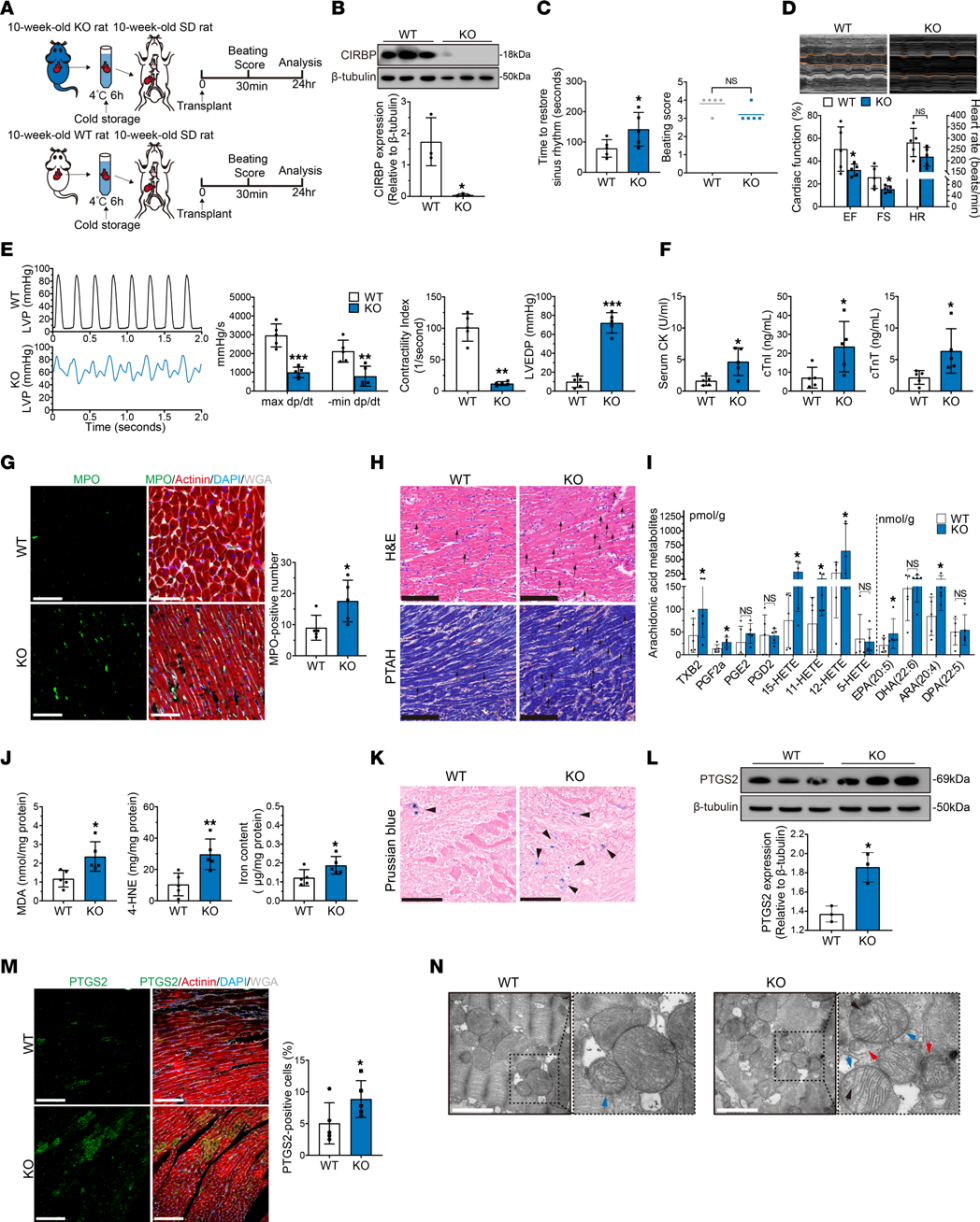
*Cirbp* deficiency impairs hypothermic cardioprotection in young donor hearts during cold storage in transplantation. (**A**) Experimental design. Young donor hearts were harvested from WT or *Cirbp*-KO rats and transplanted into young WT rats (*n* = 5 in each group). Hypothermia was used to protect donor hearts during cold storage. (**B**) Western blotting and quantification of CIRBP in donor hearts after transplantation. (**C**) Assessment of cardiac resuscitation after transplantation. (**D**) Cardiac function of donor hearts measured by echocardiography after transplantation. (**E**) Representative LVP traces and cardiac function parameters of donor hearts determined via cardiac catheterization after transplantation. (**F**) Serum cardiac enzymes in recipients after transplantation. (**G**) MPO staining of donor hearts after transplantation. Scale bars: 50 μm. (**H**) H&E and PTAH staining of donor hearts after transplantation. Arrows indicate myocardial contraction band necrosis. Scale bars: 100 μm (H&E); 50 μm (PTAH). (**I**) Levels of arachidonic acid metabolites in donor hearts after transplantation. (**J**) Peroxidation parameters and iron overload in donor hearts after transplantation. (**K**) Prussian blue staining of donor hearts after transplantation. Arrowheads indicate iron deposition in myocardium. Scale bars: 50 μm. (**L**) Western blotting and quantification of PTGS2 in donor hearts after transplantation. (**M**) Immunofluorescence staining of PTGS2 in donor hearts after transplantation. Scale bars: 100 μm. (**N**) TEM images of donor hearts after transplantation. Arrowheads indicate mitochondrial shrinkage (blue), crista loss (black), and membrane rupture (red). Scale bars: 1 μm. Data shown are the mean ± standard deviation, with individual values presented as a dot plot. Statistical significance of the beating score was determined with the Mann-Whitney *U* test. Statistical significance of the other parameters was determined by Student’s *t* test (*P < 0.05, **P < 0.01, and ***P < 0.001).

To further explore the role of CIRBP in hypothermic cardioprotection during cold storage, we generated *Cirbp*-KO rats and confirmed the KO of *Cirbp* (Supplemental Figure 11, A and B). At baseline, *Cirbp*-KO rats exhibited no developmental defects or inherent physiological abnormalities. Histological and echocardiographic analyses revealed no pathological changes or abnormalities in cardiac function in the *Cirbp*-KO rats (Supplemental Figure 11, C and D). Young donor hearts were subsequently harvested from WT (10-week-old) and *Cirbp*-KO (10-week-old) rats and transplanted into WT (10-week-old) rats after 6 hours of cold storage (Figure 5A). Western blot analysis revealed that CIRBP was not expressed in young *Cirbp*-KO donor hearts (Figure 5B). Compared with young WT donor hearts, young *Cirbp*-KO donor hearts took a longer time to regain sinus rhythm and had decreased beating scores after transplantation (Figure 5C), indicating that young *Cirbp*-KO donor hearts had a prolonged recovery time after transplantation. On day 1 after transplantation, echocardiography and cardiac catheterization showed that young *Cirbp*-KO donor hearts had significantly decreased EF, FS, max dp/dt, min dp/dt, and CI and significantly increased LVEDP compared with young WT donor hearts (Figure 5, D and E). Consistently, 7 days after transplantation, young *Cirbp*-KO donor hearts also exhibited significantly decreased cardiac function compared with young WT donor hearts (Supplemental Figure 12). These results suggested that young *Cirbp*-KO donor hearts exhibited more severe impairment of cardiac function after transplantation. Furthermore, compared with those in the control group, the recipient rats that were transplanted with young *Cirbp*-KO donor hearts showed significantly increased levels of serum cardiac enzymes (Figure 5F). In addition, immunofluorescence staining with MPO showed that young *Cirbp*-KO donor hearts had greater infiltration of neutrophils than did young WT donor hearts (Figure 5G). Moreover, H&E and PTAH staining revealed more myocardial contraction band necrosis in young *Cirbp*-KO donor hearts than in young WT donor hearts (Figure 5H). These results indicated the occurrence of profound myocardial injury and a severe inflammatory response in young *Cirbp*-KO donor hearts after transplantation.

Notably, after transplantation, arachidonic acid metabolite levels were significantly increased in young *Cirbp*-KO donor hearts compared with young WT donor hearts (Figure 5I). Moreover, MDA, 4-HNE and iron levels were significantly increased in young *Cirbp*-KO donor hearts (Figure 5J). In addition, Prussian blue staining revealed that iron deposition was increased in young *Cirbp*-KO donor hearts compared with WT donor hearts (Figure 5K). Furthermore, Western blot analysis and immunofluorescence staining revealed that myocardial expression of PTGS2 was significantly increased in young *Cirbp*-KO donor hearts compared with young WT donor hearts (Figure 5, L and M). Finally, electron microscopy revealed that mitochondrial shrinkage and crista loss were greater in young *Cirbp*-KO donor hearts than in young WT donor hearts (Figure 5N). These results indicated that ferroptosis was triggered in young *Cirbp*-KO donor hearts after transplantation.

Additionally, we used AAV9-*Sp1* to overexpress myocardial *Sp1* in aged *Cirbp*-KO donor hearts, but overexpression of *Sp1* failed to improve their cardiac functions after transplantation in the condition of *Cirbp* KO, suggesting that CIRBP is an essential downstream effector of SP1 in hypothermic cardioprotection (Supplemental Figure 13).

Taken together, these results suggested that hypothermic cardioprotection was attenuated in the young *Cirbp*-KO donor hearts, resulting in aggravated ferroptosis after transplantation.

### Cirbp KO exacerbates ferroptosis in cardiomyocytes after cold ischemia.

**Figure 6:**
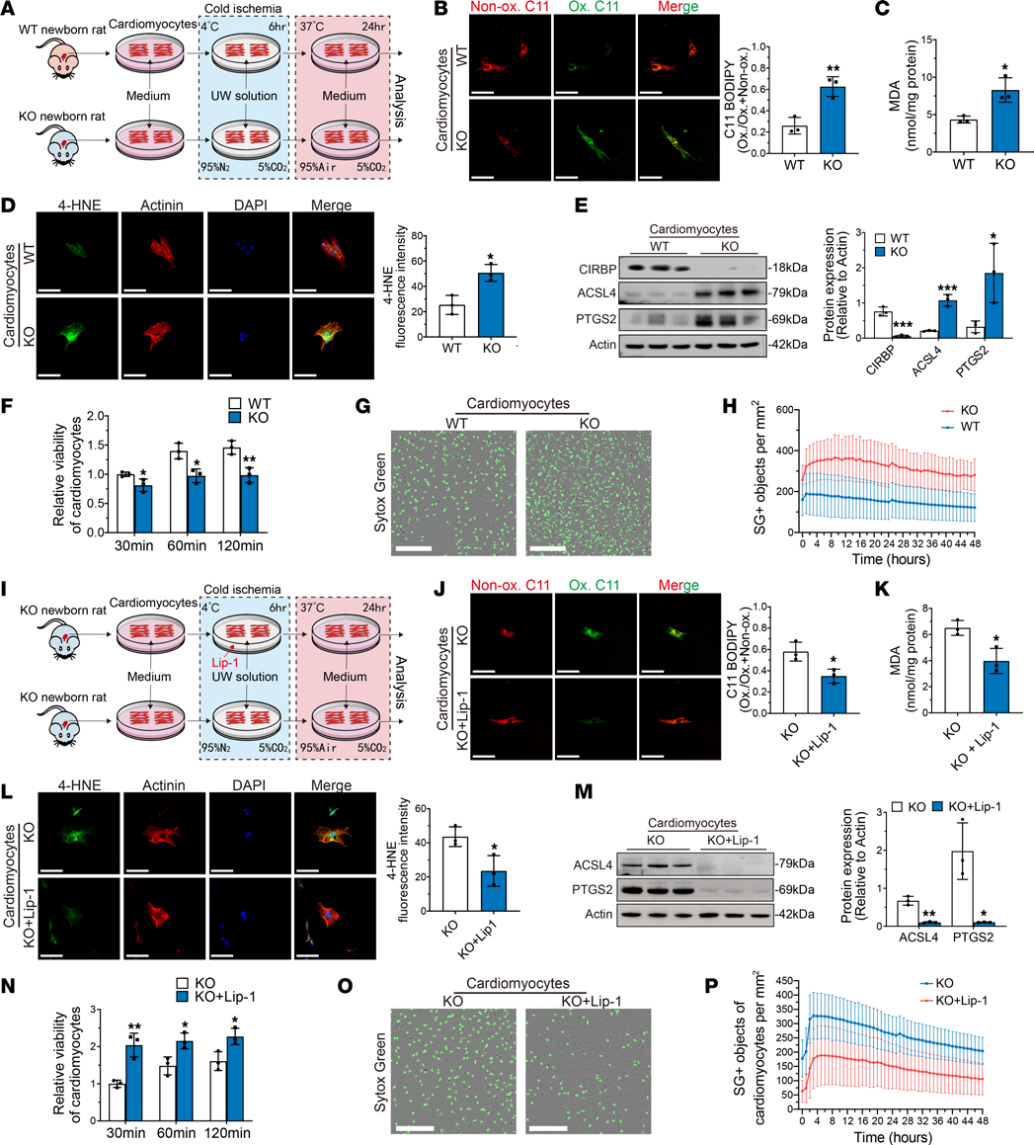
Ferroptosis is exacerbated in *Cirbp*-KO cardiomyocytes after cold ischemia. (**A**) Experimental design of the first part. The cardiomyocytes were isolated from WT or *Cirbp*-KO neonatal rats and subjected to cold ischemia. Both groups were treated with UW solution during cold ischemia. (**B**) Cardiomyocytes labeled with BODIPY 581/591 C11 after cold ischemia. The graph shows the ratio of oxidized–to–total BODIPY 581/591 C11. Ox., oxidized; Non-ox., nonoxidized. Scale bars: 25 μm. (**C**) MDA levels in cardiomyocytes after cold ischemia. (**D**) 4-HNE staining of cardiomyocytes after cold ischemia. Scale bars: 25 μm. (**E**) Western blotting and quantification of CIRBP, ACSL4, and PTGS2 in cardiomyocytes after cold ischemia. (**F**) Cell viability of cardiomyocytes after cold ischemia. (**G**) Live-cell imaging of cardiomyocytes incubated with SYTOX Green after cold ischemia. Scale bars: 300 μm. (**H**) Time-lapse analysis of cell death in cardiomyocytes after cold ischemia. (**I**) Experimental design of the second part. The cardiomyocytes were isolated from *Cirbp*-KO neonatal rats and subjected to cold ischemia. One group is treated with UW solution and the other group is treated with UW solution supplemented with liproxstatin-1. (**J**) Cardiomyocytes labeled with BODIPY 581/591 C11 after cold ischemia. Scale bars: 25 μm. (**K**) Levels of MDA in cardiomyocytes after cold ischemia. (**L**) 4-HNE staining of cardiomyocytes after cold ischemia. Scale bars: 25 μm. (**M**) Western blotting and quantification of ACSL4 and PTGS2 in cardiomyocytes after cold ischemia. (**N**) Cell viability of cardiomyocytes after cold ischemia. (**O**) Live-cell imaging of cardiomyocytes incubated with SYTOX Green after cold ischemia. Scale bars: 300 μm. (**P**) Time-lapse analysis of cell death in cardiomyocytes after cold ischemia. SG, SYTOX green. Data shown are the mean ± standard deviation, with individual values presented as a dot plot. *P < 0.05, *P < 0.01, and ***P < 0.001, by Student’s *t* test.

To further examine the effect of CIRBP on ferroptosis susceptibility in cardiomyocytes during heart transplantation, we established an in vitro model of cold ischemia to simulate cold storage in heart transplantation and evaluated ferroptosis-related markers during this process (Figure 6A).

Cardiomyocytes were isolated from WT or *Cirbp*-KO neonatal rats and subjected to cold ischemia. Mito-BODIPY staining revealed significantly increased C11 oxidation in *Cirbp*-KO cardiomyocytes compared with WT cardiomyocytes. Moreover, the levels of 4-HNE and MDA were significantly increased in *Cirbp*-KO cardiomyocytes compared with WT cardiomyocytes. These results suggested that cellular lipid peroxidation was increased in *Cirbp*-KO cardiomyocytes after cold ischemia (Figure 6, B–D). Western blot analysis revealed that the expression of ferroptosis-related molecular markers, including acyl-CoA synthetase long chain family member 4 (ACSL4) and PTGS2, was significantly increased in *Cirbp*-KO cardiomyocytes compared with WT cardiomyocytes (Figure 6E), indicating that ferroptosis was triggered in *Cirbp*-KO cardiomyocytes after cold ischemia. Finally, *Cirbp*-KO cardiomyocytes exhibited significantly decreased viability and increased cell death after cold ischemia compared with WT cardiomyocytes (Figure 6, F–H).

In addition, we isolated cardiac fibroblasts from WT or *Cirbp*-KO neonatal rats and evaluated their susceptibility to ferroptosis after cold ischemia. Ferroptosis-related marker expression and cell viability were comparable between the 2 groups, which suggested that the effect of CIRBP on ferroptosis susceptibility after cold ischemia was more specific to cardiomyocytes (Supplemental Figure 14).

Furthermore, we treated *Cirbp*-KO cardiomyocytes with a ferroptosis inhibitor (liproxstatin-1, 5 μM) during cold ischemia (Figure 6I), and the levels of C11 oxidation, 4HNE and MDA, as well as the expression of ferroptosis-related molecular markers were significantly lower in the treatment group than in the control group. These findings indicated that ferroptosis was alleviated in *Cirbp*-KO cardiomyocytes after liproxstatin-1 treatment (Figure 6, J–M). Importantly, compared with the control group, the treatment group exhibited significantly increased cell viability and decreased cell death, which demonstrated a causal relationship between ferroptosis and cellular injury after cold ischemia in *Cirbp*-KO cardiomyocytes (Figure 6, N–P).

Taken together, these in vitro results suggested that CIRBP played an important role in protecting cardiomyocytes from ferroptosis after cold ischemia.

### Cirbp overexpression enhances the hypothermic cardioprotection of aged donor hearts during cold storage in transplantation.

**Figure 7:**
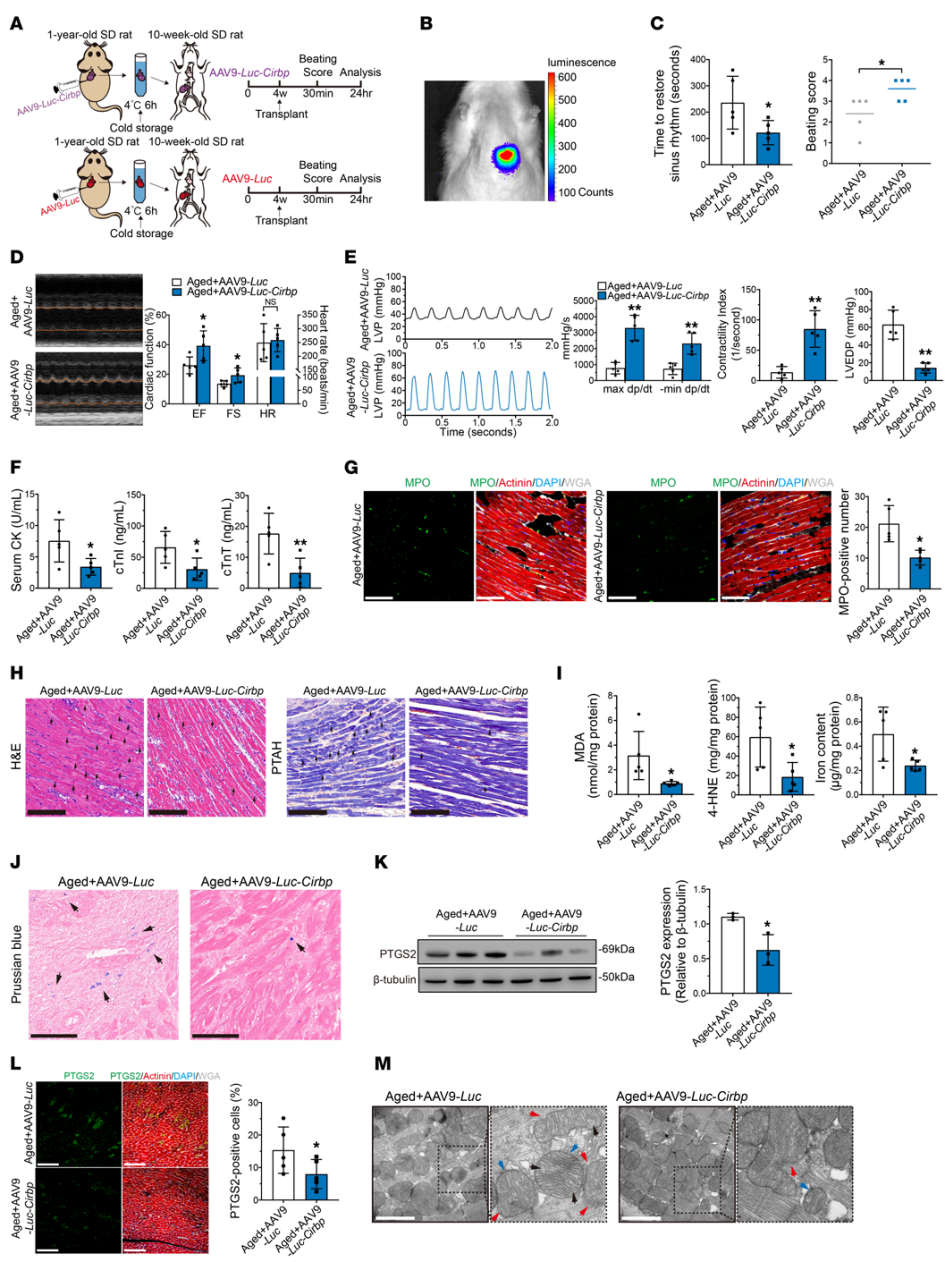
Overexpression of *Cirbp* enhances hypothermic cardioprotection in aged donor hearts during cold storage in transplantation. (**A**) Experimental design. Donor hearts were harvested from aged rats injected with AAV9-*Luc*-*Cirbp* or AAV9-*Luc* (*n* = 5 in each group) and transplanted into young rats. Hypothermia was used to protect the donor heart during cold storage. (**B**) In vivo imaged of luciferase in aged rats after AAV9 transfection. (**C**) Assessment of cardiac resuscitation after transplantation. (**D**) Cardiac function of donor hearts measured by echocardiography after transplantation. (**E**) Representative LVP traces and cardiac function parameters of donor hearts determined via cardiac catheterization after transplantation. (**F**) Serum cardiac enzymes in recipients after transplantation. (**G**) MPO staining of donor hearts after transplantation. Scale bars: 50 μm. (**H**) H&E and PTAH staining of donor hearts after transplantation. Arrows indicate myocardial contraction band necrosis. Scale bars: 100 μm (H&E); 50 μm (PTAH). (**I**) Peroxidation parameters and iron overload in donor hearts after transplantation. (**J**) Prussian blue staining of donor hearts after transplantation. Arrows indicate iron deposition in myocardium. Scale bars: 50 μm. (**K**) Western blotting and quantification of PTGS2 in donor hearts after transplantation. (**L**) Immunofluorescence staining of PTGS2 in donor hearts after transplantation. Scale bars: 100 μm. (**M**) TEM images of donor hearts after transplantation. Arrows indicate mitochondrial shrinkage (blue), crista loss (black), and membrane rupture (red). Scale bars: 1 μm. Quantitative data are shown as the mean ± standard deviation, with individual values presented as a dot plot. Statistical significance of beating score was determined with Mann-Whitney *U* test. Statistical significance of the other parameters was determined with a 2-sided Student’s *t* test (*P < 0.05 and **P < 0.01).

To demonstrate that reduced *Cirbp* expression is responsible for the attenuation of hypothermic cardioprotection in aged donor hearts during cold storage, we used AAV9 to induce overexpression of myocardial *Cirbp* in aged rats (1 year old). An AAV9 vector carrying the cTNT promoter to drive the expression of *Cirbp* and firefly luciferase (AAV9-*Luc*-*Cirbp*) was injected into aged rats, and an AAV9 vector carrying firefly luciferase (AAV9-*Luc*) was used as a control. After 4 weeks, bioluminescence imaging confirmed the successful transfection of AAV9 in the heart of aged rats. Then, the hearts were harvested and transplanted into young rats (10 weeks old) after 6 hours of cold storage (Figure 7, A and B). Compared with the controls, aged AAV9-*Luc*-*Cirbp* donor hearts took less time to return to sinus rhythm and had increased beating scores after transplantation (Figure 7C). Moreover, compared with the controls, aged AAV9-*Luc*-*Cirbp* donor hearts had increases in EF, FS, max dp/dt, min dp/dt, CI and a decrease in LVEDP on day 1 after transplantation (Figure 7, D and E). In addition, 7 days after transplantation, aged AAV9-*Luc*-*Cirbp* donor hearts also exhibited significantly improved cardiac function compared with that of the controls (Supplemental Figure 15). These results suggested that significant improvements in postoperative cardiac function were achieved by overexpressing *Cirbp* in aged donor hearts. In addition, the serum levels of cardiac enzymes were significantly lower in recipients transplanted with aged AAV9-*Luc*-*Cirbp* donor hearts than in those transplanted with aged AAV9-*Luc* donor hearts (Figure 7F). Furthermore, MPO, H&E, and PTAH staining revealed that neutrophil infiltration and myocardial contraction band necrosis were alleviated in aged AAV9-*Luc*-*Cirbp* donor hearts (Figure 7, G and H), indicating that overexpression of CIRBP in aged donor hearts could mitigate myocardial injury during transplantation.

Notably, *Cirbp* overexpression reduced MDA, 4-HNE, and iron levels in aged donor hearts after transplantation compared with the controls (Figure 7I). Prussian blue staining also showed that iron deposition was decreased in aged AAV9-*Luc*-*Cirbp* donor hearts compared with aged AAV9-*Luc* donor hearts (Figure 7J). Western blotting and immunofluorescence staining showed that PTGS2 expression was significantly lower in aged AAV9-*Luc*-*Cirbp* donor hearts than in the controls (Figure 7, K and L). Electron microscopy suggested that ferroptosis-related mitochondrial changes were mitigated in aged AAV9-*Luc*-*Cirbp* donor hearts compared with aged AAV9-*Luc* donor hearts (Figure 7M). These results indicated that ferroptosis was partially alleviated in aged donor hearts after *Cirbp* overexpression.

Taken together, these findings suggested that *Cirbp* overexpression could improve hypothermic cardioprotection and ameliorate ferroptosis in aged donor hearts after transplantation.

### CIRBP facilitates the translation of Dhodh mRNA to protect the donor heart against ferroptosis after transplantation.

**Figure 8:**
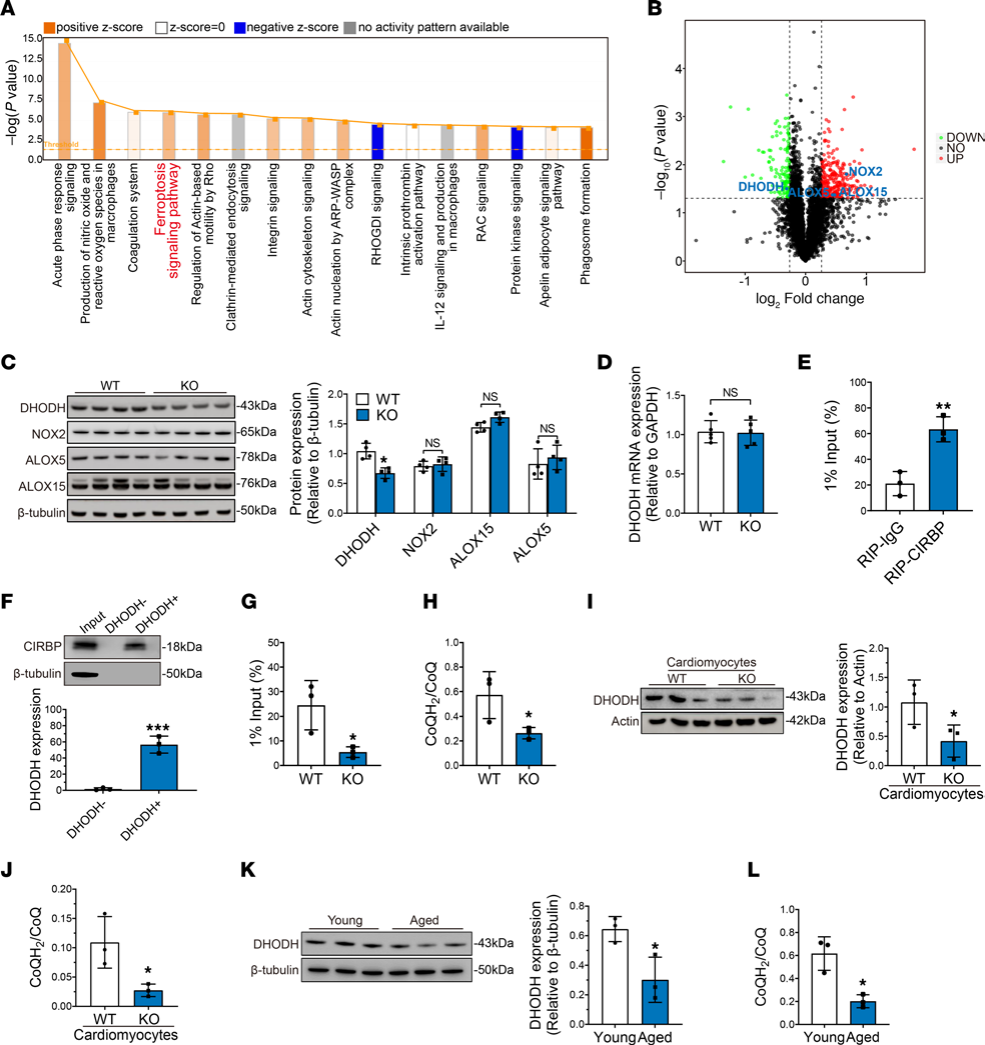
CIRBP binds *Dhodh* mRNA and enhances its translation. (**A**) Proteomics analysis of donor hearts harvested from WT rats and *Cirbp*-KO rats was performed. IPA of differentially expressed proteins between the WT and KO groups. (**B**) Volcano plots showing the differentially expressed proteins between the WT and KO groups. Green dots represent genes whose expression were significantly decreased in KO groups. Red dots represent genes whose expression were significantly increased in KO groups. Gray dots represent genes whose expression did not reach the statistical significance. (**C**) Verification of significantly differentially expressed proteins that are involved in the ferroptosis signaling pathway between WT and KO groups after transplantation. (**D**) Real-time qPCR analysis of *Dhodh* mRNA expression in donor hearts from the WT and KO groups after transplantation. (**E**) The binding of CIRBP to *Dhodh* mRNA was investigated by ultraviolet cross-link RIP assay. (**F**) Biotin-pulldown assays were performed using biotinylated fragments of *Dhodh* mRNA to detect bound cellular CIRBP. (**G**) RNC mRNA and total RNA were isolated from donor hearts after transplantation and were both subjected to real-time qPCR analysis to assess the presence of *Dhodh* mRNA. The *Dhodh* mRNA in total RNA was used as input. (**H**) CoQH_2_/CoQ ratios in donor hearts from the WT and KO groups after transplantation. (**I**) Western blotting and quantification of DHODH in WT and *Cirbp*-KO cardiomyocytes after cold ischemia. (**J**) CoQH_2_/CoQ ratios in WT and *Cirbp*-KO cardiomyocytes after cold ischemia. (**K**) Western blotting and quantification of DHODH in young and aged donor hearts after transplantation. (**L**) CoQH_2_/CoQ ratios in young and aged donor hearts after transplantation. Quantitative data are shown as the mean ± standard deviation, with individual values presented in a dot plot. *P < 0.05, **P < 0.01, and ***P < 0.001, by 2-sided Student’s *t* test.

To determine how hypothermic cardioprotection is impaired in young *Cirbp*-KO donor hearts during cold storage, we collected heart tissues from the WT and *Cirbp*-KO donor hearts after transplantation and analyzed their proteomes. The results revealed that a total of 378 proteins had significant differences between the 2 groups (fold change < 0.83 or > 1.20; *P* < 0.05) (Supplemental Table 2). PCA revealed the apparent separation of clusters in the *Cirbp*-KO and WT groups (Supplemental Figure 16). Ingenuity Pathway Analysis (IPA) revealed that the ferroptosis signaling pathway was significantly perturbed in young *Cirbp*-KO donor hearts (Figure 8A). On the basis of these results, we performed further analysis of differentially expressed proteins and identified 4 proteins involved in the ferroptosis signaling pathway. Although proteomics analysis revealed significant differences in the expression of these 4 proteins in the *Cirbp*-KO group compared with the WT group, Western blot analysis revealed a significant decrease in the expression of only DHODH (Figure 8, B and C). Additionally, we isolated cardiomyocytes from young and aged human donor hearts and subjected them to cold ischemia. Aged human cardiomyocytes also had significantly decreased levels of DHODH after cold ischemia compared with levels in young human donor hearts (Supplemental Figure 17).

A recent study reported that *Dhodh* depletion could lead to ferroptosis in the presence of low glutathione peroxidase 4 (GPX4) expression (20). Thus, we evaluated the expression of GPX4 in donor hearts during transplantation. Western blot analysis revealed that GPX4 expression was significantly lower in both young and aged donor hearts after transplantation than before transplantation (Supplemental Figure 18, A and B), although its expression after transplantation was comparable between the 2 groups (Supplemental Figure 18C). In addition, GPX4 stands for GPX4 expression was lower in cardiomyocytes after cold ischemia than before cold ischemia (Supplemental Figure 18D). These results suggested that transplantation disrupted GPX4 expression, which permitted DHODH to induce ferroptosis in aged donor hearts.

We used AAV9 to drive overexpression of *Dhodh* (AAV9-*Luc*-*Dhodh*) in young *Cirbp*-KO donor hearts and found that cardiac function and myocardial ferroptosis were partially rescued after transplantation compared with donor hearts treated with the vector (AAV9-*Luc*) (Supplemental Figure 19), indicating that DHODH was a major downstream effector of CIRBP that protected donor hearts from ferroptosis after transplantation.

Next, using real-time qPCR, we measured *Dhodh* mRNA expression in donor hearts after transplantation. Interestingly, we found no significant differences between the WT and *Cirbp*-KO groups (*P* = 0.90) (Figure 8D), indicating that CIRBP modulated *Dhodh* mRNA at the posttranscriptional level. Because CIRBP belongs to the RNA-binding protein family (21), and we identified 4 sequences that matched with the CIRBP-binding motif in the 3′-UTR region of *Dhodh* mRNA (Supplemental Table 3), we investigated whether CIRBP could bind *Dhodh* mRNA by performing an RNA immunoprecipitation (RIP) assay. Compared with expression in the negative control group, the enrichment of *Dhodh* mRNA expression was significantly greater in ribonucleoprotein complexes that immunoprecipitated with the anti-CIRBP antibody, demonstrating that CIRBP could specifically bind *Dhodh* mRNA in vivo (Figure 8E). The interaction between CIRBP and *Dhodh* mRNA was further validated by RNA-pulldown analysis with a biotin-conjugated probe and whole-tissue lysates, and the results revealed that a probe bearing 3′-UTR *Dhodh* mRNA fragments interacted with CIRBP (Figure 8F). We used qPCR to analyze translating mRNAs (ribosome-nascent chain complex–bound mRNA [RNC mRNAs]) in the donor hearts after transplantation. *Dhodh* mRNA levels were significantly lower in RNC mRNAs from the *Cirbp*-KO group than from the WT group (Figure 8G). These results indicated that CIRBP could bind *Dhodh* mRNA and facilitate its translation.

DHODH can reduce ubiquinone (CoQ) to ubiquinol (CoQH_2_), which suppresses mitochondrial lipid peroxidation and prevents subsequent ferroptosis (20). Thus, we measured the ratio of CoQH_2_ to CoQ in donor hearts after transplantation by ultra-high-performance liquid chromatography–multiple reaction monitoring–MS/MS (UHPLC-MRM-MS/MS) analysis. Compared with that in the WT donor hearts, the ratio in young *Cirbp*-KO donor hearts was significantly decreased (Figure 8H). Similarly, compared with WT cardiomyocytes, *Cirbp*-KO cardiomyocytes exhibited significantly decreased expression of DHODH and a significantly reduced ratio of CoQH_2_ to CoQ after cold ischemia (Figure 8, I and J). Similar results were found in aged donor hearts after transplantation compared with young donor hearts (Figure 8, K and L). Moreover, compared with the vector control group, the expression of DHODH and the ratio of CoQH_2_ to CoQ was significantly increased in *Cirbp*-overexpressing aged donor hearts after transplantation (Supplemental Figure 20).

In conclusion, these data suggested that CIRBP promotes the translation of *Dhodh* mRNA and increases the proportion of CoQH_2_ in the myocardium, which protects donor hearts from ferroptosis after transplantation.

### Cirbp agonist treatment improves the hypothermic cardioprotection of aged donor hearts during cold storage in transplantation.

**Figure 9:**
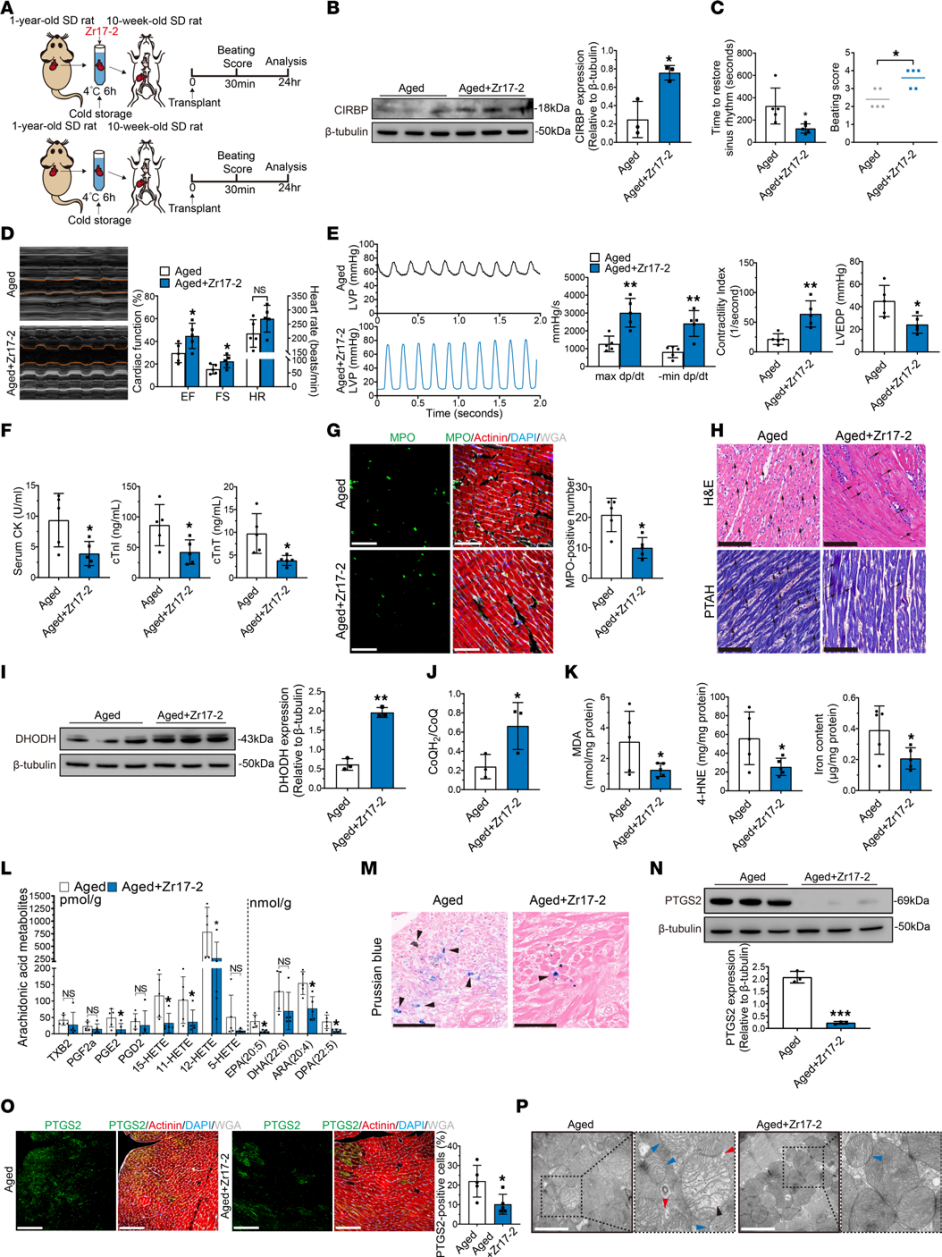
Cardioplegic solution supplemented with *Cirbp* agonist improves hypothermic cardioprotection of aged donor hearts during cold storage in transplantation. (**A**) Experimental design. Aged donor hearts were harvested and transplanted into young rats. UW solution was used to induce cardiac arrest in the control group, and UW solution supplemented with the *Cirbp* agonist Zr17-2 was used in the experimental group (*n* = 5 in each group). Hypothermia was used to protect donor hearts during cold storage. (**B**) Western blotting and quantification of CIRBP in donor hearts after transplantation. (**C**) Assessment of cardiac resuscitation after transplantation. (**D**) Cardiac function of donor hearts measured by echocardiography after transplantation. (**E**) Representative LVP traces and cardiac function parameters of donor hearts determined via cardiac catheterization after transplantation. (**F**) Serum cardiac enzymes in recipients after transplantation. (**G**) MPO staining of donor hearts after transplantation. Scale bars: 50 μm. (**H**) H&E and PTAH staining of donor hearts after transplantation. Arrows indicate myocardial contraction band necrosis. Scale bars: 100 μm (H&E images); 50 μm (PTAH images). (**I**) Western blotting and quantification of DHODH in donor hearts after transplantation. (**J**) CoQH_2_/CoQ ratio in donor hearts after transplantation. (**K**) Peroxidation parameters and iron overload in donor hearts after transplantation. (**L**) Arachidonic acid metabolite levels in donor hearts after transplantation. (**M**) Prussian blue staining of donor hearts after transplantation. Arrows indicate iron deposition in myocardium. Scale bars: 50 μm. (**N**) Western blotting and quantification of PTGS2 in donor hearts after transplantation. (**O**) Immunofluorescence staining for PTGS2 in donor hearts after transplantation and quantification. Scale bars: 100 μm. (**P**) TEM images of donor hearts after transplantation. Arrowheads indicate mitochondrial shrinkage (blue), crista loss (black), and membrane rupture (red). Scale bars: 1 μm. Original magnification (enlarged insets), ×100,000. Data shown are the mean ± standard deviation, with individual values presented as a dot plot. *P < 0.05, **P < 0.01, and ***P < 0.001, by 2-sided Student’s *t* test.

On the basis of the finding that the decrease in *Cirbp* expression was responsible for the attenuation of hypothermic cardioprotection in aged donor hearts during cold storage, we added a *Cirbp* agonist (Zr17-2, 36 μg/100 mL) to the UW solution and evaluated its cardioprotective effects. Donor hearts were harvested from aged (1-year-old) rats, and arrest was induced with UW solution or a Zr17-2–supplemented UW solution. Then, the donor hearts were transplanted into young rats (10 weeks old) after 6 hours of cold storage (Figure 9A). Western blot analysis revealed that CIRBP expression was significantly increased in aged donor hearts after the addition of Zr17-2 (Figure 9B).

In aged donor hearts, the addition of Zr17-2 to the cardioplegic solution significantly shortened the time to restore sinus rhythm and improved the beating score after transplantation (Figure 9C). This agonist also significantly improved the postoperative cardiac function of aged donor hearts 1 day and 7 days after transplantation, as indicated by the significant increases in EF, FS, max dp/dt, min dp/dt, and CI and the decrease in LVEDP (Figure 9, D and E, and Supplemental Figure 21). Moreover, the addition of Zr17-2 to the cardioplegic solution significantly alleviated the changes in serum cardiac enzymes in the recipients that were transplanted with aged donor hearts (Figure 9F). Furthermore, MPO, H&E, and PTAH staining revealed that neutrophil infiltration and myocardial contraction band necrosis were alleviated after the addition of Zr17-2 (Figure 9, G and H), indicating that Zr17-2 mitigated myocardial injury in aged donor hearts after transplantation.

In addition, Zr17-2 significantly increased the expression of DHODH and the ratio of CoQH_2_ to CoQ in aged donor hearts after transplantation (Figure 9, I and J). As a result, the levels of MDA, 4-HNE, iron, and arachidonic acid metabolites were significantly decreased in aged donor hearts after transplantation (Figure 9, K and L). Prussian blue staining also showed that iron deposition was decreased in aged donor hearts after Zr17-2 supplementation (Figure 9M). Finally, Zr17-2 significantly decreased the expression of PTGS2 in aged donor hearts (Figure 9, N and O), and electron microscopy showed that ferroptosis-related mitochondrial changes in aged donor hearts were mitigated by the addition of Zr17-2 (Figure 9P). Notably, the Seahorse assay showed that Zr17-2 significantly increased mitochondrial function in aged donor hearts (Supplemental Figure 22). These results indicated that ferroptosis was alleviated in aged donor hearts after Zr17-2 supplementation.

Taken together, these results indicated that applying a cardioplegic solution supplemented with Zr17-2 could improve the cardioprotective effects of hypothermia on aged donor hearts during cold storage in transplantation.

## Discussion

**Figure 10:**
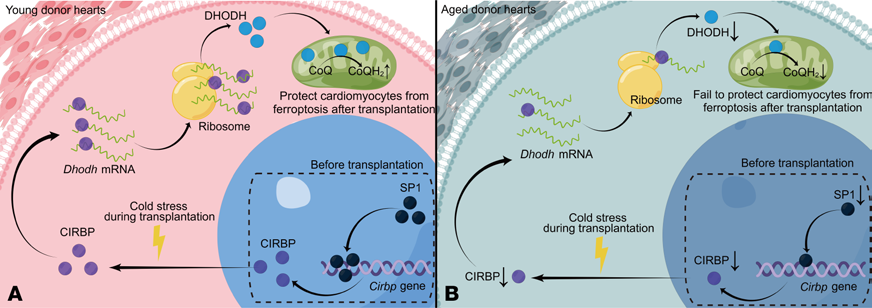
Suppression of *Cirbp* compromises DHODH-mediated ferroptosis defense and attenuates hypothermic cardioprotection during transplantation of aged donor hearts. (**A**) In the young donor heart, nuclear SP1 binds to the promoter region of *Cirbp* and induce *Cirbp* expression in cardiomyocytes at baseline. Then, during cold storage in heart transplantation, CIRBP is activated by cold stress and translocates from the nucleus to the cytoplasm. CIRBP binds to *Dhodh* mRNA and facilitates its translation. Thereafter, increased DHODH enhances the reduction of CoQ to CoQH_2_, which protects the donor heart against ferroptosis after transplantation. (**B**) In the aged donor heart, nuclear abundance of SP1 is decreased, which inhibits *Cirbp* expression in cardiomyocytes. Consequently, DHODH-mediated ferroptosis defense is compromised during heart transplantation, resulting in attenuated hypothermic cardioprotection in the aged donor heart.

Numerous studies have reported unsatisfactory outcomes in recipient patients who were transplanted with aged donor hearts (6, 8), but the mechanisms underlying these outcomes have remained unclear. Here, we identified a mechanism that links aging with compromised hypothermic cardioprotection and exacerbated ferroptosis in aged donor hearts after transplantation (Figure 10). At baseline, aging suppressed the expression of SP1 and inhibited its binding to the *Cirbp* promoter region, leading to decreased CIRBP expression in the aged myocardium. Under cold stress, CIRBP translocated from the nucleus to the cytoplasm in donor hearts during cold storage in transplantation. It could bind *Dhodh* mRNAs and facilitate their translation, which increased the reduction of CoQ to CoQH_2_ to prevent myocardial ferroptosis after transplantation. However, *Cirbp* expression was suppressed in aged donor hearts, and the abundance of translocated CIRBP was also decreased during cold storage, resulting in the attenuation of hypothermic cardioprotection; moreover, *Cirbp* overexpression in aged donor hearts could ameliorate this cardioprotection.

The aged heart undergoes a series of changes at the molecular level that leave it vulnerable to myocardial injury in cardiovascular diseases such as myocardial infarction and cardiomyopathy (22, 23). In our study, we found that the expression of SP1, which is a transcription factor that is upstream of *Cirbp*, was decreased in aged donor hearts, leading to the attenuation of hypothermic cardioprotection. Previous studies have shown that a decrease in SP1 in aged cells is associated with the age-related increases in ROS (19). Moreover, another study reported that SP1 downregulation during cellular senescence is due to an increase in the rate of SP1 protein degradation (24). These studies might explain the decreased levels of SP1 in aged donor hearts. Notably, the proteasome inhibitor ALLN can block SP1 degradation in aged cells (24). Thus, this inhibitor might also have the potential to improve the cardioprotective effect of hypothermia on aged donor hearts.

In our study, we found that decreasing the expression of SP1 suppressed CIRBP expression in aged donor hearts. Using *Cirbp*-KO and *Cirbp*-overexpressing donor hearts, we further demonstrated the pivotal role of CIRBP in hypothermic cardioprotection during cold storage in heart transplantation. A previous study showed that CIRBP could enhance CoQ biosynthesis to protect the heart from apoptosis during cardiopulmonary bypass (11), but our study showed that CIRBP protected the donor heart by reducing CoQ to CoQH_2_ during transplantation, indicating the multiple mechanisms by which CIRBP regulates CoQ. Interestingly, a recent study reported that CIRBP promotes ferroptosis during renal ischemia-reperfusion injury, but our study showed that CIRBP inhibited ferroptosis during heart transplantation, suggesting that CIRBP might play distinct roles in the regulation of ferroptosis under different pathophysiological conditions. Importantly, we found that the CIRBP agonist Zr17-2 could enhance CIRBP expression in aged donor hearts, thereby increasing the reduction of CoQ to protect cardiomyocytes against ferroptosis. Therefore, Zr17-2 supplementation might be a promising strategy to protect aged donor hearts during transplantation.

Ferroptosis has become a popular research topic in the field of cardiovascular diseases, but its role in heart transplantation has not been fully elucidated. The DHODH and GPX4 systems are 2 major ferroptosis defense systems; deficiencies in either system can cause a cell to become dependent on the other, and disabling both systems can trigger ferroptosis induced by mitochondrial lipid peroxidation (20). In our study, in both young and aged donor hearts, we found that GPX4-mediated ferroptosis defense was compromised during transplantation. Importantly, we found that DHODH expression was decreased in aged donor hearts because of aging-induced CIRBP suppression. Therefore, ferroptosis exacerbation was evident in aged donor hearts after transplantation. Notably, we found that the ferroptosis inhibitors liproxstatin-1 and ferrostatin-1 could attenuate myocardial injury in aged donor hearts during transplantation, suggesting that these inhibitors could also be a therapeutic tool in the future.

This study has several limitations. First, it was based on a heterotopic heart transplantation model because orthotopic heart transplantation is still hardly performed on rats due to limitations in surgical technology. Although echocardiography and cardiac catheterization were used to evaluate cardiac functions of heterotopic transplanted hearts, we were unable to evaluate their functions in a normal loading state because blood flow from the pulmonary vein to the left atrium in the heterotopic transplanted heart is absent, resulting in its unloading state. Second, the human myocardial specimens used in this study were harvested from nontransplanted human donor hearts because postoperative samples are difficult to acquire due to ethical concerns. Third, the aged donor hearts used in this study were harvested from 1-year-old rats. One-year-old rats physiologically correspond to the 30- to 40-year-old human age range, which is consistent with the previously reported age limit for decreased donor heart performance (25). Although we found that *Cirbp* suppression was progressive in older donor hearts, further exploration is needed to determine whether the attenuation of hypothermic cardioprotection will persist as age increases. Finally, we used syngeneic rats to establish a heart transplantation model; therefore, transplant rejection was not significant, and we did not use immunosuppressants. Whether transplant rejection is related to severe myocardial injury in aged donor hearts and whether the effectiveness of immunosuppression is compromised in aged donor hearts are important questions that require further research.

Our findings have strong clinical implications. According to a report from the Organ Procurement Transplant Network (2), approximately 4,000 new candidates are added to the heart transplant waiting list every year in the United States, and the number of candidates awaiting a heart transplant has increased tremendously in the past 10 years. Therefore, we must increase the donor age to meet the growing demand for heart transplantations. Although normothermic preservation has been used in heart transplantation, traditional cold storage continues to be a prevalent method for this surgery, especially in developing countries, due to its accessibility and cost effectiveness (26). Our findings can improve hypothermic cardioprotection in aged donor hearts and broaden the indications for the use of aged donor hearts for transplantation. Moreover, hypothermia is also used in other clinical scenarios, including the protection of donor livers and donor kidneys during organ transplantation. For patients who undergo transplantation with these organs from aged donors, the protective effects of hypothermia might also be compromised. Therefore, our findings could be extended to these contexts and improve the hypothermic protection of other organs from aged donors.

## Methods

Detailed methods are available in the Supplemental Methods.

### Sex as a biological variable.

The animal study exclusively examined male rat to reduce the confounding influence of sex hormones. It is unknown whether the findings are similar for female rat.

### Study design.

The purpose of this study was to explore the mechanism of the attenuation of hypothermic cardioprotection in aged donor hearts during cold storage in heart transplantation and to investigate its translational value. Using a rat model of heart transplantation, we performed RNA-Seq to attempt to identify the most relevant protein to explain the attenuation of hypothermic cardioprotection in aged donor hearts. Moreover, changes in expression of the identified protein were verified in aged human donor hearts. Furthermore, we used genetically modified rats to validate the central role of the identified protein and its downstream effectors. In the animal study, the grouping criteria were based on the age and genotype of the donor rats. The sample size was determined on the basis of previous reports and our past experience using this animal model. The detailed experimental design is shown in the flowchart (Supplemental Figure 23).

### Statistics.

Statistical analysis was performed using SPSS 22.0 software (SPSS Inc.). The normality of the distributions was evaluated with the Shapiro-Wilk test. For 2-group comparisons, Student’s *t* test was used for normally distributed data, and the Mann-Whitney *U* test was used for non-normally distributed data. To evaluate the differences among 3 or more groups, 1-way ANOVA followed by a Tukey-Kramer multiple-comparison test was used. All *P* values are 2 sided, and *P* values of less than 0.05 were considered to indicate statistical significance. The data are expressed as the mean ± standard deviation.

### Study approval.

Informed consent was obtained from the families of all the donors. The human studies conformed to the principles outlined in the Declaration of Helsinki and were approved by the Shanghai Children’s Medical Center Research Ethics Committee (SCMCIRB-K2022119-1). All experiments involving animals were conducted in accordance with the *Guide for the Use and Care of Laboratory Animals* (National Academies Press, 2011), and all animal protocols were approved by the Shanghai Children’s Medical Center Animal Care and Use Committee (SCMC-LAWEC-2020-010).

### Data availability.

The RNA-Seq and ChIP-Seq data are available from the NCBI’s Gene Expression Omnibus (GEO) database (GEO GSE227005). The mass spectrometry proteomics data have been deposited in the ProteomeXchange Consortium via the iProX partner repository with the data set identifier PXD044088. All data supporting the graphs and tables are provided in the Supporting Data Values file. The other data and study materials are available to researchers for the purposes of reproducing the results or replicating the procedure upon request to the corresponding authors.

## Author contributions

YZ, HZ, and Y Liu conceived and designed the study. YZ and CJ performed the animal study and analyzed the data. JH performed sequencing experiments, bioinformatics analyses, and statistical analyses. CH and XH collected the human samples. XZ and YS were responsible for visualization. LW, Y Li, and BF provided technical advice and performed the rat histopathology. YY and JL provided consultation and material support. YZ and CJ drafted the manuscript. Y Liu and HZ are the principal investigators, obtained funding, and assisted with the drafting of the manuscript. All the authors reviewed, contributed to, and approved the final manuscript.

## Supplementary Material

Supplemental data

Unedited blot and gel images

Supporting data values

## Version 1 05/01/2024

Electronic publication

## References

[1] Tanai E (2015). Pathophysiology of heart failure. Compr Physiol.

[2] Colvin M (2022). OPTN/SRTR 2020 annual data report: heart. Am J Transplant.

[3] Axtell AL (2019). The effect of donor age on posttransplant mortality in a cohort of adult cardiac transplant recipients aged 18-45. Am J Transplant.

[4] Sathianathan S (2022). Heart transplant donor selection guidelines: review and recommendations. Curr Cardiol Rep.

[5] Khasati NH (2007). Donor heart selection: the outcome of “unacceptable” donors. J Cardiothorac Surg.

[6] Khush KK (2020). International Society for HeartLung Transplantation. The International Thoracic Organ Transplant Registry of the International Society for Heart and Lung Transplantation: 37th adult heart transplantation report-2020; focus on deceased donor characteristics. J Heart Lung Transplant.

[7] DeFilippis EM (2022). Evolving characteristics of heart transplantation donors and recipients: JACC focus seminar. J Am Coll Cardiol.

[8] John MM (2019). Interaction between ischemic time and donor age on adult heart transplant outcomes in the modern era. Ann Thorac Surg.

[10] Copeland H (2020). Donor heart and lung procurement: A consensus statement. J Heart Lung Transplant.

[11] Liu Y (2019). Chronic hypoxia-induced *Cirbp* hypermethylation attenuates hypothermic cardioprotection via down-regulation of ubiquinone biosynthesis. Sci Transl Med.

[12] Jackson TC (2018). Infants uniquely express high levels of RBM3 and other cold-adaptive neuroprotectant proteins in the human brain. Dev Neurosci.

[13] Ono K (1969). Improved technique of heart transplantation in rats. J Thorac Cardiovasc Surg.

[14] Dixon SJ (2012). Ferroptosis: an iron-dependent form of nonapoptotic cell death. Cell.

[15] Yang WS (2014). Regulation of ferroptotic cancer cell death by GPX4. Cell.

[16] Liao Y (2017). The role of cold-inducible RNA binding protein in cell stress response. Int J Cancer.

[17] Zhang HT (2015). Cold-inducible RNA-binding protein inhibits neuron apoptosis through the suppression of mitochondrial apoptosis. Brain Res.

[18] Araki R (2015). DNA methylation of the GC box in the promoter region mediates isolation rearing-induced suppression of srd5a1 transcription in the prefrontal cortex. Neurosci Lett.

[19] Chhunchha B (2018). Sumoylation-deficient Prdx6 repairs aberrant Sumoylation-mediated Sp1 dysregulation-dependent Prdx6 repression and cell injury in aged and oxidative stress. Aging (Albany NY).

[20] Mao C (2021). DHODH-mediated ferroptosis defence is a targetable vulnerability in cancer. Nature.

[21] Sui M (2021). CIRBP promotes ferroptosis by interacting with ELAVL1 and activating ferritinophagy during renal ischaemia-reperfusion injury. J Cell Mol Med.

[22] Dhalla NS (2012). Cardiac remodeling and subcellular defects in heart failure due to myocardial infarction and aging. Heart Fail Rev.

[23] Yáñez-Bisbe L (2021). Aging impairs reverse remodeling and recovery of ventricular function after isoproterenol-induced cardiomyopathy. Int J Mol Sci.

[24] Oh JE (2007). Downregulation of transcription factor, Sp1, during cellular senescence. Biochem Biophys Res Commun.

[25] Khush KK (2020). The International Thoracic Organ Transplant Registry of the International Society for Heart and Lung Transplantation: 37th adult heart transplantation report – 2020 focus on deceased donor characteristics. J Heart Lung Transplant.

[26] Lechiancole A (2023). Graft preservation in heart transplantation: current approaches. Front Cardiovasc Med.

