# Supplementary material for: Cirbp suppression compromises DHODH-mediated ferroptosis defense and attenuates hypothermic cardioprotection in an aged donor transplantation model
Source: J Clin Invest. 2024 Mar 12;134(9):e175645. doi: 10.1172/JCI175645 (PMC11060748; doi:10.1172/JCI175645)
Supplement: Supplemental data [file jci-134-175645-s036.pdf]

Supplementary Materials

Cirbp suppression compromises DHODH-mediated ferroptosis defense and attenuates hypothermic cardioprotection in an aged-donor transplantation model

Yifan Zhu; Chenyu Jiang; Jian He; Chen He; Xingliang Zhou; Xu Huang; Yi Shen; Liwei Wu; Yongnan Li; Bei Feng; Yi Yan; Jun Li; Hao Zhang; Yiwei Liu

Materials and Methods

Rat model of heart transplantation

Male adult Sprague Dawley rats were obtained from Vital River Laboratory (Beijing, China). All animal experiments were conducted in accordance with the *Guide for the Use and Care of Laboratory Animals* (National Academies Press, 2011), and all animal protocols were approved by the Shanghai Children's Medical Center Animal Care and Use Committee (SCMC-LAWEC-2020-010). The rat model of heart transplantation was established as previously described (1). The grouping criteria were based on the age and genotype of the donor rats. The sample size was determined on the basis of previous reports and our past experience using this animal model. For the *Cirbp* agonist experiment, animals were randomized into treatment or control group using simple randomization method. The principal researchers were blinded from the group allocations when functional experiments were conducted. To eliminate the influence of estrogen on hypothermic cardioprotection, we only adopted male rats in animal studies. Detailed protocol was described below. The experiments were conducted under room temperature. The donor hearts were harvested from 10-week-old or 1-year-old male rats. Each rat was anesthetized with isoflurane (5%) and maintained by the mask (3% with an O_2 flow of 1 L/min). After performing a median laparotomy, 500 IU heparin dissolved in 1 ml saline were injected into the inferior vena cava for anticoagulation, then the diaphragm was incised and lateral thoracotomy was performed on both sides. The donor heart was exsanguinated by transecting the abdominal vessels. After infusion of UW solution (CoStorSol ®, University of Wisconsin), the ascending aorta and pulmonary artery were separated through the transvers pericardial sinus. Ligate the superior and inferior vena cava and the pulmonary veins and extract the heart. The donor heart was further perfused by heparin saline and UW solution, and was store at 4℃ for 6 hours. Then, 10-week-old male rats were used as recipient. The anesthesia procedures and median laparotomy were performed on recipient rat. The intestines were moved to the left side outside the abdomen and placed on a moist compress. Blunt dissection of adipose tissue from abdominal aorta and inferior vena cava below renal vessel level. Elevate the abdominal vessels and position the Cooley vascular clamp on it. Then a longitudinal incision was created on the vessels and it was flushed with heparin saline. Anastomose the ascending aorta of the donor heart with the abdominal aorta of the recipient, and then anastomose the pulmonary artery of the donor heart with inferior vena cava of the recipient with 8-0 running sutures. The donor heart gradually resumed sinus rhythm after releasing the Cooley vascular clamp. The time from the beginning of donor reperfusion to its recovery of sinus rhythm was recorded. After 30 min of reperfusion, the beating score was assessed according to the Stanford cardiac surgery laboratory graft scoring system (0: no contraction; 1: contraction barely palpable; 2: obvious decrease in contraction strength, but still contracting in a coordinated manner, rhythm disturbance; 3: strong, coordinated beat but noticeable decrease in strength or rate, distention/stiffness; or 4: strong contraction of both ventricles, regular rate, no enlargement or stiffness). At 1 or 7 days after transplantation,

cardiac catheterization was performed, during which a stab wound in heart apex was made in transplanted hearts with 28-gauge needle. Then a 2F Millar catheter (Millar Inc, Houston) was inserted though the wound and the hemodynamic performance was recorded. The data were analyzed using LabChart 8.0 software (AD INSTRUMENTS, Australia).

Human cardiac specimens

The human studies conformed to the principles outlined in the Declaration of Helsinki and were approved by Shanghai Children's Medical Center Research Ethics Committee (SCMCIRB-K2022119-1). Cardiac specimens from human donor hearts were obtained and provided by Organ Procurement Organizations in Guangxi province (n=7, <35 years old; and n=5, >35 years old). Due to remote locations, suitable heart recipients could not be found for these donor hearts. Left ventricle tissues (approximately 500 mg net weight) were harvested after the organ donors were diagnosed with brain death. Informed consent was obtained from the families of all donors and they agreed with that these specimens could be used for scientific research. The patient characteristics are summarized in Supplemental Table 1.

Generation of *Cirbp*-KO rats

Cirbp-KO rats were produced by using transcription activator-like effector nuclease (TALEN)-based genome editing techniques. TALEN constructs specific for the rat *Cirbp* gene were designed to target its exon (Repeat variable diresidue: TGCCTCTCTCCTGCAGTGGTggtggtaaaggacagggAGACTCAACGATC CCGA; Rat Genome Database ID: 620756; Chr7: 12571310-12575127 reverse strand). TAL Effector Nucleotide Targeter 2.0 (https://tale-nt.cac.cornell.edu/) did not predict off-target site. TALEN expression vector was constructed by incorporating the TALEN construct into pCDNA3.1-TALEN plasmid. The expression vector was tested for integrity and then linearized and transcribed into mRNA and then microinjected into single-cell rat embryos from SD rats. To collect fertilized eggs, fertile female SD rats (8 to 10 weeks) were induced to super-ovulate by intraperitoneal injection of PMSG (30 IU) and hCG (30 IU). These rats were mated with fertile males and those with detected vaginal plug were selected as egg donors. Microinjected embryos were transferred to the oviduct of pseudo-pregnant rats at the day vaginal plug was detected. Genomic DNA of founder rats was extracted from tail biopsies and screened for TALEN-induced mutations at the target site of the *Cirbp* gene. TALEN target site was amplified using the following primers: forward primer 5′-AGATCTCGGAAGGTGAGGCT-3′ and reverse primer 5′-ATCCTCGGGACCGGTTAT CA-3′. Genotyping was performed by the sequencing of the PCR products from tail DNA. The founder with 7 bp deletion of the gene was chosen for further breeding. Founder rats were mated with wildtype SD males to produce F1 generation. These rats were backcrossed to SD rats for five generations, which were further intercrossed to produce the homozygous line.

Isolation of human cardiomyocytes

Human cardiomyocytes were isolated as previously described (2). In brief, cardiac tissues were collected from young and aged human donor hearts and were transported in 45 ml 4 °C University of Wisconsin (UW) solution before the isolation procedure. Once the cardiac tissues arrived at the laboratory, they were fragmented into small pieces in a Ca2+-free buffer using surgical scissors at room temperature and were then filtered into a 50-ml conical flask containing the enzymatic buffer (275 u/ml collagenase II and 1.2 u/ml protease XXIV). Supernatant was discarded and the remaining chunks were re-incubated in the same enzyme buffer without protease. Cell suspension was passed through a 100-μm filter and centrifuged at 100×g for 1 min and then cardiomyocytes were resuspended in the resuspension buffer for subsequent experiments.

Isolation of cardiomyocytes and fibroblasts from neonatal rats

The cardiomyocytes and fibroblasts were obtained from neonatal Sprague Dawley rats using neonatal heart dissociation kit (no.nc-6031, Cellutron) as previously described (3). Briefly, for each experiment, left ventricular tissues were harvest from 3 neonatal Sprague Dawley rats within 24h after birth. The tissues were minced and digested for 12 min in 10 ml enzyme buffer, then the supernatant was collected in a new tube. 4 ml of new enzyme buffer was added to the remaining tissues and digested for 15min. This digestion steps were repeated for 7-9 times until fully digested. The supernatant collection from each round underwent 1200 rpm, 1 min centrifuge to obtain pellets of digested cells. The pellet was resuspended in culture medium (no.11320033, Gibco) and plated onto 6 cm culture plates. At two hours after initial plating, non-myocyte cells attached to the dish, whereas cardiomyocytes remained in solution. Thus, the unattached cells in culture medium were transferred onto new plates to obtain cardiomyocytes. Furthermore, the attached cells underwent live/dead staining (Fixable viability stain) and fibroblast maker staining (CD90), and then flow cytometry was employed for cell sorting to obtain fibroblasts involving the following steps: gating for cells, gating for single cells, gating for live cells (Fixable viability stain) and gating for CD90-positive cells.

In vitro modeling of cold ischemia

The cold ischemia was imposed by replacing the culture medium with UW solution (CoStorSol ®, University of Wisconsin) and placing the culture plates within a humidified hypoxia incubator chamber (#27310, STEMCELL) equilibrated with 95% N_2 and 5% CO_2.Then, the chamber was placed in an incubator and the temperature was set at 4°C. After 6 h of cold ischemia, UW solution was exchanged to culture medium and the culture plates were placed in an incubator with 95% room air and 5% CO_2 at 37°C for 24 h. Then, ferroptosis-related markers and cell viability were evaluated in each group.

Cell viability assay

Cardiomyocytes and cardiac fibroblasts viability were assessed using Cell Counting Kit-8 (C0038, Beyotime) according to the manufacturer's instructions. Briefly, approximately 5,000 cells (100 μL/well) in the cell suspension were seeded in a 96-well plate. Then the plate was subjected to cold ischemia. The CCK-8 solution (10 μL) was subsequently added to each well, and the cells were incubated for indicated time. Finally, the absorbance was measured at 450 nm using a microplate reader.

BODIPY 581/591 C11 analysis

Two days before the experiment, 2×10^4 cells per well were seeded into 24-well glass bottom black plate in each well. Then, the cells were subjected to cold ischemia. After cold ischemia, the medium was removed and cells were washed once with DPBS. Cells were then labelled in 1 ml DPBS containing 5 μM BODIPY 581/591 C11 and incubated at 37 °C for 15 min. The label mixture was removed and 1 ml fresh DPBS was added to the cells. Confocal imaging and quantification of BODIPY 581/591 C11 were performed. Red and green fluorescence values were quantified using ImageJ. The BODIPY 581/591 C11 value was calculated as the ratio of the green fluorescence (indicates oxidized probe) to total fluorescence (green + red, indicates oxidized plus reduced probe).

Cell death analysis

Cells were plated in 96-well plates at a density of 2000 cells per well at 2 days before cold ischemia. After cold ischemia, the medium was replaced with fresh medium containing 30 nM SYTOX Green Dead Cell Stain. Then, the plates were immediately transferred to an IncuCyte Zoom imaging system (SARTORIUS)

enclosed in an incubator set to 37 °C and 5% CO_2. Three images per well were captured in the green and phase channels every 1 hour in a 48-h period, and the ratio of SYTOX Green-positive objects (dead cells) was quantified using Zoom image analysis software (Essen Bioscience).

Isolation of cardiomyocytes from transplanted hearts

The cardiomyocytes were isolated from transplanted hearts by using Langendorff perfusion system as previously described (4). Briefly, the Langendorff perfusion system was filled with 80 mL calcium-free modified Krebs-Henseleit protective solution (CZ0083, Leagene Biotech, China) gassed with 95% oxygen. Then, heat the perfusion system to 37°C and circulate the system for 5 min. Anesthesia the recipient rat with 4% isoflurane and 1 ml of 125 U/ml heparin was injected into its tail vein. After that, the rat underwent euthanasia through CO2 asphyxiation and the transplanted heart was excised, thoroughly rinsed to remove residual blood in ice-cold saline solution immediately, and then mounted on the Langendorff apparatus by fixing aorta on the connector. Subsequently, the heart was perfused with calcium-free modified Krebs-Henseleit protective solution for 5 min at velocity of 1 drop per second to further eliminate residual blood. Then, the heart was perfused with calcium-free modified Krebs-Henseleit protective solution supplemented with II-type collagenase (LS004176, Worthington, Germany) at a concentration of 250 U/ml. Following a 25-minute Langendorff perfusion of the heart, digestion was terminated by perfusing 5% fetal bovine serum (12484028, Gibco, United States). The heart was then excised, minced, and dispersed. After aspirating the suspension, filter the solution with the digested heart through a nylon mesh (200 μm) into a new 50ml tube. Centrifuge the filtered solution at 29 x g for 3 min. Discard the supernatant and add 6 mL Krebs-Henseleit protective solution including 12.5 μL CaCl2 (250 μM). Resuspend the pellet through smooth shaking movements. Centrifuge again at 29 x g for 2 min. Discard the supernatant and add 6 mL Krebs-Henseleit protective solution substituted with 25 μL CaCl2 (500 μM). Dissolve the cell pellet through gentle shaking movements and add 12 mL Krebs-Henseleit protective solution including 120 μL CaCl2 (1 mM). Centrifuge for a third time at 16 x g for 1 min. Again, remove the supernatant. Mix the cell pellet with the pre-warmed culture medium (AC-1001036, Applied Cell, China). The recalcified cardiomyocytes were then utilized for subsequent experiments.

Mitochondrial function assay

Prepare the culture plate from the Agilent Seahorse XFe24 kit (100777-004, Agilent, United States) and pre-hydrate the sensor cartridges in the culture plate according to the instructions overnight before use. Coat the Seahorse 24-well culture plate with extracellular matrix (354277, Corning, United States). Seed the isolated cardiomyocytes at a density of 5000 cells per well, evenly distributed by groups. Allow the cells to settle at room temperature for 2 hours. Once the cardiomyocytes adhere, wash the cells twice with XF assay medium (103075-100, Agilent, United States). Add 500 μL of XF assay medium for the next experimental steps. Following the instructions provided in the Agilent Seahorse XFe24 kit, sequentially add the XFp cell mito Stress test kit (103015-100, Agilent, United States) to the drug plate. This kit includes Oligomycin, FCCP, and Rotenone. After completing the drug additions, combine the drug plate with the culture plate and place them into the Seahorse XFe24 Analyzer (XFe24, Agilent, United States). After measurement, data were analyzed in Wave software (Agilent Technologies, Inc).

In vivo overexpression of *Sp1* in aged donor hearts

AAV9 vectors used for *Sp1* overexpression were products from Hanbio Biotechnology Co. Ltd (Shanghai, China). The AAV9 vectors carrying rat *Sp1* cDNA with a c-TnT promoter (AAV9-*Sp1*) were used in the

experimental group. The AAV9 vectors carrying the cTNT promoter (AAV9-*null*) were used in the control group. All AAV9 vectors were administered to the aged rats (1-year-old) through myocardial in situ injection for 150 μl at a dose of 1.6×10^{12} vg/ml. After 4 weeks, their hearts were harvested for transplantation. The overexpression of *Sp1* in aged donor hearts was verified by western blotting.

In vivo overexpression of *Cirbp* in aged donor hearts

AAV9 vectors used for *Cirbp* overexpression were products from Hanbio Biotechnology Co. Ltd (Shanghai, China). The AAV9 vectors carrying the cTNT promoter to drive the expression of *Cirbp* and firefly luciferase (AAV9-*Luc*-*Cirbp*) were used in the experimental group. The AAV9 vectors carrying the cTNT promoter to drive the expression of firefly luciferase (AAV9-*Luc*) were used in the control group. All AAV9 vectors were administered to the aged rats (1-year-old) through myocardial in situ injection for 200 μl at a dose of 1.2×10^{12} vg/ml. After 4 weeks, the successfully transfection of AAV9 into hearts in these aged rats was verified by in vivo luciferase imaging and then their hearts were harvested for transplantation. The overexpression of *Cirbp* in aged donor hearts was verified by western blotting.

In vivo overexpression of *Dhodh* in *Cirbp*-knockout young donor hearts

AAV9 vectors used for *Dhodh* overexpression were products from Hanbio Biotechnology Co. Ltd (Shanghai, China). The AAV9 vectors carrying the cTNT promoter to drive the expression of *Cirbp* and firefly luciferase (AAV9-*Luc*-*Dhodh*) were used in the experimental group. The AAV9 vectors carrying the cTNT promoter to drive the expression of firefly luciferase (AAV9-*Luc*) were used in the control group. All AAV9 vectors were administered to the *Cirbp*-knockout young rats (10-week-old) through myocardial in situ injection for 120 μl at a dose of 1.3×10^{12} vg/ml. After 4 weeks, the successfully transfection of AAV9 into hearts in these *Cirbp*-knockout young rats was verified by in vivo luciferase imaging and then their hearts were harvested for transplantation. The overexpression of *Dhodh* in *Cirbp*-knockout young donor hearts was verified by western blotting.

In vivo overexpression of *Sp1* in *Cirbp*-knockout aged donor hearts

AAV9 vectors used for *Sp1* overexpression were products from Hanbio Biotechnology Co. Ltd (Shanghai, China). The recombinant AAV9 vectors carrying rat *Sp1* cDNA with a c-TnT promoter (AAV9-*Sp1*) were used in the experimental group. The AAV9 vectors carrying the cTNT promoter (AAV9-null) were used in the control group. All AAV9 vectors were administered to the *Cirbp*-knockout aged rats (1-year-old) through myocardial in situ injection for 150 μl at a dose of 1.6×10^{12} vg/ml. After 4 weeks, their hearts were harvested for transplantation. The overexpression of *Sp1* in *Cirbp*-knockout aged donor hearts was verified by western blotting.

Echocardiography

M-mode echocardiography was performed on each rat after transplantation using a small animal echocardiography analysis system (Vevo770, VisualSonics). In brief, rats were anesthetized with isoflurane. The middle part of abdomen was moistened by the ultrasonic coupling agent as previously described (5). The heart rate (HR), left ventricular ejection fraction (EF), left ventricular end-diastolic diameter (LVEDD) and left ventricular end-systolic diameter (LVESD) were measured using a 30-MHz central frequency scan head after capturing the long axis section of the left ventricle. Percent fractional shortening (FS %) was calculated as (LVEDD-LVESD)/LVEDD.

RNA sequencing

Left ventricular tissues were collected at one day after transplantation. Total RNA was isolated using the Trizol Reagent (Invitrogen Life Technologies), after which the concentration, quality and integrity were determined using a NanoDrop spectrophotometer (Thermo Scientific). Three micrograms of RNA were used as input material for the RNA sample preparations. Sequencing libraries were generated using the TruSeq RNA Sample Preparation Kit (Illumina, San Diego, CA, USA). Briefly, mRNA was purified from total RNA using poly-T oligo-attached magnetic beads. Fragmentation was carried out using divalent cations under elevated temperature in an Illumina proprietary fragmentation buffer. First, strand cDNA was synthesized using random oligonucleotides and SuperScript II. Second, strand cDNA synthesis was subsequently performed using DNA Polymerase I and RNase H. Remaining overhangs were converted into blunt ends via exonuclease/polymerase activities and the enzymes were removed. After adenylation of the 3′ ends of the DNA fragments, Illumina PE adapter oligonucleotides were ligated to prepare for hybridization. To select cDNA fragments of the preferred 200 bp in length, the library fragments were purified using the AMPure XP system (Beckman Coulter, Beverly, CA, USA). DNA fragments with ligated adaptor molecules on both ends were selectively enriched using Illumina PCR Primer Cocktail in a 15 cycle PCR reaction. Products were purified (AMPure XP system) and quantified using the Agilent high sensitivity DNA assay on a Bioanalyzer 2100 system (Agilent). The sequencing library was then sequenced on an Illumina Novaseq 6000 by Shanghai Personal Biotechnology Cp. Ltd. Differential expression analysis of mapped RNA-seq data was performed using DESeq Genes with $P < 0.05$ and fold change <0.67 or >1.5 are identified as differentially expressed genes (DEGs). The pathway enrichment analyses of the differentially expressed genes were performed using Metascape (http://metascape.org) based on Kyoto Encyclopedia of Genes and Genomes (KEGG) pathway database. The data for RNA-sequencing are available in the National Center for Gene Expression Omnibus through series accession No. GSE227005.

TMT-based quantitative proteomics

Left ventricular tissues were harvested at one day after heart transplantation. The proteins from each sample were precipitated with acetone and redissolved in the triethylammonium bicarbonate buffer. Then, proteins were quantified by BCA protein assay kit (no. p0010, Beyotime) and about 100 μg of proteins were digested to peptides. The peptides were further labeled with TMT. Then the sample was fractionated using a C18 column on a Rigol L3000 HPLC system. For transition library construction, shotgun proteomics analyses were performed using an EASY-nLCTM 1200 UHPLC system (Thermo Fisher) coupled with a Q Exactive™ series mass spectrometer (Thermo Fisher). The resulting spectra from each run were searched separately by the search engines: Proteome Discoverer 2.2 (Thermo Fisher). In order to improve the quality of analysis results, the software PD 2.2 further filtered the retrieval results: Peptide Spectrum Matches (PSMs) with a credibility of more than 99% was identified PSMs. Differentially expressed proteins were selected according to following criteria: (1) $P < 0.05$; (2) fold change < 0.83 or fold change > 1.2. The proteins with significant differences between two groups were used for volcanic map analysis and Ingenuity pathway analysis. The proteins with significant differences are listed in Supplemental Table 2. The mass spectrometry proteomics data have been deposited to the ProteomeXchange Consortium via the iProX partner repository with the dataset identifier PXD044088.

Chromatin immunoprecipitation (ChIP)

Chromatin extracts were prepared from cardiac tissues of wild-type rats and aged rats. ChIP assays were performed using an EZ-Magna ChIP™ A/G Chromatin immunoprecipitation Kit (no. 17-10086, Millipore)

according to the manufacturer's instructions. Briefly, tissues were crumbled into pieces and fixed with formaldehyde, chromatin was isolated, and DNA was sheared into fragments by ultrasonic wave. The fragments were incubated with an SP1-specific antibody (4ug per ChIP, no. 21962-1-AP, proteintech) overnight at 4°C, after which the cross-linking was reversed, and proteins were digested with proteinase K. Then the purified DNA fragments were subjected to high-throughput sequencing based on a protocol from Myers Laboratory at Stanford University (http://www.hudsonalpha.org/myers-lab/protocols/). Library preparation and next-generation sequencing were performed by Novogene Biotechnology Corporation. Clean pair-end reads with high quality were mapped onto the rat genome with Bowtie2 algorithm (v.2.2.6). Only unique mapped reads were kept for subsequent analysis when reads mapped to multiple genomic positions. SP1-binding peak regions across the whole genome were extracted using MACS2 software (v.2.1.0). A signal density heatmap for enriched regions was calculated by deeptools (v.3.4.3) from .bam files. Potential binding sites of SP1 were determined by ChIP-sequencing, and then Real-time qPCR was performed using life Technology ABI 7500 System with primers flanking the GC box site at the *Cirbp* promoter region (5′-AATCTGAGGCGTCCGATTGG-3′ and 5′-TCACACACCCAAGCTTCCTAAC-3′) using a SYBR Green–based PCR kit (no. A25742, Thermo Fisher Scientific). The data for ChIP-sequencing are available in the National Center for Gene Expression Omnibus (GEO) through series accession No. GSE227005.

Biotin pulldown assay

The RNA pulldown analysis was conducted as previously described (6). The biotin labeled RNA probes were synthesized by CLOUD-SEQ Company (Shanghai, China). The labeled probe contained 3′-UTR *Dhodh* mRNA fragments and the negative control probe contained the reverse sequence of positive probes. The RNA-Dhodh probe: Biotin-GGATGGGACTTCTGGCACGTATGGTCACCTCCGATGATCTGCTCC AAT; and the negative control probe: Biotin-ATTGGAGCAGATCATCGGAGGTGACCATACGTGCCAG AAGTCCCATCC. The tissues harvested and washed with PBS twice and incubated in lysis buffer on ice for 10 min. The lysates were precleared by centrifugation. The probes were incubated with streptavidin-coated magnetic beads to generate probe-coated magnetic beads. The lysates were incubated with probe-coated beads and washed with the washing buffer. The RNA complexes bound to the beads were eluted and subjected to western blot.

UV cross-link RNP-IP assays

RIP was performed according to standard protocol (7). Cardiac tissues were exposed to 254nm UV (400 mJ/cm^2), and whole lysates were prepared for immunoprecipitation using anti-CIRBP (5 μg, no. 10209-2-AP, Proteintech) and anti-IgG antibodies. Briefly, 75 μl Protein-G-DYNA beads were incubated with 5 μg CIRBP antibody (overnight, 4°C) and washed three times with NT2 buffer (50 mM Tris–HCl (pH 7.5), 150 mM NaCl, 1 mM MgCl2, 0.05% NP-40), then added the lysates (100 mg) to Ab-beads and incubated for overnight at 4°C. The immunoprecipitated materials were washed five times with IP washing buffer (50 mM HEPES (pH 7.5), 300 mM KCl, 1 mM MgCl2, 0.05% NP40, 0.5 mM DTT). Then the materials were treated with NT2 buffer, Dnase I (2 U/ml), SDS (1%), 15 μl proteinase K and 5ul RNase inhibitor (30 min, 55°C). A magnetic rack is used to collect IP products. After purification of RNA with phenol chloroform, the transcripts present in the RNP complexes were analyzed by real-time qPCR. The primer sequences for RT-PCR were listed as below: *Dhodh* forward: 5′- GGCTAGCTGTTCGAGTCACC -3′; reverse: 5′-CCAAAGCCCAGCTTGTAGAG-3′.

RNC mRNAs-qPCR

Ribosome nascent-chain complex-bound (RNC) mRNAs-qPCR is based on the Polysome profiling method (8). In brief, the lysates were treated with cycloheximide to inhibit translation. Then the ribosome components were separated from the free mRNA and other cellular components by superspeed freezing centrifugation using a single concentration of sucrose solution buffer. RNC-mRNA can be obtained by extracting RNA in precipitation with phenol chloroform. At last, the transcripts were analyzed by real-time qPCR. The primer sequences for RT-PCR were listed as below: *Dhodh* forward: 5′-GGCTAGCTGTTCGAGTCACC -3′; reverse: 5′- CCAAAGCCCAGCTTGTAGAG-3′.

Cardiac arachidonic acid metabolites

All cardiac tissues were detected by ultra-performance liquid chromatography-mass spectrometry (UPLC-MS/MS, Agilent, USA). About 50 mg cardiac muscle samples were taken, weighed and added with grinding magnetic beads, followed by 500 μL of cold methanol, 10 μL of BHT methanol solution (4.8 g/100mL) and 50 uL of internal standard methanol solution (PGE2-D4 50 ng/mL, 5-HETE-D8 50 ng/mL). Then, the samples were homogenized using a tissue grinder (50 Hz, 90 s), and were extracted twice by adding cold methanol. After centrifugation (12000 rpm, 4°C, 10 min), the supernatant was dried, redissolved with 150μL methanol, diluted with pure water until the methanol content was less than 15%, and enriched in SPE column. After drying, the receiving solution was redissolved with 50 μL methanol, filtered by 0.22 μM filter membrane, and finally detected by UPLC-MS/MS. Data were collected and analyzed using Mass Hunter software (version B.08.00, Agilent, USA). Retention time and MRM ion pairs were qualitatively compared with the standards, and internal standard method was used for quantification.

Cardiac oxidative parameters assay

Cardiac oxidative parameters and iron content were detected using commercially available assay kits, according to the manufacturer's instructions (4-HNE, no. D751041-0096, Sangon; MDA, no. S0131, Beyotime; Iron content, no. BC4355, Solarbio)

Serum concentrations assay

Serum concentrations of cardiac enzymes were detected using commercially available ELISA kits, according to the manufacturer's instructions (cTNI, no. SEKR-0048, Solarbio; cTNT, no. SEKR-0047, Solarbio; CK, BC1140, Solarbio).

Histological analysis

The harvested cardiac tissues were fixed overnight in 4% paraformaldehyde buffer. Cardiac tissue was paraffin-embedded and sectioned at 4-5 μm. Then, sections were stained with H&E, PTAH and Prussian blue by using commercially available kits (no. DH0003, Leagene; no. DC0001, Leagene; G1424, Solarbio), according to the manufacturer's instructions.

Apoptosis analysis

Tissue sections were prepared after dewaxing, hydrate by graded ethanol and antigen retrieval using citrate. TUNEL staining was performed using an in-situ cell death detection kit (no. 11684795910, Roche) according to the manufacturer's protocol. The apoptotic index was measured by counting puncta of TUNEL signal in 100 randomly selected actinin-positive cells in multiple fields chosen randomly at 400× magnification. Images were captured by a laser-scanning confocal microscope (SP8, Leica).

Searching sequences matched with CIRBP binding motif in the 3' UTR region of *Dhodh* mRNA

A 51-nucleotide motif was identified as the CIRBP binding motif in a previous study (9). We scanned this motif in the 3'-UTR region of *Dhodh* mRNA by the FIMO software as previously described (10). The results showed that there are four matched sequences (Supplemental Table 3)

Immunofluorescence staining in cardiac tissues

The immunofluorescence was performed following a standard protocol as previously described (11). Briefly, after dewaxing, hydration and antigen retrieval, paraffin-embedded sections were blocked with 5% serum from goat for 2 hours and incubated with primary antibodies overnight at 4°C. Some sections were incubated with IgG to test the specificity of antibodies at same time. Then, sections were stained with fluorochrome-conjugated secondary antibodies for 60 min. Images were captured by a laser-scanning confocal microscope (SP8, Leica). The fluorescence intensity and area were measured by Image J software (version 1.52a). The MPO-positive number was determined by counting puncta of MPO signals in 3 randomly selected views. The primary antibodies were listed in supplementary Supplemental Table 4.

Immunofluorescence staining in cardiomyocytes

The immunofluorescence was performed following a standard protocol as previously described (7). Briefly, cardiomyocytes were fixed in 4% paraformaldehyde. Thereafter, cardiomyocytes were blocked with 5% serum from goat for 2 hours and incubated with primary antibodies overnight at 4°C. Some cardiomyocytes were incubated with IgG to test the specificity of antibodies at same time. Then, cardiomyocytes were stained with fluorochrome-conjugated secondary antibodies for 60 min. Images were captured by a laser-scanning confocal microscope (SP8, Leica). The fluorescence intensity and area were measured by Image J software (version 1.52a). The primary antibodies were listed in Supplemental Table 4.

RT-PCR analysis

Total RNA was extracted using the Trizol reagent (no. 15596018, Thermo Fisher Scientific). Reverse transcription was performed using a Primescript RT reagent kit (no. RR047Q, Takara) and RT-PCR was performed using life Technology ABI 7500 System based on SYBR-Green PCR kit (no. A25742, Thermo Fisher Scientific). The △△Ct method was used for the calculation of relative changes in gene expression. The primer sequences for RT-PCR were listed as below: *Cirbp* forward: 5′-GGTCTCAGCTTCGACACCAA -3′; reverse: 5′- TCCCATCCACAGACTTCCCA -3′. *Dhodh* forward: 5′-CCGAGGACCAAGCTGTCATT-3′ and reverse: 5′-GTAAGGGCGTACATCTCCCG-3′. *Gapdh* forward: 5′- CTCTCTGCTCCTCCCTGTTC -3′; reverse: 5′- GCCAAATCCGTTCACACCG -3′.

Cytosolic and nuclear proteins extraction

Cytoplasmic and nuclear proteins were extracted from cardiac samples using commercially available assay kits according to the manufacturer's instructions (Minute^{TM}, no. NT-032, Invent).

Western blot analysis

Western blot analysis was performed following a standard protocol as previously described (7). Briefly, total proteins of tissues and cells were extracted using RIPA buffer with protease inhibitors (Roche). For immunoblot analysis, protein samples were resolved by SDS-PAGE and transferred onto nitrocellulose membranes by an iBlot 2 dry blotting system (Thermo Fisher Scientific). Membranes were blocked in 5% non-fat dry milk in TBST (100 mmol/L Tris, pH 7.5, 0.9% NaCl, 0.1% Tween-20) for 1 hour and incubated

with primary antibodies overnight at 4°C. Membranes were washed 3 times with TBST and incubated for 1 hour with horseradish peroxidase-labeled secondary antibody at room temperature. After rinsing, enhanced chemiluminescent substrate (no. WBKLS0500, Millipore) was used to develop the membranes. Amersham Imager 600 (GE) was used to capture and analyze the band intensities. The primary antibodies were listed in Supplemental Table 5.

Transmission electron microscope (TEM)

Samples of myocardium (1 mm×1 mm×2 mm) were quickly removed from the left ventricle and immediately fixed with 2.5% glutaraldehyde overnight at 4°C for 48 hours. The fixed tissues were then dehydrated with ethanol and embedded in ultra-thin sections. After the sections were counterstained with uranyl acetate and lead citrate, they were observed under the transmission electron microscope (HT7500, Hitachi, Japan). Five random fields from each sample were screened, and the abnormal mitochondria with mitochondrial shrinkage, mitochondrial exacerbated crista loss and mitochondrial membrane rupture were preserved.

CoQ and $CoQH_2$ analysis

The frozen tissue was cut on dry ice and weighted (20 ± 2 mg). For cell experiments, a minimum of 8×10^6 cells need to be collected. The samples were spiked with 100 μL 96% ethanol containing BHT and 100 ng/mL CoQ10-d9 as internal standard, and homogenized in the frozen state by using a Tissuelyser with zirconia beads at 40 Hz for 4 min. The homogenates were centrifuged at 15000 *g* and 4 °C for 15 min, and the supernatant was collected and evaporated to dryness under nitrogen. The dry residue was reconstituted in 50 μL ethanol prior to performing UHPLC-MS/MS analysis. The quality control (QC) sample was obtained by isometrically pooling all the prepared samples. The samples of calibration curves were prepared for ubiquinone and ubiquinol, respectively. The UHPLC-MS/MS analysis was performed on an Agilent 1290 Infinity II UHPLC system coupled to a 6470A Triple Quadrupole mass spectrometry (Santa Clara, CA, United States). Samples was injected onto a Thermo Fisher Acclaim C30 column (100 mm × 2.1 mm, 3 μm) at a flow rate of 0.3 mL/min. The mobile phases consisted of (A) 60% acetonitrile and (B) isopropanol/acetonitrile (9:1, v/v), both with 10mM ammonium formate. The chromatographic separation was conducted by a gradient elution program as follows: 10% B at 0-0.5 min, 70% B at 1.5 min, 90% B at 8 min, 100% B at 8.5 min and held to 9.5 min, 10% B at 9.6 min and held to 11min. The eluted analysts were ionized in an electro spray ionization source in positive mode (ESI+). The temperatures of source drying gas and sheath gas were 300 °C and 350 °C. The flow rates of source drying gas and sheath gas were 5 and 11 L/min, respectively. The pressure of nebulizer was 45 psi, and capillary voltage was 4000 V. Multiple reaction monitoring mode was applied to determine ubiquinone (880.7->197 for quantification and 880.7->95 for qualification) and ubiquinol (882.7->197 for quantification and 882.7->95 for qualification). The Agilent MassHunter software (version B.08.00) was used to control instruments and acquire data. The raw data were processed by MassHunter Workstation Software (version B.08.00, Agilent) by using the default parameters and assisting manual inspection to ensure the qualitative and quantitative accuracies of each compound. The peak areas of each compound in all samples were integrated. Calibration curves of 11-point were constructed by plotting the peak area ratio of each compound to internal standard against concentration of each compound. The molar amount of ubiquinone (oxidized CoQ10) in the ubiquinol (reduced CoQ10) sample was firstly calculated using the calibration curve of ubiquinone, and then the true molar concentration of ubiquinol, after subtracting the ubiquinone molar concentration from the nominal value of ubiquinol sample, was used to plot the calibration curves of ubiquinol. The ubiquinone (CoQ) and ubiquinol ($CoQH_2$) contents (nmol/g wet tissue) of raw sample were quantified in Excel software.

Supplementary references

1. Ono K. Lindsey ES. Improved technique of heart transplantation in rats. *J Thorac Cardiovasc Surg.* 1969;57(2):225-229.
2. Guo Gr, et al. A modified method for isolation of human cardiomyocytes to model cardiac diseases. *J Transl Med.* 2018;16(1):288.
3. Shi B, et al. Targeting gut microbiota-derived kynurenine to predict and protect the remodeling of the pressure-overloaded young heart. *Sci Adv.* 2023;9(28):eadg7417.
4. Nippert F, et al. Isolation and Cultivation of Adult Rat Cardiomyocytes. *J Vis Exp.* 2017;128:56634.
5. Liu Y, et al. Effect of chronic left ventricular unloading on myocardial remodeling: Multimodal assessment of two heterotopic heart transplantation techniques. *J Heart Lung Transplant.* 2015;34(4):594-603.
6. Min X, et al. A circular intronic RNA ciPVT1 delays endothelial cell senescence by regulating the miR-24-3p/CDK4/pRb axis. *Aged Cell.* 2022;21(1):e13529.
7. Liu Y, et al. Chronic hypoxia-induced *Cirbp* hypermethylation attenuates hypothermic cardioprotection via down-regulation of ubiquinone biosynthesis. *Sci Transl Med.* 2019;11(489):eaat8406.
8. Wang T, et al. Translating mRNAs strongly correlate to proteins in a multivariate manner and their translation ratios are phenotype specific. *Nucleic Acids Res.* 2013;41(9):4743-4754.
9. Yang R, et al. Functional significance for a heterogenous ribonucleoprotein A18 signature RNA motif in the 3'-untranslated region of ataxia telangiectasia mutated and Rad3-related (ATR) transcript. *J Biol Chem.* 2010;285(12): 8887-8893.
10. Grant CE, et al. FIMO: scanning for occurrences of a given motif. *Bioinformatics.* 2011; 27(7): 1017-1018.
11. Liu Y, et al. Suppression of Myocardial Hypoxia-Inducible Factor-1α Compromises Metabolic Adaptation and Impairs Cardiac Function in Patients With Cyanotic Congenital Heart Disease During Puberty. *Circulation.* 2021;2:2254-2272.

Supplemental Figures

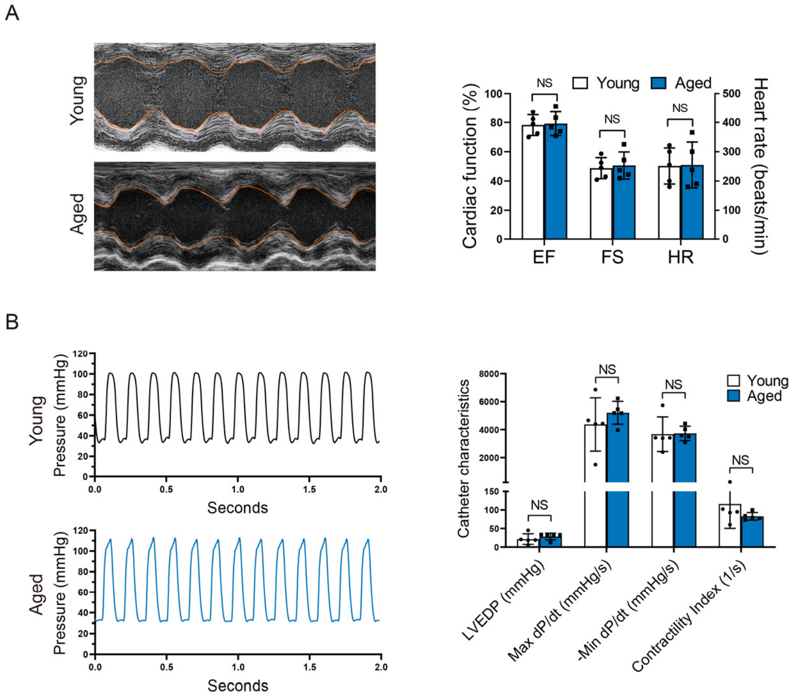

Supplemental Figure 1. Evaluation of cardiac functions in young and aged donor heart prior to transplantation (**A**) Cardiac function of donor hearts measured by echocardiography prior to transplantation. EF, ejection fraction; FS, fractional shortening. (**B**) Representative left ventricular pressure (LVP) traces and cardiac function parameters of donor hearts determined via cardiac catheterization prior to transplantation. LVEDP, left ventricular end-diastolic pressure. Quantitative data are shown as the mean ± standard deviation with individual values presented as a dot plot. Statistical significance was analyzed by Student's *t* test (NS, not significant).

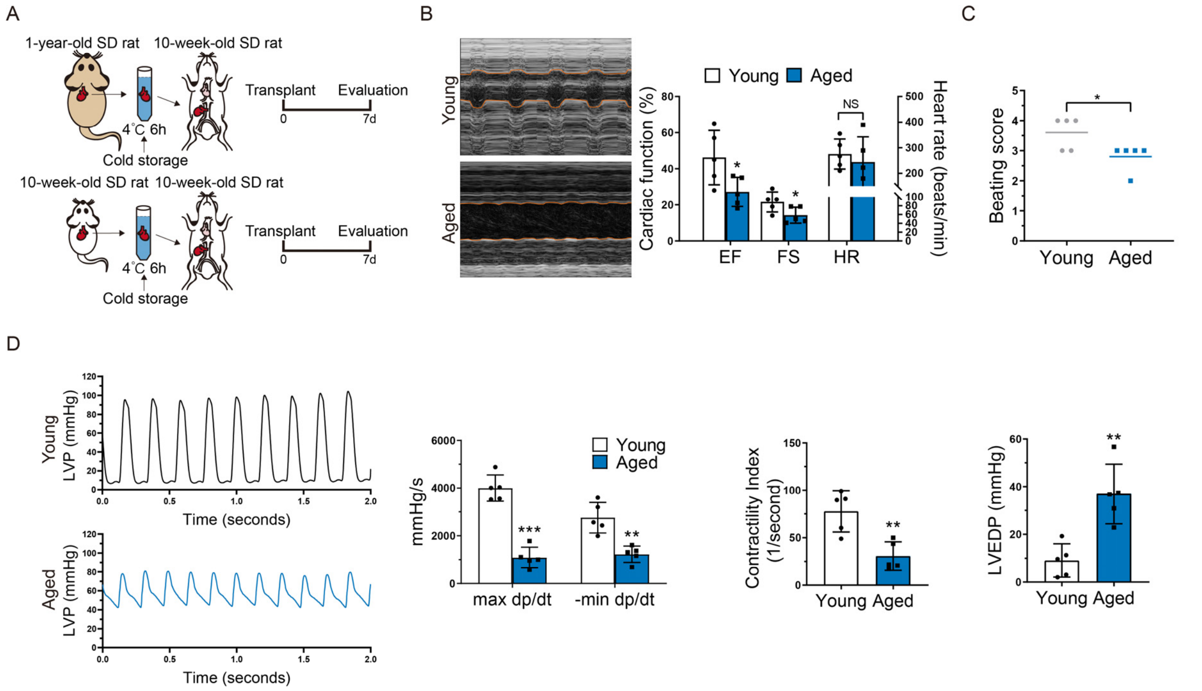

Supplemental Figure 2. Aged donor hearts showed decreased cardiac functions at 7 days after transplantation. (A) Experimental design. Donor hearts were harvested from young rats (10-week-old) and aged rats (1-year-old) and then transplanted to young rats (10-week-old) (n=5 in each group). Hypothermia was used to protect the donor hearts during cold storage. The beating score and donor heart function were measured at 7 days after transplantation. SD, Sprague-Dawley. (**B**) Cardiac function of donor hearts measured by echocardiography at 7 days after transplantation. EF, ejection fraction; FS, fractional shortening; SV, stoke volume; CO, cardiac output; HR, heart rate. **(C)** Beating score of donor hearts at 7 days after transplantation. (**D**) Representative left ventricular pressure (LVP) traces and cardiac function parameters of donor hearts determined via cardiac catheterization at 7 days after transplantation. LVEDP, left ventricular end-diastolic pressure. Quantitative data are shown as the mean ± standard deviation with individual values presented as a dot plot. Statistical significance of beating score was determined with Mann-Whitney test. Statistical significance of the other parameters was determined with Student's t test (**P* < 0.05; ***P* < 0.01; ****P* < 0.001; NS, not significant).

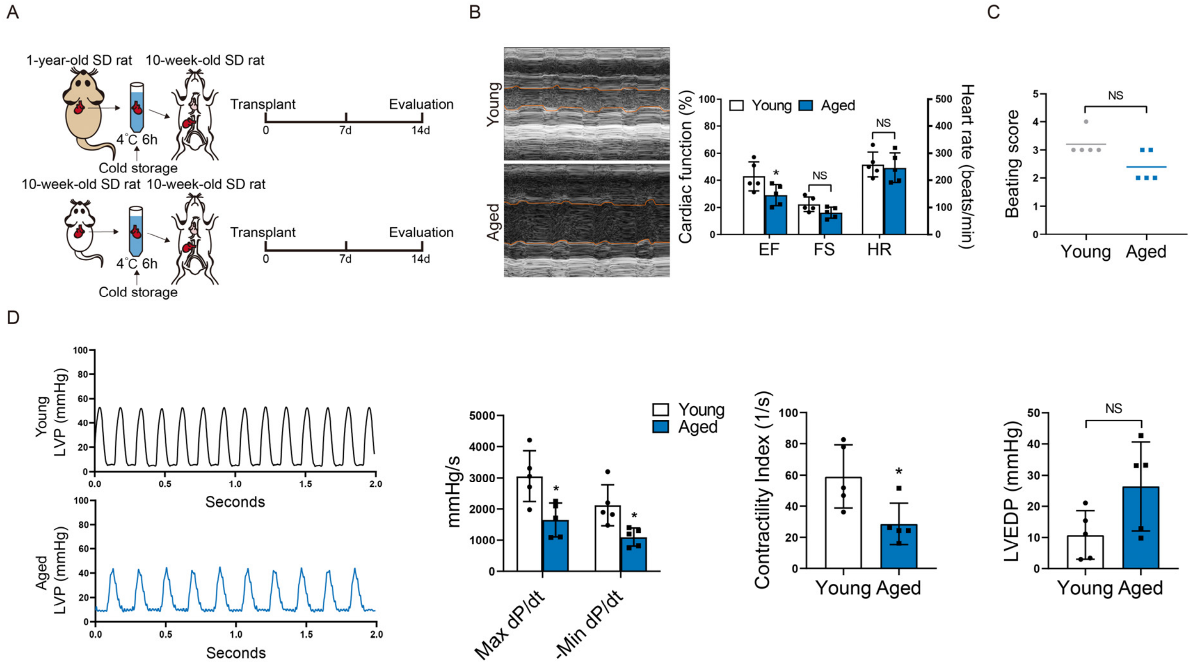

Supplemental Figure 3. Aged donor hearts showed decreased cardiac functions at 14 days after transplantation. (**A**) Experimental design. Donor hearts were harvested from young rats (10-week-old) and aged rats (1-year-old) and then transplanted to young rats (10-week-old) (n=5 in each group). Hypothermia was used to protect the donor hearts during cold storage. The beating score and donor heart function were measured at 14 days after transplantation. SD, Sprague-Dawley. (**B**) Cardiac function of donor hearts measured by echocardiography at 14 days after transplantation. EF, ejection fraction; FS, fractional shortening; HR, heart rate. **(C)** Beating score of donor hearts at 14 days after transplantation. (**D**) Representative left ventricular pressure (LVP) traces and cardiac function parameters of donor hearts determined via cardiac catheterization at 14 days after transplantation. LVEDP, left ventricular end-diastolic pressure. Quantitative data are shown as the mean ± standard deviation with individual values presented as a dot plot. Statistical significance of beating score was determined with Mann-Whitney test. Statistical significance of the other parameters was determined with Student's t test (**P* < 0.05; NS, not significant).

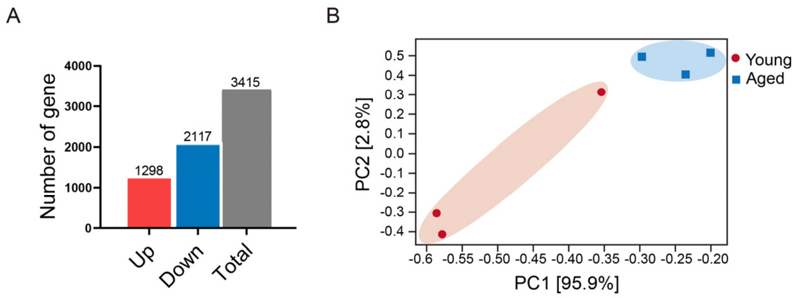

Supplemental Figure 4. Transcriptomic analysis of the young and aged donor heart after transplantation (**A**) The total numbers of genes whose expression were significantly increased or decreased between two groups. (**B**) PCA plots showing the separation of clusters between two groups (n=3 in each group).

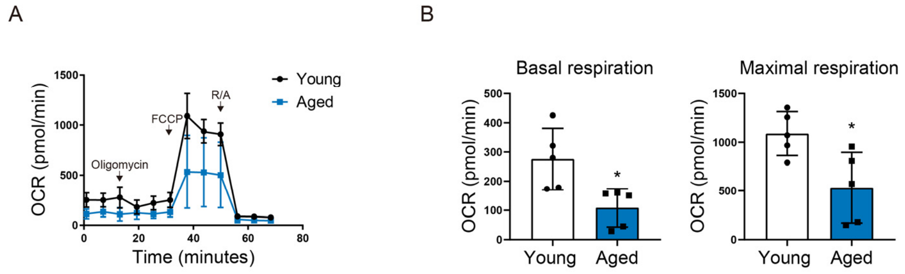

Supplemental Figure 5. Aged donor hearts showed impaired mitochondrial functions at 1 day after transplantation. (**A**) Oxygen consumption rate (OCR) of cardiomyocytes isolated from young or aged donor hearts at 1 day after transplantation. The traces were recorded by XF24 metabolic analyzer and the mitochondrial effectors were injected at time points indicated by downward arrows. (**B**) Basal respiration and maximal respiration of cardiomyocytes isolated from two groups. Statistical significance was analyzed by Student's *t* test (*$P < 0.05$).

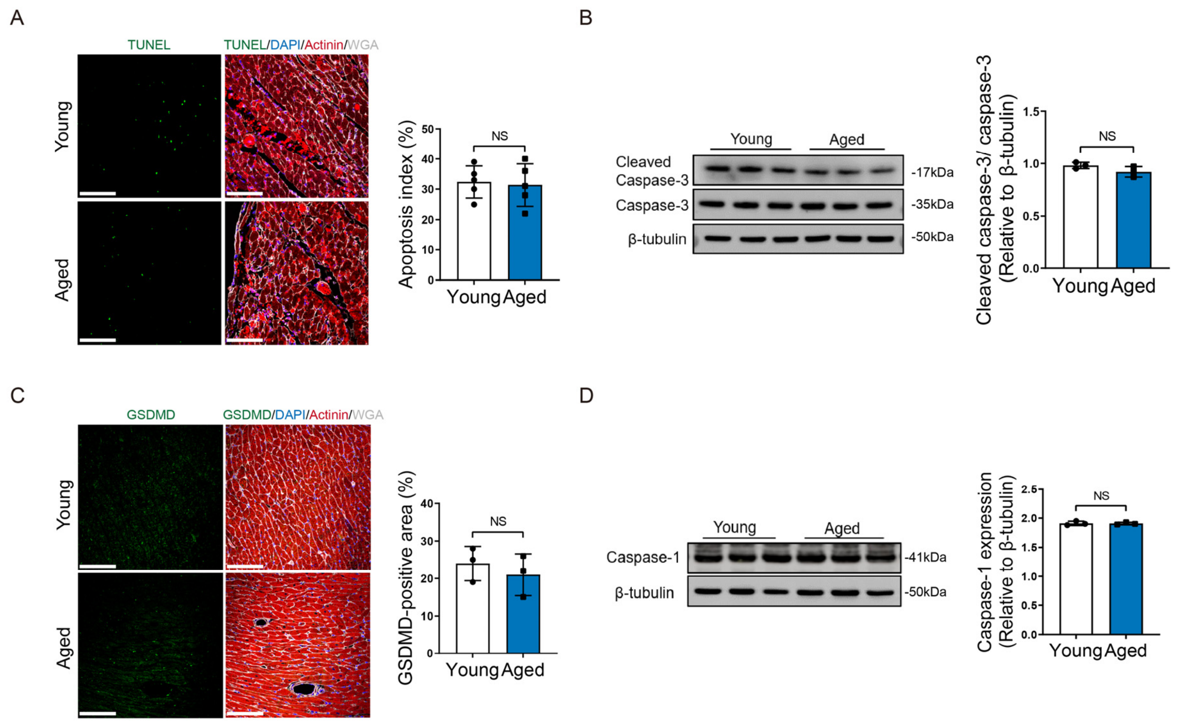

Supplemental Figure 6. The levels of apoptosis and pyroptosis are comparable between the young and aged donor heart after transplantation. (**A**) TUNEL staining in young and aged donor hearts after transplantation. Scale bars, 100 μm. The apoptotic index was measured by counting puncta of TUNEL signal in 100 randomly selected actinin-positive cells. (**B**) Western blot analysis of cleaved caspase-3 and caspase-3 in young and aged donor hearts after transplantation. The graph shows the ratio of cleaved caspase-3 to caspase-3. (**C**) Immunofluorescence staining of GSDMD in young and aged donor hearts after transplantation. Scale bars, 100 μm. (**D**) Western blot analysis of caspase-1 in young and aged donor hearts after transplantation. Quantitative data are shown as the mean ± standard deviation with individual values presented as a dot plot. Statistical significance was analyzed by Student's *t* test (NS, not significant).

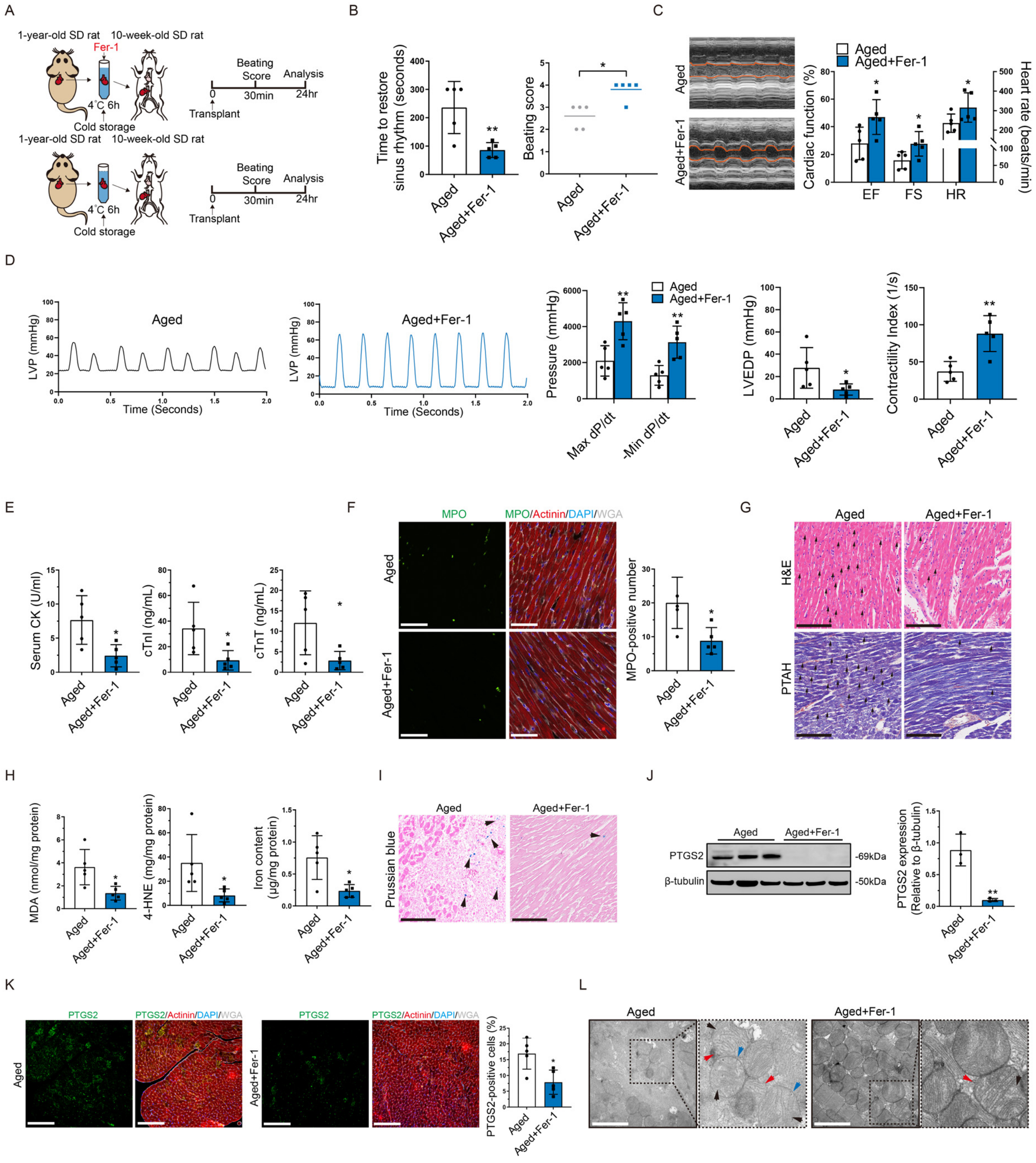

Supplemental Figure 7. Cardioplegic solution supplemented with another ferroptosis inhibitor also could improve cardioprotection of aged donor heart during cold storage in transplantation. (**A**) Experimental design. Donor hearts were harvested from aged rats (1-year-old). UW solution was used to induce cardiac arrest in control group, and the experimental group used UW solution supplemented with another ferroptosis inhibitor, ferrostatin-1 (Fer-1) (n=5 in each group). After 6-hours cold ischemia, donor hearts were transplanted to young rats (10-week-old). Hypothermia was used to protect the donor heart during cold storage. The beating score was evaluated at 30 mins after transplantation. The donor heart functions and myocardial injury indicators were measured at 1 day after transplantation. SD, Sprague-Dawley. (**B**) Assessment of cardiac resuscitation after transplantation. (**C**) Cardiac function of donor hearts measured by echocardiography after transplantation. (**D**) Representative left ventricular pressure (LVP) traces and cardiac function parameters of donor hearts determined via cardiac catheterization after

transplantation. LVEDP, left ventricular end-diastolic pressure. (**E**) Serum cardiac enzymes in recipients after transplantation. (**F**) MPO staining of donor hearts after transplantation. Scale bars, 50 µm. (**G**) H&E and PTAH staining of donor hearts after transplantation. The arrows indicate myocardial contraction band necrosis. Scale bars, 100µm in H&E and 50µm in PTAH. (**H**) Peroxidation parameters and iron overload in donor hearts after transplantation. (**I**) Prussian blue staining of donor hearts after transplantation. The arrows indicate iron deposition in myocardium. Scale bars, 50 µm. (**J**) Western blotting and quantification of PTGS2 in donor hearts after transplantation. (**K**) Immunofluorescence staining of PTGS2 in donor hearts after transplantation. Scale bars, 100 µm. (**L**) Transmission electron microscopy images of donor hearts after transplantation. Blue arrows indicate mitochondrial shrinkage; Black arrows indicate the mitochondrial crista loss; Red arrows indicate mitochondrial membrane rupture. Scale bars, 1 µm. Quantitative data are shown as the mean ± standard deviation with individual values presented as a dot plot. Statistical significance of beating score was determined with Mann-Whitney test. Statistical significance of the other parameters was determined with Student's *t* test (*$P < 0.05$; **$P < 0.01$; ***$P < 0.001$; NS, not significant).

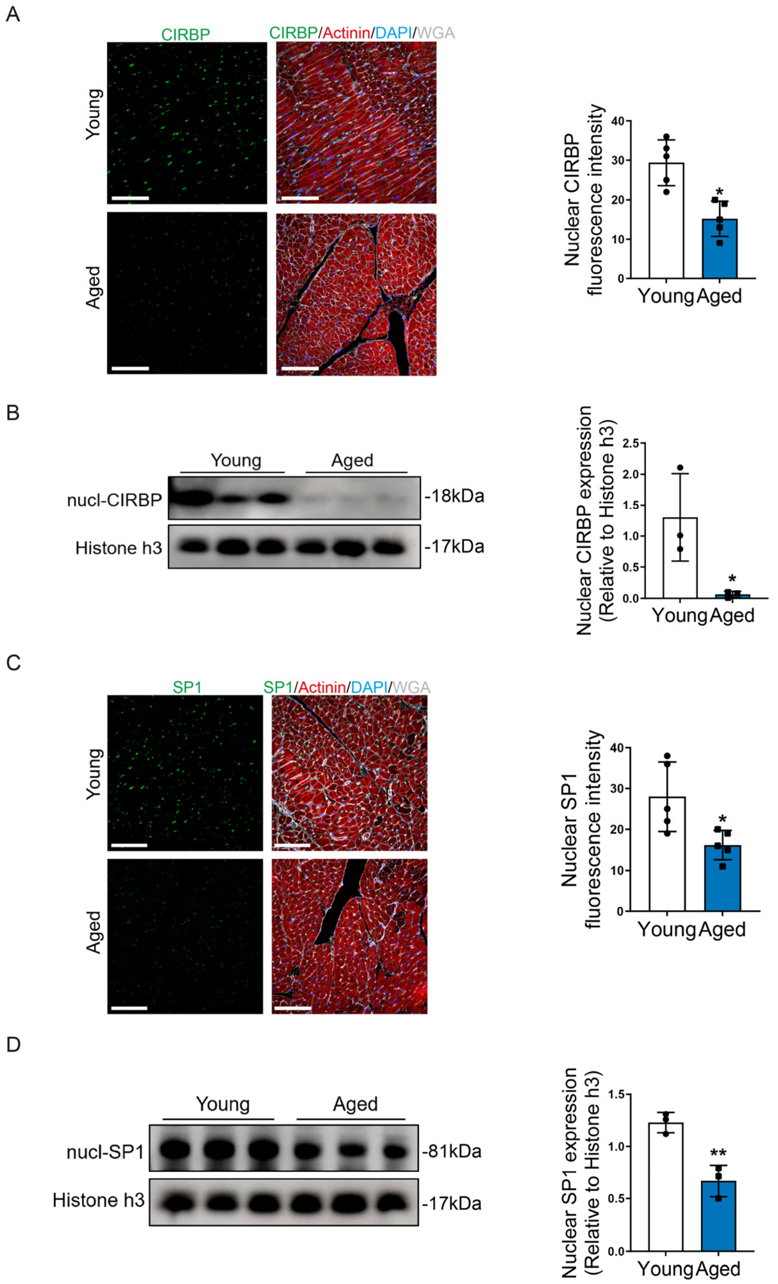

Supplemental Figure 8. The expression of CIRBP and SP1 in young and aged human donor hearts. (**A**) Immunofluorescence staining of CIRBP in young and aged human donor hearts before transplantation. Scale bars, 100 μm. (**B**) Western blotting and quantification of nuclear CIRBP in young and aged human donor hearts before transplantation. (**C**) Immunofluorescence staining of SP1 in young and aged human donor hearts before transplantation. Scale bars, 100 μm. (**D**) Western blotting and quantification of nuclear SP1 in young and aged human donor hearts before transplantation. Quantitative data are shown as the mean ± standard deviation with individual values presented as a dot plot. Statistical significance was analyzed by Student's *t* test (*$P < 0.05$; **$P < 0.01$).

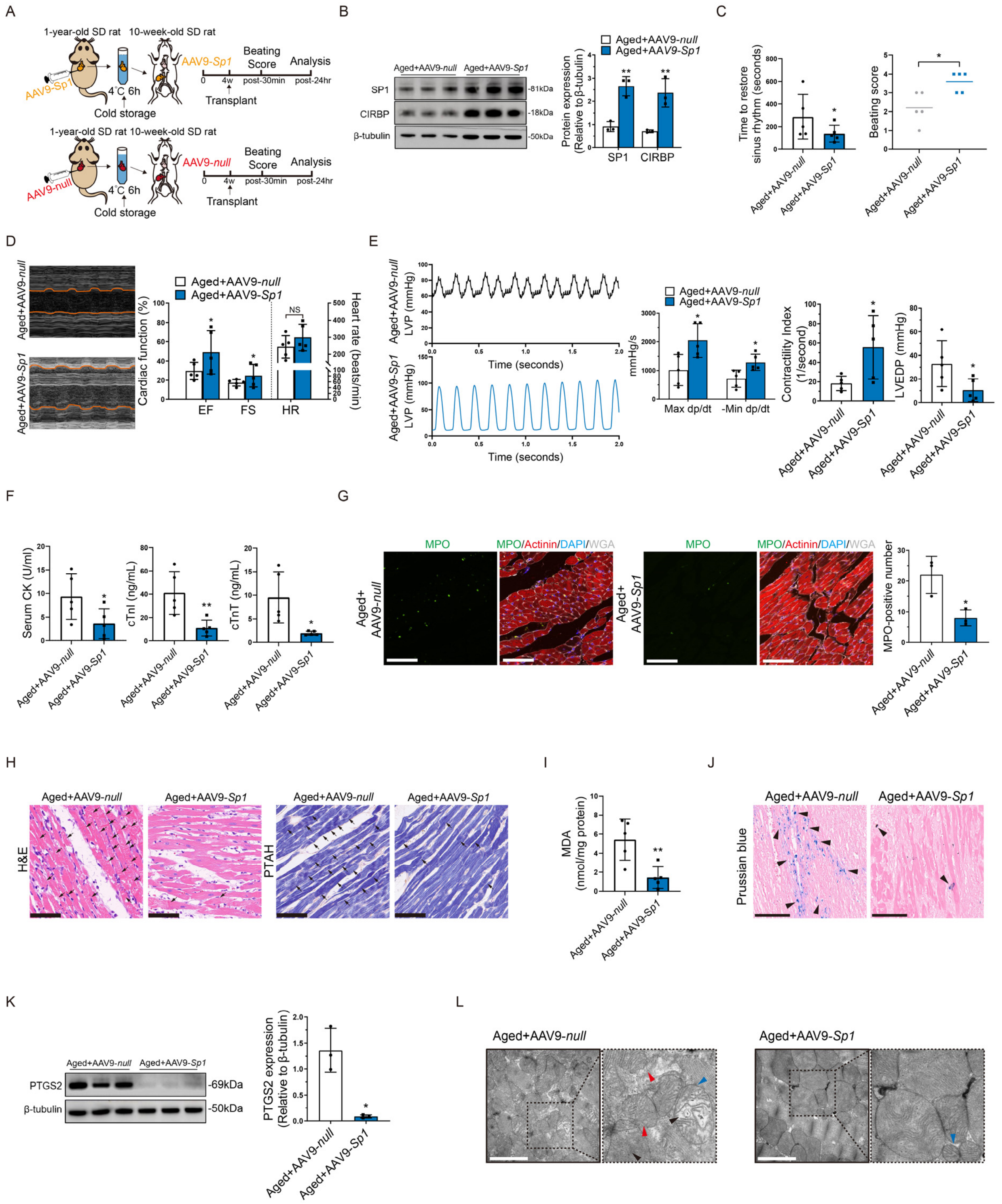

Supplemental Figure 9. Overexpression of *Sp1* enhances hypothermic cardioprotection in aged donor hearts during cold storage in transplantation. (**A**) Experimental design. Aged rats (1-year-old) were injected with AAV9 vectors carrying *Sp1* cDNA (AAV9-*Sp1*) or control vehicles (AAV9-*null*) (n=5 in each group). After 4 weeks, the donor hearts were harvested from two groups and transplanted to the young rats (10-week-old). Hypothermia was used to protect the donor heart during cold storage. The beating score was evaluated at 30 mins after transplantation. The donor heart functions and myocardial injury indicators were measured at 1 day after transplantation. SD, Sprague-Dawley. (**B**) Western blotting and quantification of SP1 and CIRBP in *Sp1*-overexpressing aged donor hearts. (**C**)Assessment of cardiac resuscitation after

transplantation (**D**) Cardiac function of donor hearts measured by echocardiography after transplantation. (**E**) Representative left ventricular pressure (LVP) traces and cardiac function parameters of donor hearts determined via cardiac catheterization after transplantation. LVEDP, left ventricular end-diastolic pressure. (**F**) Serum cardiac enzymes in recipients after transplantation. (**G**) MPO staining of donor hearts after transplantation. Scale bars, 50 µm. (**H**) H&E and PTAH staining of donor hearts after transplantation. The arrows indicate myocardial contraction band necrosis. Scale bars, 100µm in H&E and 50µm in PTAH. (**I**) Lipid peroxidation in donor hearts after transplantation. (**J**) Prussian blue staining of donor hearts after transplantation. The arrows indicate iron deposition in myocardium. Scale bars, 50 µm. (**K**) Western blotting and quantification of PTGS2 in donor hearts after transplantation. (**L**) Transmission electron microscopy images of donor hearts after transplantation. Blue arrows indicate mitochondrial shrinkage; Black arrows indicate the mitochondrial crista loss; Red arrows indicate mitochondrial membrane rupture. Scale bars, 1 µm. The data shown are the mean ± standard deviation with individual values presented as a dot plot. Statistical significance of beating score was determined with Mann-Whitney test. Statistical significance of the other parameters was determined with Student's *t* test (*$P < 0.05$; **$P < 0.01$; ***$P < 0.001$; NS, not significant).

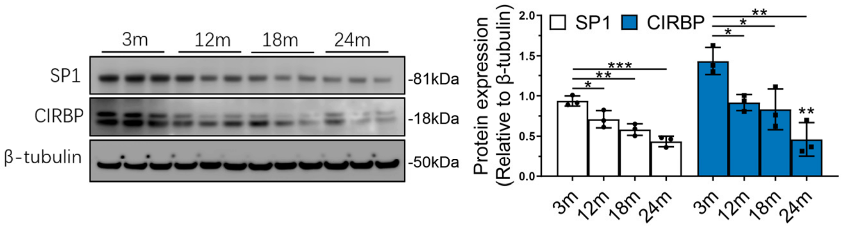

Supplemental Figure 10. Dynamic changes in expressions of SP1 and CIRBP in donor hearts from rats at different ages. Quantitative data are shown as the mean ± standard deviation with individual values presented as a dot plot. Statistical significance was analyzed by one-way ANOVA followed by Tukey–Kramer multiple-comparisons test (**P* < 0.05; ***P* < 0.01; ****P* < 0.001).

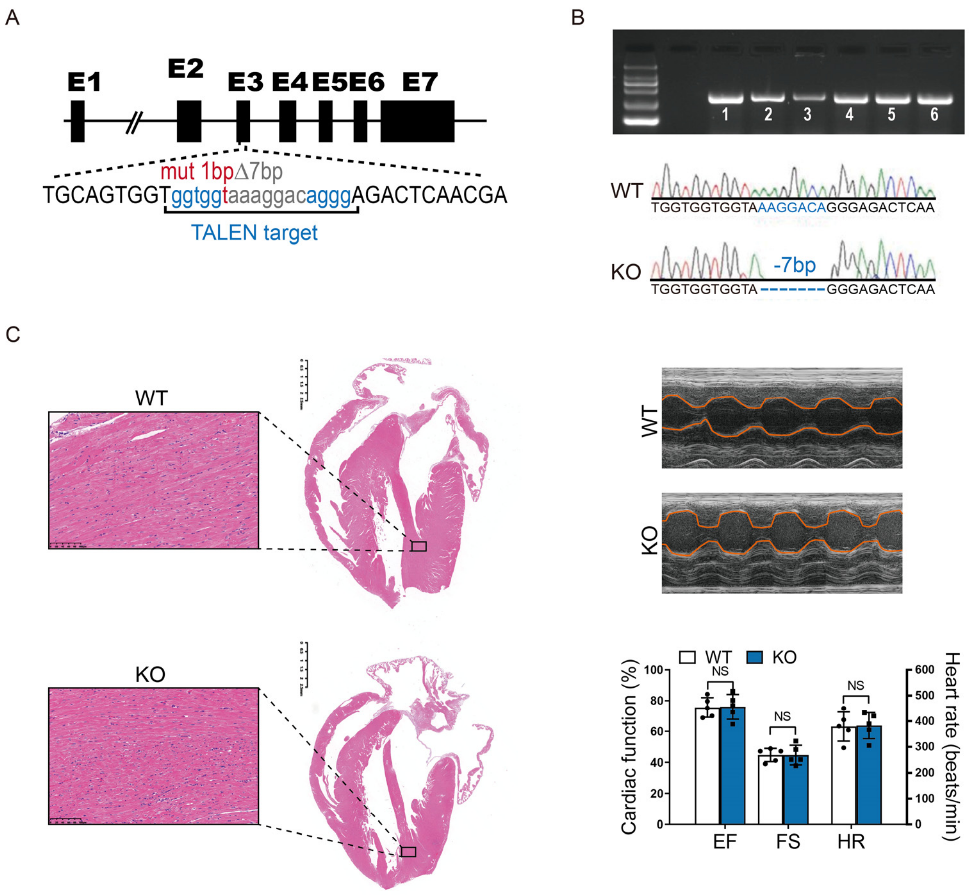

Supplemental Figure 11. Baseline assessment of cardiac histology and heart function in *Cirbp*-knockout rats. (**A**) A 17 bp sequence in the exon 3 of *Cirbp* was chosen as TALEN-target for deletion. (**B**) Genotyping of *Cirbp*-knockout rats. Upper: Representative gel electrophoresis image of PCR products of *Cirbp*-knockout rats. Lower: Confirmation of successful gene deletion by direct DNA sequencing of PCR products from the upper image. (**C**) H&E staining in cardiac tissues (original magnification, ×100). scale bar, 100 µm. (**D**) Echocardiographic evaluation of cardiac function (n = 3 in each group). The data shown are the mean ± standard deviation with individual values presented as a dot plot. Statistical significance was analyzed by Student's *t* test (NS, not significant).

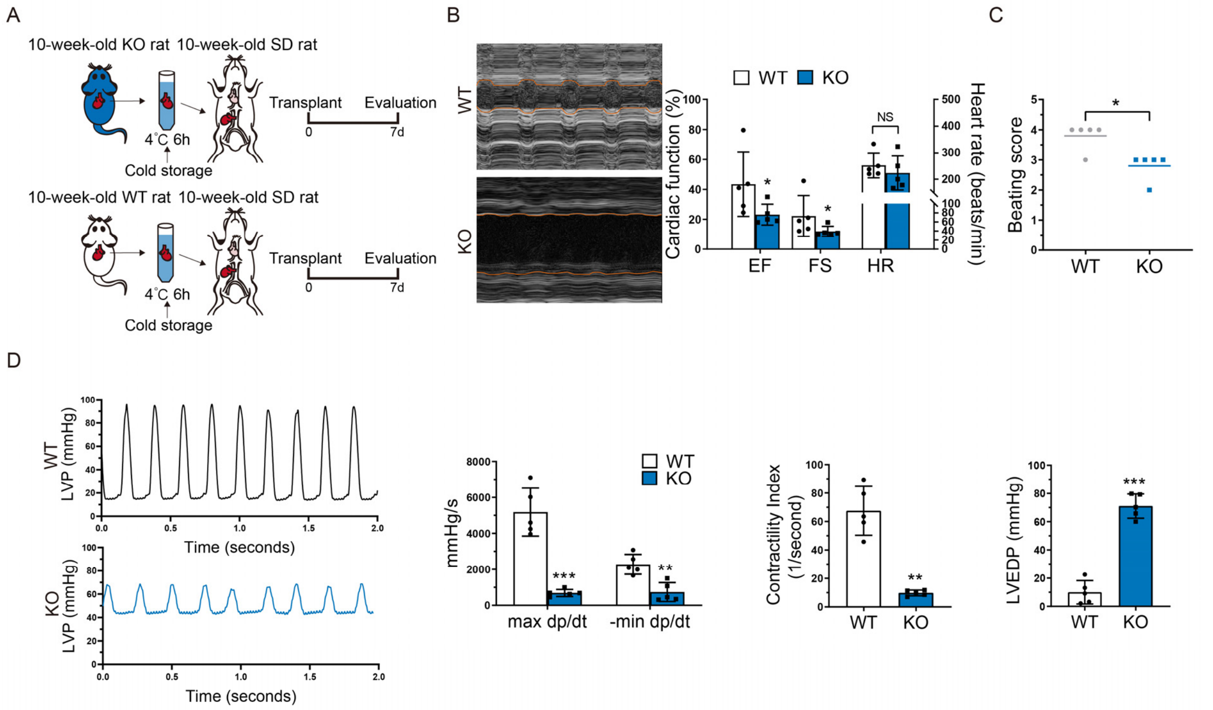

Supplemental Figure 12. ***Cirbp*-knockout young donor heart showed decreased cardiac functions at 7 days after transplantation.** (**A**) Experimental design. Young donor hearts were harvested from wild-type (10-week-old) (WT) rats and *Cirbp*-knockout (10-week-old) (KO) rats, and were transplanted to wild-type young rats respectively (10-week-old) (n=5 in each group). Hypothermia was used to protect the donor heart during cold storage. The beating score and donor heart function were measured at 7 days after transplantation. SD, Sprague-Dawley. (**B**) Cardiac function of donor hearts measured by echocardiography at 7 days after transplantation. EF, ejection fraction; FS, fractional shortening; SV, stoke volume; CO, cardiac output; HR, heart rate. **(C)** Beating score of donor hearts at 7 days after transplantation. (**D**) Representative left ventricular pressure (LVP) traces and cardiac function parameters of donor hearts determined via cardiac catheterization at 7 days after transplantation. LVEDP, left ventricular end-diastolic pressure. Quantitative data are shown as the mean ± standard deviation with individual values presented as a dot plot. Statistical significance of beating score was determined with Mann-Whitney test. Statistical significance of the other parameters was determined with Student's *t* test (*$P < 0.05$; **$P < 0.01$; ***$P < 0.001$; NS, not significant).

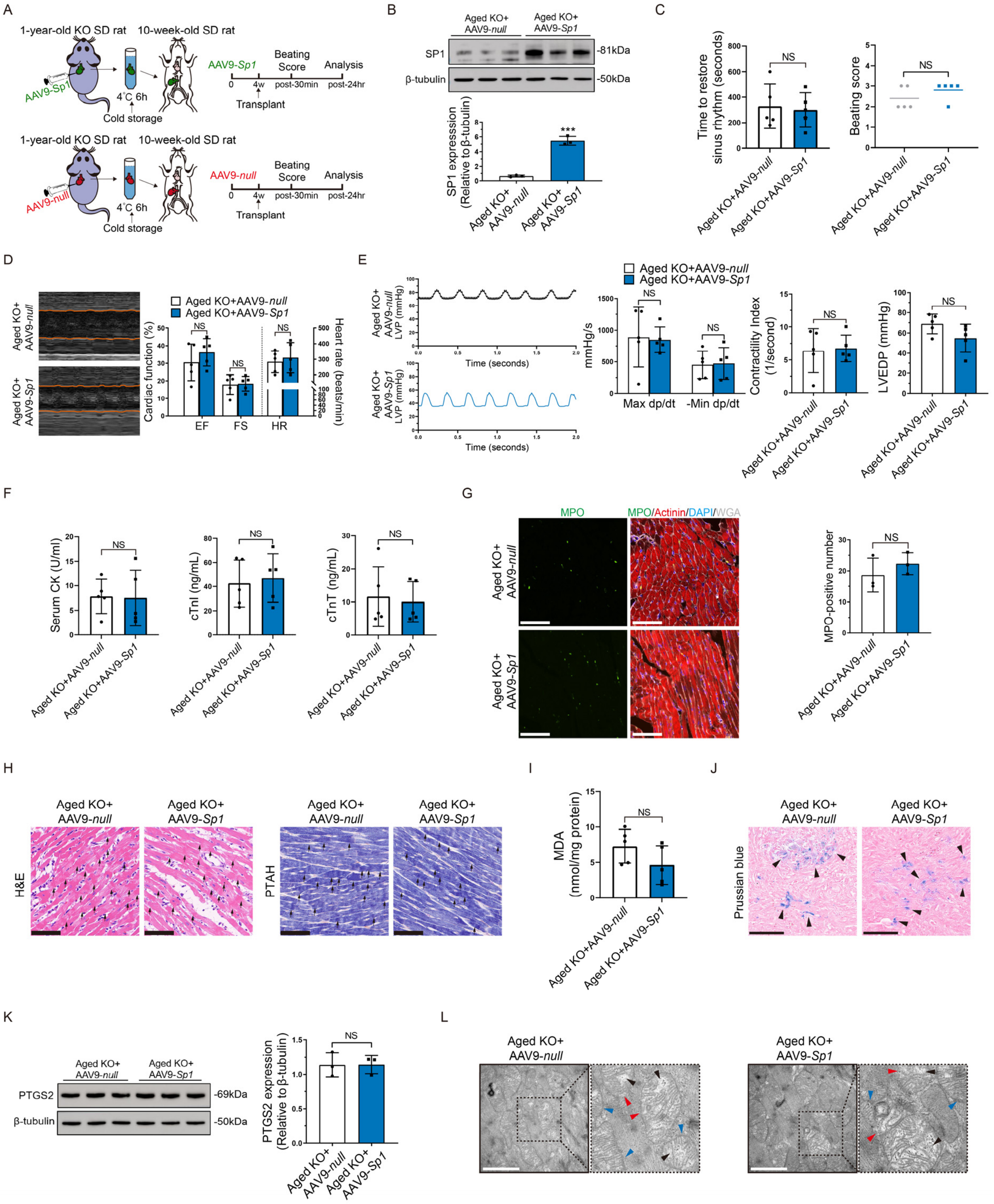

Supplemental Figure 13. Overexpression of *Sp1* failed to improve hypothermic cardioprotection in *Cirbp*-knockout aged donor hearts during cold storage in transplantation. (A) Experimental design. *Cirbp*-knockout aged rats (1-year-old) were injected with AAV9 vectors carrying *Sp1* cDNA (AAV9-*Sp1*) or control vehicles (AAV9-null) (n=5 in each group). After 4 weeks, the donor hearts were harvested from two groups and transplanted to the wild-type young rats (10-week-old). Hypothermia was used to protect the donor heart during cold storage. The beating score was evaluated at 30 mins after transplantation. The donor heart functions and myocardial injury indicators were measured at 1 day after transplantation. SD, Sprague-Dawley. **(B)** Western blotting and quantification of SP1 in donor hearts. **(C)** Assessment of cardiac

resuscitation after transplantation. **(D)** Cardiac function of donor hearts measured by echocardiography after transplantation. **(E)** Representative left ventricular pressure (LVP) traces and cardiac function parameters of donor hearts determined via cardiac catheterization after transplantation. LVEDP, left ventricular end-diastolic pressure. **(F)** Serum cardiac enzymes in recipients after transplantation. **(G)** MPO staining of donor hearts after transplantation. Scale bars, 50 μm. **(H)** H&E and PTAH staining of donor hearts after transplantation. The arrows indicate myocardial contraction band necrosis. Scale bars, 100μm in H&E and 50μm in PTAH. **(I)** Lipid peroxidation in donor hearts after transplantation. **(J)** Prussian blue staining of donor hearts after transplantation. The arrows indicate iron deposition in myocardium. Scale bars, 50 μm. **(K)** Western blotting and quantification of PTGS2 in donor hearts after transplantation. **(L)** Transmission electron microscopy images of donor hearts after transplantation. Blue arrows indicate mitochondrial shrinkage; Black arrows indicate the mitochondrial crista loss; Red arrows indicate mitochondrial membrane rupture. Scale bars, 1 μm. The data shown are the mean ± standard deviation with individual values presented as a dot plot. Statistical significance of beating score was determined with Mann-Whitney test. Statistical significance of the other parameters was determined with Student's *t* test (NS, not significant).

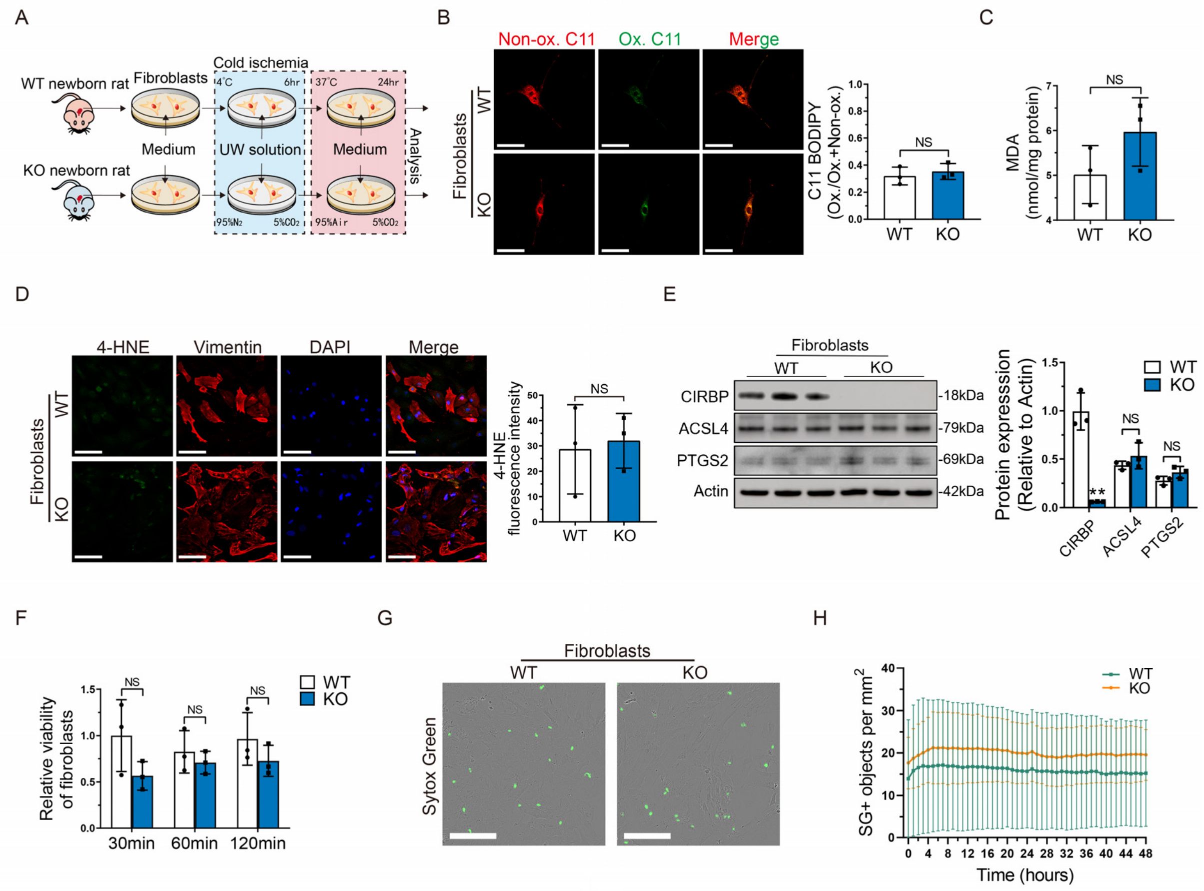

Supplemental Figure 14. The effect of CIRBP on ferroptosis susceptibility after cold ischemia is not significant in cardiac fibroblasts. (A) Experimental design. The cardiac fibroblasts were isolated from the wild-type or *Cirbp*-knockout neonatal rats and subjected to cold ischemia. Both groups were treated with UW solution during cold ischemia. **(B)** Fibroblasts labelled with BODIPY 581/591 C11 after cold ischemia. The graph shows the ratio of oxidized-to-total BODIPY 581/591 C11. Ox., oxidized; Non-ox., nonoxidized. Scale bar, 25μm. **(C)** The levels of MDA in fibroblasts after cold ischemia. **(D)** 4-HNE staining of fibroblasts after cold ischemia. Scale bar, 25μm. **(E)** Western blotting and quantification of CIRBP, ACSL4, and PTGS2 in fibroblasts after cold ischemia. **(F)** Cell viability of fibroblasts after cold ischemia. **(G)** Live-cell imaging of fibroblasts incubated with SYTOX Green after cold ischemia. Scale bar, 300 μm. **(H)** Time-lapse analysis of cell death in fibroblasts after cold ischemia. SG, SYTOX Green. The data shown are the mean ± standard deviation with individual values presented as a dot plot. Statistical significance was determined with Student’s t test (NS, not significant).

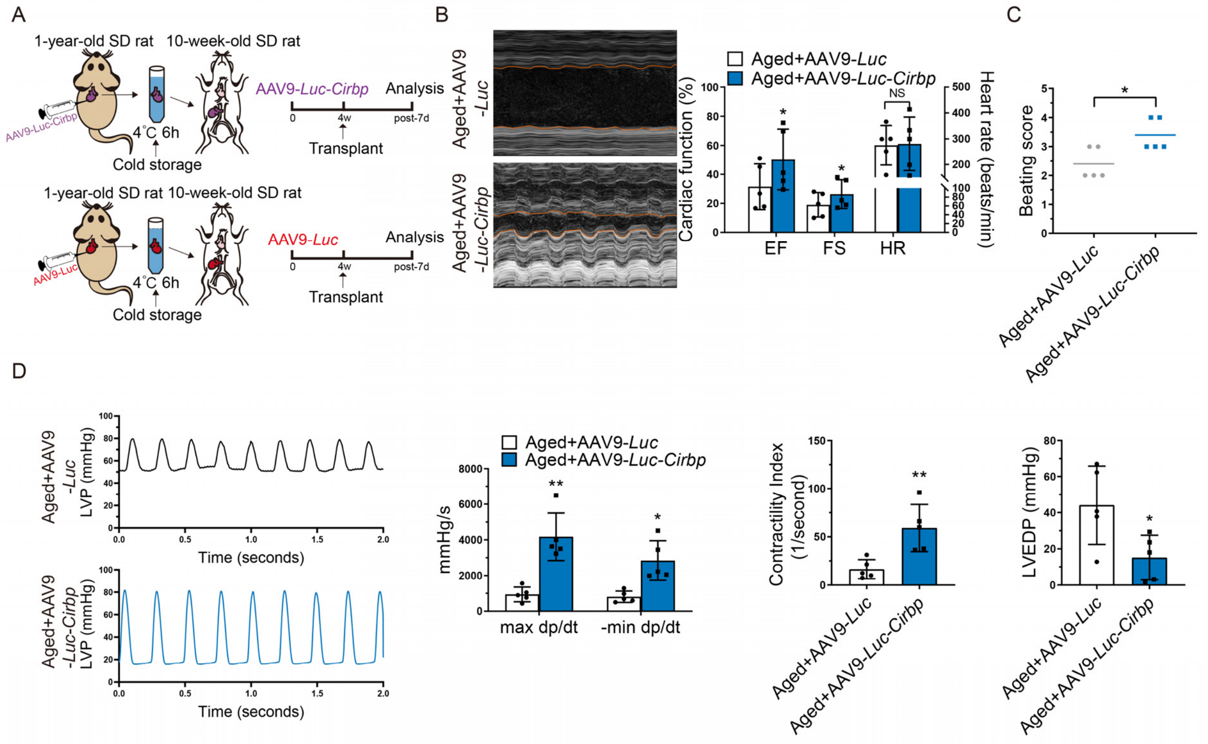

Supplemental Figure 15. *Cirbp*-overexpressing aged donor hearts showed improved cardiac functions at 7 days after transplantation. (**A**) Experimental design. Aged rats (1-year-old) were injected with AAV9 vectors carrying *Cirbp* cDNA (AAV9-*Luc*-*Cirbp*) or control vehicles (AAV9-*Luc*) (n=5 in each group). After 4 weeks, the donor hearts were harvested from two groups and transplanted to the young rats (10-week-old). Hypothermia was used to protect the donor heart during cold storage. The beating score and donor heart function were measured at 7 days after transplantation. SD, Sprague-Dawley. (**B**) Cardiac function of donor hearts measured by echocardiography at 7 days after transplantation. EF, ejection fraction; FS, fractional shortening; SV, stoke volume; CO, cardiac output; HR, heart rate. **(C)** Beating score of donor hearts at 7 days after transplantation. (**D**) Representative left ventricular pressure (LVP) traces and cardiac function parameters of donor hearts determined via cardiac catheterization at 7 days after transplantation. LVEDP, left ventricular end-diastolic pressure. Quantitative data are shown as the mean ± standard deviation with individual values presented as a dot plot. Statistical significance of beating score was determined with Mann-Whitney test. Statistical significance of the other parameters was determined with Student's *t* test (**P* < 0.05; ***P* < 0.01; NS, not significant).

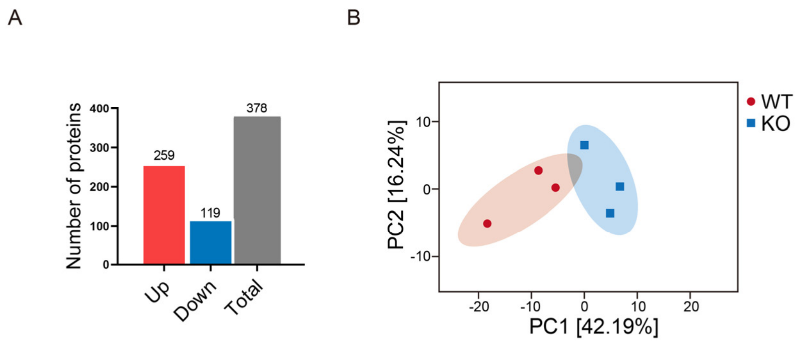

Supplemental Figure 16. Proteomic analysis of wild-type and *Cirbp*-knockout donor hearts after transplantation. (A) The total numbers of proteins whose expression were significantly increased or decreased between two groups; **(B)** PCA plots showed the separation of clusters between the two groups (n=3 in each group).

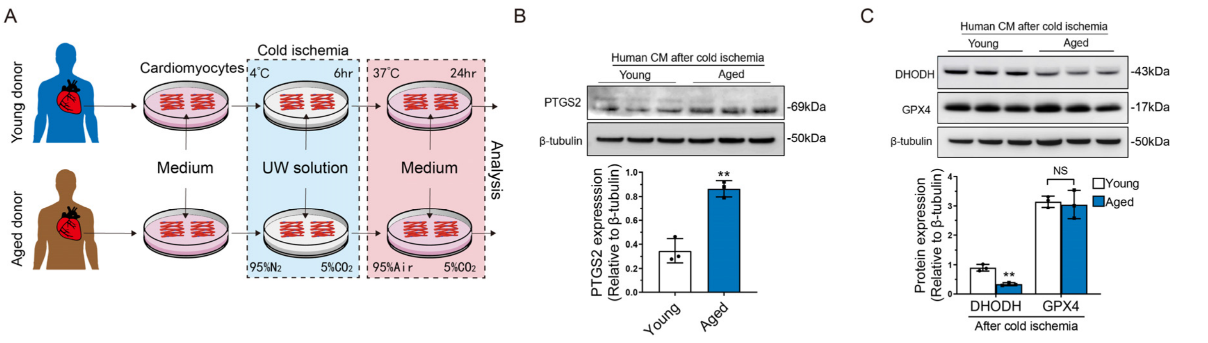

Supplemental Figure 17. Ferroptosis is exacerbated in aged human cardiomyocytes after cold ischemia. (**A**) Experimental design. The cardiomyocytes were isolated from the young or aged human donor heart and subjected to cold ischemia. Both groups were treated with UW solution during cold ischemia. (**B**) Western blotting and quantification of PTGS2 in human cardiomyocytes after cold ischemia. (**C**) Western blotting and quantification of DHODH and GPX4 in human cardiomyocytes after cold ischemia. The data shown are the mean ± standard deviation with individual values presented as a dot plot. Statistical significance was determined with Student's t test (***P* < 0.01; NS, not significant).

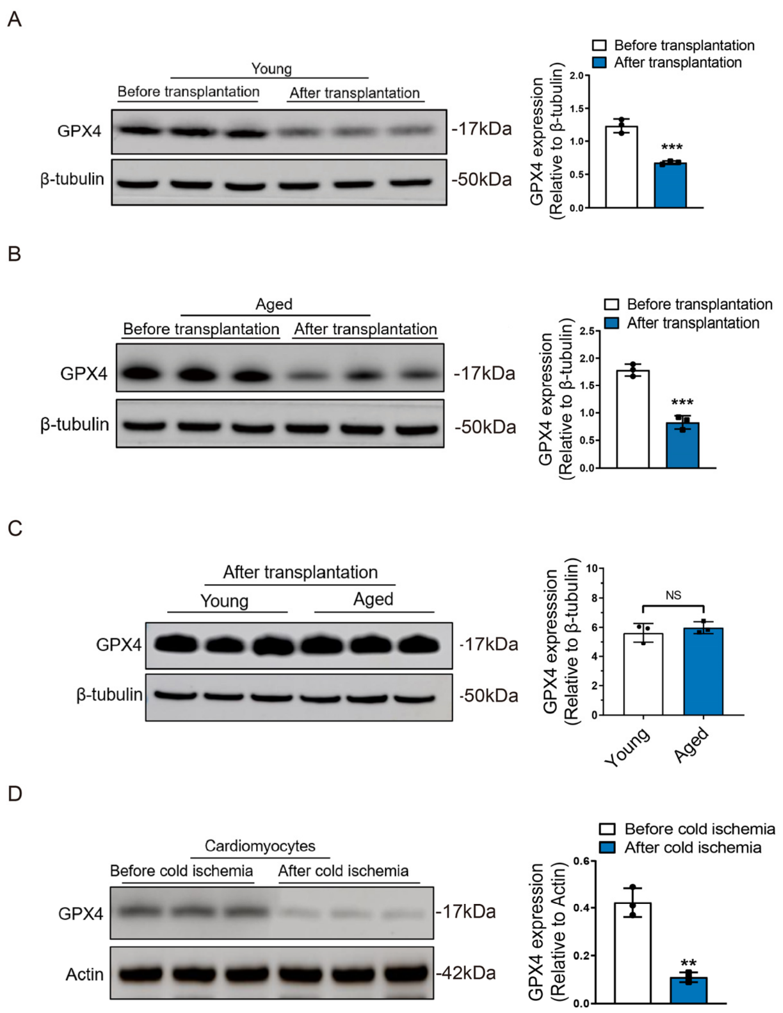

Supplemental Figure 18. GPX4-mediated ferroptosis defense is compromised during heart transplantation. (**A**) Western blotting and quantification of GPX4 in the young donor heart before and after transplantation. (**B**) Western blotting and quantification of GPX4 in the aged donor heart before and after transplantation. (**C**) Western blotting and quantification of GPX4 in the young and aged donor heart after transplantation. (**D**) Western blotting and quantification of GPX4 in the cardiomyocytes before and after cold ischemia. The data shown are the mean ± standard deviation. Statistical significance was analyzed by Student's *t* test (**$P < 0.01$; ***$P < 0.001$; NS, not significant).

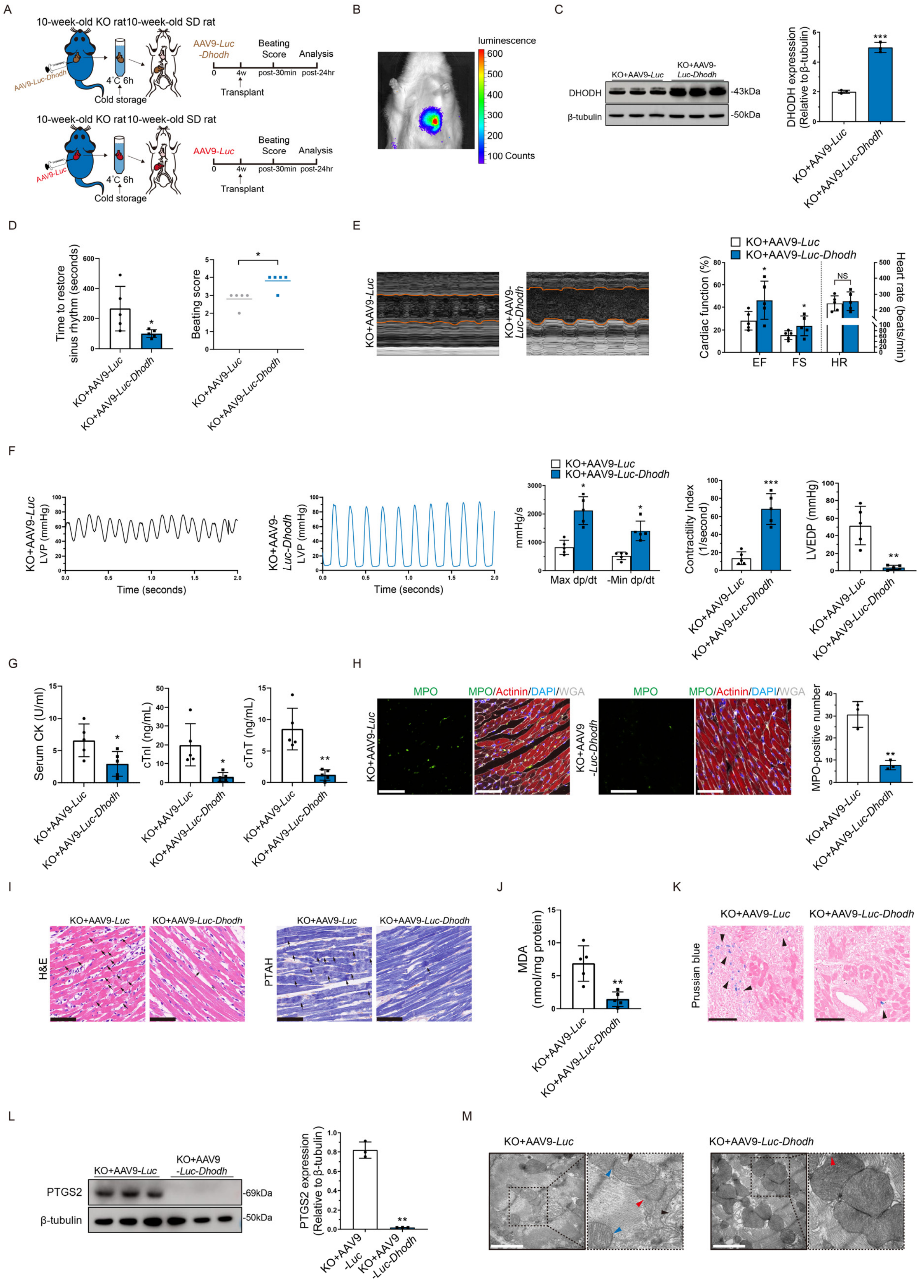

Supplemental Figure 19. Overexpression of *Dhodh* improves hypothermic cardioprotection in *Cirbp*-knockout young donor hearts during cold storage in transplantation. (A) Experimental design. An AAV9 vector carrying the *cTNT* promoter to drive the expression of firefly luciferase and *Dhodh* (AAV9-

Luc-Dhodh) was injected into *Cirbp*-knockout young rats (10-week-old), and an AAV9 vector carrying firefly luciferase (AAV9-*Luc*) was used as a control (n=5 in each group). After 4 weeks, the donor hearts were harvested from two groups and transplanted to the wild-type young rats (10-week-old). Hypothermia was used to protect the donor heart during cold storage. The beating score was evaluated at 30 mins after transplantation. The donor heart functions and myocardial injury indicators were measured at 1 day after transplantation. SD, Sprague-Dawley. **(B)** In vivo imaged of luciferase in aged rats after AAV9 transfection. **(C)** Western blotting and quantification of DHODH in donor hearts after transplantation. **(D)** Assessment of cardiac resuscitation after transplantation. **(E)** Cardiac function of donor hearts measured by echocardiography after transplantation. **(F)** Representative left ventricular pressure (LVP) traces and cardiac function parameters of donor hearts determined via cardiac catheterization after transplantation. LVEDP, left ventricular end-diastolic pressure. **(G)** Serum cardiac enzymes in recipients after transplantation. **(H)** MPO staining of donor hearts after transplantation. Scale bars, 50 μm. **(I)** H&E and PTAH staining of donor hearts after transplantation. The arrows indicate myocardial contraction band necrosis. Scale bars, 100 μm in H&E and 50 μm in PTAH. **(J)** Lipid peroxidation in donor hearts after transplantation. **(K)** Prussian blue staining of donor hearts after transplantation. The arrows indicate iron deposition in myocardium. Scale bars, 50 μm. **(L)** Western blotting and quantification of PTGS2 in donor hearts after transplantation. **(M)** Transmission electron microscopy images of donor hearts after transplantation. Blue arrows indicate mitochondrial shrinkage; Black arrows indicate the mitochondrial crista loss; Red arrows indicate mitochondrial membrane rupture. Scale bars, 1 μm. The data shown are the mean ± standard deviation with individual values presented as a dot plot. Statistical significance of beating score was determined with Mann-Whitney test. Statistical significance of the other parameters was determined with Student's t test (*$P < 0.05$; **$P < 0.01$, ***$P < 0.001$; NS, not significant).

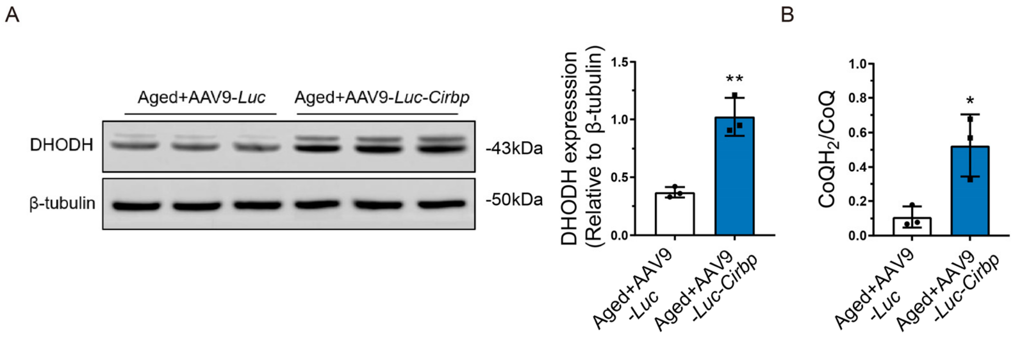

Supplemental Figure 20. Overexpression of *Cirbp* enhances DHODH expression and CoQ reduction in aged donor heart. (**A**) Western blotting and quantification of DHODH in aged donor hearts with or without overexpression of *Cirbp* after transplantation. (**B**) The $CoQH_2$/CoQ ratio in aged donor hearts with or without overexpression of *Cirbp* after transplantation. The data shown are the mean ± standard deviation. Statistical significance was analyzed by Student's *t* test (*$P < 0.05$; **$P < 0.01$).

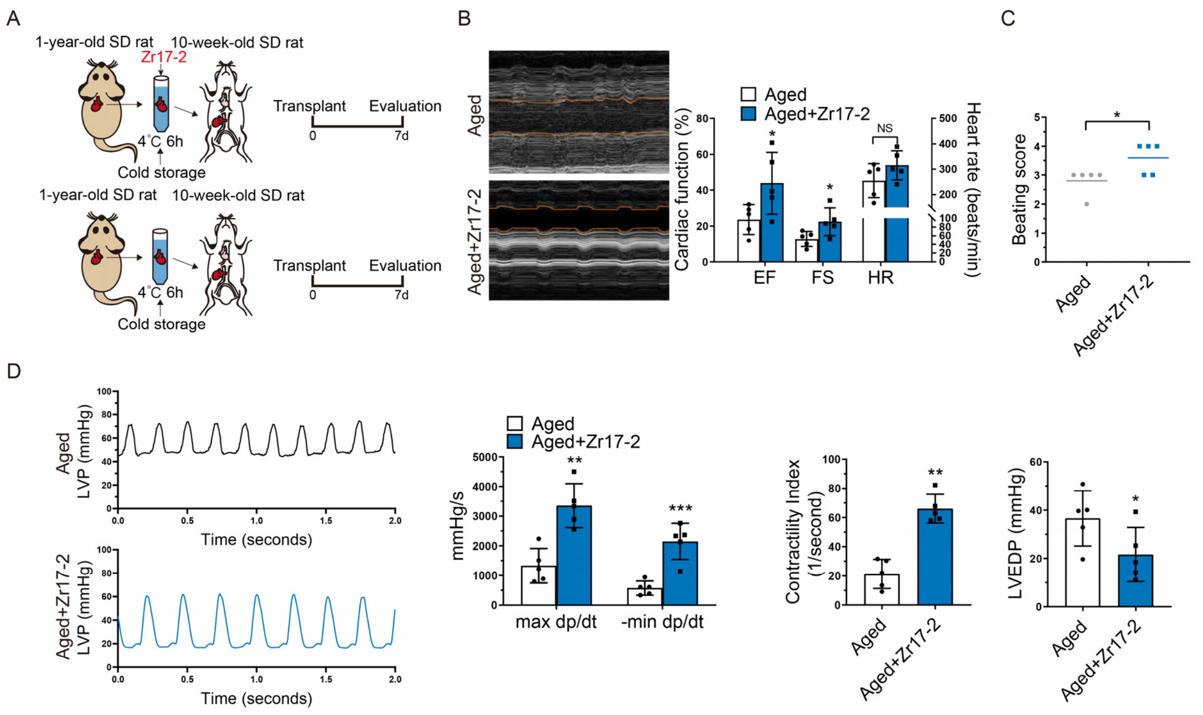

Supplemental Figure 21. *Cirbp* agonist improved cardiac functions of aged donor hearts at 7 days after transplantation. (A) Experimental design. Donor hearts were harvested from aged rats (1-year-old). UW solution was used to induce cardiac arrest in control group, and the experimental group used UW solution supplemented with *Cirbp* agonist (Zr17-2) (n=5 in each group). After 6-hours cold ischemia, donor hearts were transplanted to the young rat (10-week-old). Hypothermia was used to protect the donor heart during cold storage. The donor heart functions and myocardial injury indicators were measured at 7 days after transplantation. SD, Sprague-Dawley. (**B**) Cardiac function of donor hearts measured by echocardiography at 7 days after transplantation. EF, ejection fraction; FS, fractional shortening; SV, stoke volume; CO, cardiac output; HR, heart rate. **(C)** Beating score of donor hearts at 7 days after transplantation. (**D**) Representative left ventricular pressure (LVP) traces and cardiac function parameters of donor hearts determined via cardiac catheterization at 7 days after transplantation. LVEDP, left ventricular end-diastolic pressure. Quantitative data are shown as the mean ± standard deviation with individual values presented as a dot plot. Statistical significance of beating score was determined with Mann-Whitney test. Statistical significance of the other parameters was determined with Student's t test (*$P < 0.05$; **$P < 0.01$; ***$P < 0.001$; NS, not significant).

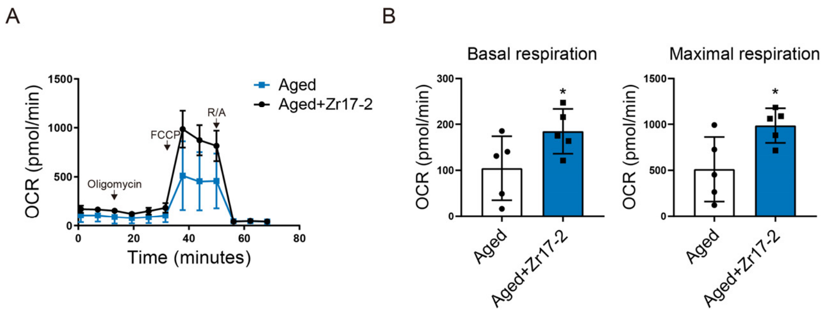

Supplemental Figure 22. Cardioplegic solution supplemented with *Cirbp* agonist could improve mitochondrial functions of aged donor heart at 1 day after transplantation. (**A**) Oxygen consumption rate (OCR) of cardiomyocytes isolated from aged donor heart or Zr17-2 treated aged donor heart at 1 day after transplantation. The traces were recorded by XF24 metabolic analyzer and the mitochondrial effectors were injected at time points indicated by downward arrows. (**B**) Basal respiration and maximal respiration of cardiomyocytes isolated from two groups. Statistical significance was analyzed by Student's *t* test (*$P <$ 0.05).

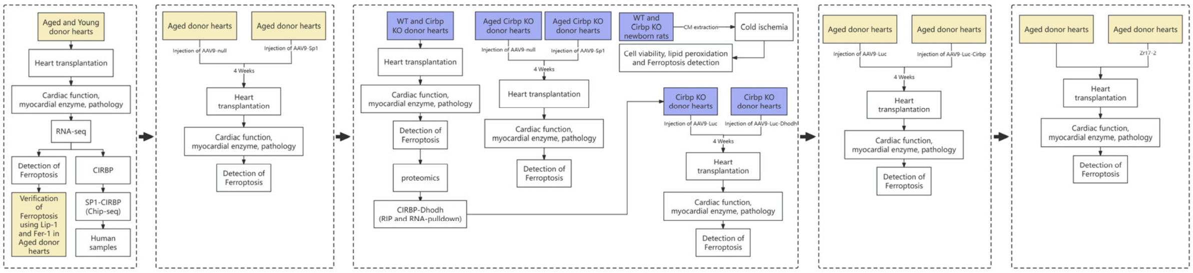

Supplemental Figure 23. Research process flowchart in our study. The brown section focused on phenotypes of aged donor heart after transplantation. The blue section focused on exploring its underlying mechanisms.

Supplemental Tables

Supplemental Table 1. Baseline characteristics of human donors.

	**Young donors**	**Aged donors**
Number	7	5
Age (year)	25.0±5.1	52.4±6.9
Male (%)	71.4	80.0
LVEF (%)	68.0±6.5	68.2±8.9
LVFS (%)	35.0±3.9	35.1±3.5

LVEF, left ventricular ejection fraction; LVFS, left ventricular fractional shortening. Data are mean ± standard deviation.

Supplemental Table 2. Significantly different proteins (fold change < 0.83 or fold change > 1.2, P < 0.05) in young donor hearts from Cirbp-knockout rats versus wild-type rats.

Protein	Description	*P* value	Fold Change
Carhsp1	Calcium-regulated heat stable protein 1	0.000357348	0.806884621
Rnase2	Pre-eosinophil-associated ribonuclease-2	0.000392298	1.72354049
Plcd1	1-phosphatidylinositol 4,5-bisphosphate phosphodiesterase delta-1	0.000631008	0.423643054
Cirbp	Cold-inducible RNA-binding protein	0.000700132	0.519144505
LOC100911104	Ly6-C antigen	0.000714226	1.559586585
Hdhd5	Cat eye syndrome chromosome region, candidate 5 homolog (Human) (Predicted)	0.001100373	0.794621938
Fcgrt	IgG receptor FcRn large subunit p51	0.001244084	0.722510996
Sqor	Sqrdl protein	0.001280181	1.304696804
Reep5	Receptor expression-enhancing protein 5	0.001399164	0.826194823
Ptms	Parathymosin	0.001402497	0.708204388
Rfx1	Regulatory factor X, 1 (Influences HLA class II expression) (Predicted)	0.001583487	0.8176
8-Sep	Septin-8	0.001624336	0.741751844
Ftl1	Ferritin light chain 1	0.001826433	1.298472635
Atp2a3	Calcium-transporting ATPase	0.001866854	1.51886121
Serpinb6a	Serine (Or cysteine) peptidase inhibitor, clade B, member 6a	0.001895052	0.818151958
Gstz1	Maleylacetoacetate isomerase	0.002078924	1.318547574
Rab3ip	Rab-3A-interacting protein	0.002086068	0.717789598
Mboat7l1	Membrane-bound O-acyltransferase domain-containing 7-like 1	0.002151507	1.241301908
Lama4	Laminin subunit alpha 4	0.002197673	0.816537933
Col1a1	Collagen alpha-1(I) chain	0.002216522	0.751495176
Cltb	Clathrin light chain B	0.002224421	0.821035371
Atxn10	Ataxin-10	0.002349256	0.825367906
Col5a1	Collagen alpha-1(V) chain	0.00250061	0.71863343
Fdx1	Adrenodoxin, mitochondrial	0.002519292	0.828398668
Myom3	Myomesin 3	0.002640647	0.684265791
Cox19	Cytochrome c oxidase assembly factor COX19	0.003051766	0.779365476
Chaf1b	Chromatin assembly factor 1 subunit B	0.003129556	1.244206774
Tlr2	Toll-like receptor 2	0.00316159	1.260284126
Mprip	Myosin phosphatase Rho-interacting protein	0.003277223	0.816554589
Micall2	MICAL-like protein 2	0.0032976	1.243982048
Rida	2-iminobutanoate/2-iminopropanoate deaminase	0.00332536	1.299059929
Fbn1	Fibrillin 1	0.003676915	0.825282
Dhodh	Dihydroorotate dehydrogenase (quinone), mitochondrial	0.004077	0.6660152
Igfbp7	Insulin-like growth factor binding protein 7, isoform CRA_b	0.004095496	0.822652677
Bnip2	BCL2-interacting protein 2	0.004166827	1.296583851
Atp1a2	Sodium/potassium-transporting ATPase subunit alpha-2	0.00438137	0.817238357
Nedd8	NEDD8	0.004561838	0.812956126
Ext2	Exostoses (Multiple) 2 (Predicted)	0.004661652	0.751808336
crp	Pentaxin	0.004752396	1.432377737
Glrx5	Glutaredoxin 5	0.004760031	0.811717912
Slpi	Secretory leukocyte protease inhibitor	0.004812315	3.459562965

Supplemental Table 2. continued

Protein	Description	*P* value	Fold Change
Rbfox1	RNA binding protein fox-1 homolog 1	0.004848827	0.790358433
Fcer1g	Fc receptor gamma-chain	0.004954012	1.905592303
Prkaca	cAMP-dependent protein kinase	0.005015206	0.659816312
Postn	Periostin	0.005034577	0.58492317
Dpep1	Dipeptidase 1	0.005094596	0.809884718
Hsd11b1	Corticosteroid 11-beta-dehydrogenase isozyme 1	0.005110183	0.67069435
Rac2	Rac family small GTPase 2	0.005136074	1.376295933
Col3a1	Collagen alpha-1(III) chain	0.005486285	0.611683598
Ndufv3	Complex I-9kD	0.005549151	0.62040515
Pak1ip1	PAK1-interacting protein 1	0.005789572	1.294985251
Crispld2	Cysteine-rich secretory protein LCCL domain-containing 2	0.005832119	1.349580641
Hk3	Hexokinase	0.00627993	1.233980949
RGD1559575	Similar to novel protein	0.006921701	1.306364252
Cox17	Cytochrome C oxidase assembly protein COX17	0.006979744	0.667207695
Ifrd1	Interferon-related developmental regulator 1	0.007338004	1.376881397
Caskin2	Cask-interacting protein 2	0.007419579	0.816009214
Myh15	Myosin, heavy chain 15	0.007849948	0.688738979
Tf	Serotransferrin	0.007955	1.3183
Rptor	Regulatory-associated protein of MTOR, complex 1	0.008047063	1.252144082
Bola3	BolA family member 3	0.008278832	0.828538986
Impa2	Inositol-1-monophosphatase	0.008305223	0.805244991
Serpinb10	Serine (Or cysteine) peptidase inhibitor, clade B (Ovalbumin), member 10	0.008445471	1.448928239
Dock8	Dedicator of cytokinesis 8	0.008568186	1.211004896
Chrac1	Chromatin accessibility complex 1 (Predicted), isoform CRA_b	0.00858366	1.204308094
Apoc4	Apolipoprotein C-IV	0.008593371	1.746014172
Tpm2	Tropomyosin beta chain	0.008645568	0.390846497
Pkia	cAMP-dependent protein kinase inhibitor alpha	0.008700131	0.657725622
Rilp	Rab interacting lysosomal protein (Predicted)	0.008729554	1.219645686
Arhgap45	Histocompatibility (Minor) HA-1 (Predicted), isoform CRA_b	0.00879689	1.416170213
Smim20	Small integral membrane protein 20	0.008920874	0.657824622
Proz	Proz protein (Fragment)	0.008994493	1.383093605
Gss	Glutathione synthetase	0.009	0.829155591
C1r	Complement subcomponent C1r	0.009012408	1.297393247
Nqo1	NAD(P)H dehydrogenase [quinone] 1	0.009216552	1.365541686
Ncf4	Ncf4 protein	0.009306897	1.442348704
Hcls1	Hematopoietic cell-specific LYN substrate 1	0.009430019	1.541909392
Parvg	Parvin, gamma	0.009474131	1.344815825
Lyz1	Lysozyme C-1	0.00948102	1.853005452
Acap2	Arf-GAP with coiled-coil, ANK repeat and PH domain-containing protein 2	0.009588188	1.207818431
Stxbp2	Syntaxin-binding protein 2	0.009708779	1.433068685
Saa4	Hermansky-Pudlak syndrome 5 protein homolog	0.009919368	1.658018156
Apom	Apolipoprotein M	0.010105965	1.293233083

Supplemental Table 2. continued

Protein	Description	*P* value	Fold Change
Cfp	Complement factor properdin	0.010129203	1.557110463
Ppic	Peptidyl-prolyl cis-trans isomerase	0.010129827	0.829491282
Stk4	Non-specific serine/threonine protein kinase	0.010134889	1.2607152
Hpcal1	Hippocalcin-like protein 1	0.01024535	1.217133942
Bop1	Ribosome biogenesis protein BOP1	0.010284617	1.264876957
C6	Complement component C6	0.010285843	1.529652259
Rab27a	Ras-related protein Rab-27A	0.010505139	1.43456944
Cap1	Adenylyl cyclase-associated protein 1	0.010610102	1.327941103
Uxs1	UDP-glucuronic acid decarboxylase 1	0.011084429	1.208603418
Diaph1	Protein diaphanous homolog 1	0.011162487	1.216110767
Sars2	Seryl-tRNA synthetase	0.011389315	0.826539235
Zfp512	Zinc finger protein 512	0.011401846	0.768220617
Taco1	Translational activator of cytochrome c oxidase 1	0.011418254	0.820128539
Gtpbp8	GTP-binding protein 8	0.011556099	0.672977625
Ethe1	ETHE1, persulfide dioxygenase	0.011562953	1.235888552
Serpina10	Protein Z-dependent protease inhibitor	0.011568331	1.522795997
Eln	Elastin	0.011624167	0.796766241
Fgr	Tyrosine-protein kinase Fgr	0.011680857	1.467010632
Ptpn1	Tyrosine-protein phosphatase non-receptor type 1	0.011885521	1.227218029
Arg1	Arginase-1	0.012220604	1.532024793
Nutf2	Nuclear transport factor 2	0.012333948	0.828567294
Skap2	Src kinase-associated phosphoprotein 2	0.012629513	1.492045859
Vasp	Vasodilator-stimulated phosphoprotein	0.012658441	1.3211021
Chchd1	Coiled-coil-helix-coiled-coil-helix domain containing 1 (Predicted), isoform CRA_a	0.012699806	0.712661652
Lypla1	Acyl-protein thioesterase 1	0.012991773	0.676635134
Card9	Caspase recruitment domain-containing protein 9	0.013087515	1.323317218
Cygb	Cytoglobin, isoform CRA_b	0.013225046	0.759716175
Timm8b	Mitochondrial import inner membrane translocase subunit Tim8 B	0.013410298	0.815067311
Wdr3	WD repeat domain 3	0.013481295	1.214835549
Slc35f6	Solute carrier family 35 member F6	0.013890428	1.374153205
Fkbp3	Peptidylprolyl isomerase (Fragment)	0.014029436	0.79601358
Junb	Transcription factor jun-B	0.01403794	1.739039118
Akap12	A-kinase anchor protein 12	0.014606757	1.387475697
Pros1	Vitamin K-dependent protein S	0.014663602	1.488754243
Pnkp	Polynucleotide kinase 3-phosphatase	0.014695105	1.238055843
Pcsk5	Proprotein convertase subtilisin/kexin type 5	0.014882408	1.397538016
Tmem38a	Transmembrane protein 38a (Predicted), isoform CRA_b	0.014887688	0.718571394
Gpx3	Glutathione peroxidase	0.015265743	1.564650059
Ncf2	Neutrophil cytosolic factor 2	0.015399965	1.517204301
Fam173a	Adenine nucleotide translocase lysine methyltransferase	0.015565093	0.765819362
Arhgdib	Rho GDP dissociation inhibitor beta	0.015568416	1.598965201
Ptpn6	Tyrosine-protein phosphatase non-receptor type 6	0.015569331	1.352380462

Supplemental Table 2. continued

Protein	Description	*P* value	Fold Change
Itgal	Integrin subunit alpha L	0.015579264	1.355223881
Gmfg	Glia maturation factor	0.015645707	1.593476556
Cybb	Cytochrome b-245 beta chain	0.015661204	1.732658202
Apbb1ip	Amyloid beta (A4) protein-binding, family B, member 1 interacting protein	0.0157474	1.409265176
Vrk1	VRK serine/threonine kinase 1	0.015747605	1.41791905
Cdh1	Cadherin-1	0.015929981	1.353670635
Pde2a	Phosphodiesterase	0.015959884	0.77900251
Glrx	Glutaredoxin-1	0.015995288	1.233626517
Prxl2a	Peroxiredoxin-like 2A	0.016135726	1.351494314
Ptp4a3	Protein tyrosine phosphatase 4A3	0.016156447	0.740780331
Plin4	Perilipin 4	0.016246167	0.789079256
Cd74	H-2 class II histocompatibility antigen gamma chain	0.016292471	1.25904928
Blvra	Biliverdin reductase A	0.016461966	0.544996457
Mgp	Matrix Gla protein	0.016515283	1.387571366
Cavin4	Caveolae-associated protein 4	0.01658518	0.813106703
Ercc1	ERCC excision repair 1, endonuclease non-catalytic subunit	0.016625624	0.824362606
Plg	Plasminogen	0.016667869	1.624145911
G6pdx	Glucose-6-phosphate 1-dehydrogenase	0.016702488	1.474996357
Il1rn	Interleukin-1 receptor antagonist protein	0.016707704	1.397872451
Zyx	Zyxin	0.016735179	1.364610882
Fcn2	Ficolin-2	0.016950252	1.444581573
Pstpip2	Proline-serine-threonine phosphatase-interacting protein 2	0.017108117	1.284993205
Habp2	Hyaluronan-binding protein 2	0.017195431	1.506952224
Lta4h	Leukotriene A-4 hydrolase	0.017211624	1.326896235
Myo1g	Myosin IG	0.017305059	1.725971925
Pgd	6-phosphogluconate dehydrogenase, decarboxylating	0.017538155	1.312363663
Ece1	Endothelin-converting enzyme 1	0.017766728	1.217772215
Tns2	Tensin 2	0.017818351	0.707390917
Nova2	NOVA alternative-splicing regulator 2	0.018113742	0.820935243
Akr1b8	Aldose reductase-like protein	0.01815684	1.531345337
Smim12	Small integral membrane protein 12	0.018190957	0.797457418
Naca	Nascent polypeptide-associated complex subunit alpha	0.018202794	0.818163873
Rpp30	Ribonuclease P/MRP subunit p30	0.018541311	1.584429825
F13b	Coagulation factor XIII B chain	0.018559641	1.245120539
Hspb3	Heat shock 27kDa protein 3	0.018562075	0.748011433
Tes	Testin	0.018765179	1.237816151
Lcat	Phosphatidylcholine-sterol acyltransferase	0.018771058	1.421361232
Iqgap2	IQ motif-containing GTPase-activating protein 2	0.018960414	1.213555045
C4bpa	C4b-binding protein alpha chain	0.019202361	1.679114638
Ube2b	Ubiquitin-conjugating enzyme E2 B	0.019277318	0.6
Arpc1b	Actin-related protein 2/3 complex subunit 1B	0.019409062	1.338325098
C4a	C4a anaphylatoxin	0.019605429	1.368485457

Supplemental Table 2. continued

Protein	Description	*P* value	Fold Change
Mrpl35	39S ribosomal protein L35, mitochondrial	0.019816419	1.255266758
Arhgap9	Rho GTPase-activating protein 9	0.019929713	1.322326179
RGD1307182	Similar to RIKEN cDNA B430306N03 gene	0.01994467	1.537773804
C5ar1	C5a anaphylatoxin chemotactic receptor 1	0.020017261	1.411037736
Vapb	Vesicle-associated membrane protein-associated protein B	0.020490697	0.814180154
Lsp1	Lymphocyte specific 1, isoform CRA_a	0.021247277	1.528324996
Apcs	Pentaxin	0.021288948	1.284102704
Pstpip1	Proline-serine-threonine phosphatase-interacting protein 1	0.021512694	1.373447731
Vtn	Vitronectin	0.02156905	1.795375023
Angel2	Angel homolog 2	0.021624217	1.205436391
NFIB	CCAAT-box-binding transcription factor (Fragment)	0.02179729	0.76209036
Samhd1	Deoxynucleoside triphosphate triphosphohydrolase SAMHD1	0.021999921	1.283372365
Vdac2	Voltage-dependent anion-selective channel protein 2	0.022	0.825719
Rps24	40S ribosomal protein S24	0.022133686	1.248169542
Cbr4	Carbonyl reductase family member 4	0.022180628	0.800367157
Myl7	Myosin light chain 7	0.022364263	1.817007168
Fmnl1	Formin-like 1	0.02241324	1.21603432
Cd34	CD34 antigen (Predicted)	0.022413491	0.798230614
Suox	Sulfite oxidase, mitochondrial	0.02262426	0.784026389
Arpc5	Actin-related protein 2/3 complex subunit 5	0.022695975	1.250077515
Rbmx	RNA-binding motif protein, X chromosome	0.022714	0.809935502
Lcp1	Lymphocyte cytosolic protein 1	0.023192758	1.443853372
Cblb	E3 ubiquitin-protein ligase CBL-B	0.023223187	1.473177442
Pcdh7	Protocadherin 7, isoform CRA_b	0.023277966	0.810787172
Ptprc	Receptor-type tyrosine-protein phosphatase C	0.023350933	1.386906353
Masp1	Mannan-binding lectin serine protease 1	0.023541302	1.236930377
F9	Coagulation factor IX	0.023590743	1.47260298
Ccdc9b	Coiled-coil domain containing 9B	0.023604963	0.789112327
Tnfaip8	TNF alpha-induced protein 8	0.023665065	1.292616372
Cfh	Complement factor H	0.02377252	1.425012388
Itgam	Integrin alpha M	0.024052332	1.486191469
Nudt3	Diphosphoinositol polyphosphate phosphohydrolase 1	0.02410264	0.809430716
Cpne2	Copine 2	0.024165858	1.299146413
Psmb10	Proteasome subunit beta type-10	0.024250048	1.20484372
Lamtor2	Late endosomal/lysosomal adaptor, MAPK and MTOR activator 2	0.0242511	1.223439074
Igf1	Insulin-like growth factor I	0.024291765	1.246931672
Rbpjl2	Recombination signal-binding protein for immunoglobulin kappa J region-like 2	0.024317214	1.549992414
Apobr	Apolipoprotein B receptor	0.024981294	1.278631588
Pum3	Pumilio homolog 3	0.02519445	1.201945227
Rpl35a	60S ribosomal protein L35a	0.025316104	1.263792917
Ass1	Argininosuccinate synthase	0.025625552	1.55330438
C8a	Complement C8 alpha chain	0.0256564	1.493093398

Supplemental Table 2. continued

Protein	Description	*P* value	Fold Change
Kcnab2	Voltage-gated potassium channel subunit beta-2	0.025875126	1.584830508
Ripk3	Receptor-interacting serine/threonine-protein kinase 3	0.026273986	1.487857485
Gsn	Gelsolin	0.026723225	1.429971547
F10	Coagulation factor X	0.026845711	1.635171981
Mustn1	Musculoskeletal embryonic nuclear protein 1	0.027074064	0.684726897
C4bpb	C4b-binding protein beta chain	0.027158632	1.567896895
Apob	Apolipoprotein B-100	0.027238252	1.438034429
Cyba	Cytochrome b-245 light chain	0.02736959	1.741042345
Prg4	Proteoglycan 4	0.027388276	1.453028072
Serpine1	Plasminogen activator inhibitor 1	0.027413633	1.699577758
Crip2	Cysteine-rich protein 2	0.027513768	0.799060148
Ctsg	Cathepsin G	0.027535544	1.882376179
Rad50	DNA repair protein RAD50	0.02756596	1.238022034
ND4L	NADH-ubiquinone oxidoreductase chain 4L	0.027569835	0.792151028
Gpd1	Glycerol-3-phosphate dehydrogenase [NAD(+)], cytoplasmic	0.027587912	0.720166945
Itih4	Inter alpha-trypsin inhibitor, heavy chain 4	0.027687666	1.499260274
Napsa	Napsin	0.027741414	1.547880737
Pccb	Propionyl coenzyme A carboxylase, beta polypeptide	0.028122925	1.292553191
Ambp	Protein AMBP	0.028293352	1.434573605
Atf1	Atf1 protein (Fragment)	0.02834681	1.23229109
LOC100361907	Cfh protein	0.028400268	1.670943228
Pram1	PML-RARA-regulated adaptor molecule 1	0.028533815	1.713229511
Lst1	Leukocyte-specific transcript 1	0.028636383	2.106400665
C8b	Complement component C8 beta chain	0.028886103	1.391890612
Sri	LOC683667 protein	0.028942551	1.292182959
Hmox1	Heme oxygenase 1	0.029	1.466435
Gca	Grancalcin	0.029194962	1.455676727
Uck2	Uridine-cytidine kinase	0.029457904	1.281648089
Tnni1	Troponin I, slow skeletal muscle	0.02949512	0.738345207
Cav1	Caveolin-1	0.029497991	0.789068079
RGD1564614	Similar to complement factor H-related protein	0.029762026	1.394444444
Serpinb1a	Leukocyte elastase inhibitor A	0.029778008	1.567314814
Bola1	BolA family member 1	0.02990774	0.820903818
Spp2	Secreted phosphoprotein 24	0.029957759	1.79047958
Tarsl2	Threonyl-tRNA synthetase	0.030041132	0.785810306
Vamp8	Vesicle-associated membrane protein 8	0.030268875	1.380330782
Serpinf1	Alpha-2 antiplasmin	0.030331141	1.333420557
ND1	NADH-ubiquinone oxidoreductase chain 1	0.030342689	0.778567044
Arpc2	Actin-related protein 2/3 complex subunit 2	0.030703246	1.202683359
Gabarap	Gamma-aminobutyric acid receptor-associated protein	0.030796472	1.351186374
Afap1l1	Actin filament-associated protein 1-like 1	0.03100964	0.819978046
Rpl15	60S ribosomal protein L15	0.03124892	1.218122454

Supplemental Table 2. continued

Protein	Description	*P* value	Fold Change
Ostm1	Osteoclastogenesis-associated transmembrane protein 1	0.031338905	1.83081688
Sptan1	Spectrin alpha chain, non-erythrocytic 1	0.03143128	0.811205057
C3	C3-beta-c	0.03167154	1.449590907
Serpinb10	Serpin B10	0.03213574	1.685349066
Rasgrp2	RAS guanyl releasing protein 2 (Calcium and DAG-regulated) (Predicted), isoform CRA_b	0.032179532	1.636470243
Uchl5	Ubiquitin carboxyl-terminal hydrolase	0.032438466	1.325406581
Mpo	Myeloperoxidase	0.032538833	1.775070426
Plin2	Perilipin	0.03260009	1.527985939
Cdc42	Cell division control protein 42 homolog	0.032764684	1.283144153
Fkbp1a	Peptidyl-prolyl cis-trans isomerase FKBP1A	0.032884752	0.778627579
Trappc8	Similar to TRS85 homolog (Predicted)	0.032983969	1.27171488
Apobec2	Apolipoprotein B editing complex 2 (Predicted), isoform CRA_a	0.032997988	0.769929311
Serpinf2	RCG33981, isoform CRA_a	0.033059748	1.598554337
Ccr1	C-C motif chemokine receptor 1	0.033081991	1.466679657
Olfm4	Olfactomedin 4	0.033640646	1.815358822
F12	Coagulation factor XII	0.033673222	1.328134421
A1i3	Alpha-1-inhibitor 3	0.033740391	1.346638936
Plcb3	1-phosphatidylinositol 4,5-bisphosphate phosphodiesterase	0.0338216	1.206502107
C4b	C4a anaphylatoxin	0.033856489	1.392423159
Tap2	Antigen peptide transporter 2	0.03406937	1.243430514
Apoc1	Apolipoprotein C-I	0.034227503	1.643069165
Fmo3	Dimethylaniline monooxygenase [N-oxide-forming] 3	0.034260982	0.829956981
Hddc3	HD domain containing 3 (Predicted), isoform CRA_b	0.034515779	0.811275644
Mrps6	Mitochondrial ribosomal protein S6	0.034889746	0.764502762
Ciapin1	Anamorsin	0.034965449	0.825451504
S100a11	Protein S100-A11	0.035095859	1.324260786
Bmp1	Metalloendopeptidase	0.035233903	1.661080074
Alox12	Alox12 protein	0.0356	1.38844
C8g	Complement C8 gamma chain	0.035663398	1.432435908
Myh8	Myosin-8	0.035702608	1.232492997
Commd5	COMM domain-containing protein 5	0.035729798	1.2784
Slc3a2	4F2 cell-surface antigen heavy chain	0.036	1.201
Pld4	Phospholipase D family, member 4	0.036387518	1.231636995
Pygl	Glycogen phosphorylase, liver form	0.036438645	1.559273496
Zfp131	Zinc finger protein 131	0.03666192	1.312865497
Bst1	ADP-ribosyl cyclase/cyclic ADP-ribose hydrolase 2	0.03666497	1.229820628
Cnn2	Calponin (Fragment)	0.036716204	1.41192592
Ltv1	Protein LTV1 homolog	0.036727782	1.236872812
Casp1	Caspase-1	0.0369461	1.260079552
Ogn	Mimecan	0.036953364	0.732633524
Ostf1	Osteoclast-stimulating factor 1	0.037070383	1.25599448

Supplemental Table 2. continued

Protein	Description	*P* value	Fold Change
Sirpa	Sirpa protein	0.037202381	1.247602668
Litaf	LPS-induced TN factor	0.037277182	1.393313555
Hpx	Hemopexin	0.037317087	1.402081908
Prkar1a	cAMP-dependent protein kinase type I-alpha regulatory subunit	0.037450218	0.739594546
Ckb	Creatine kinase B-type	0.037603144	0.701413671
Slc29a3	Equilibrative nucleoside transporter 3	0.03761487	1.249124854
Eif3f	Eukaryotic translation initiation factor 3 subunit F	0.037647063	1.546193399
A1m	Alpha-1-macroglobulin	0.037741616	1.396635311
Papola	Poly(A) polymerase	0.037746372	1.221815623
Rcn3	Reticulocalbin-3	0.037775907	0.811738504
Ano10	Anoctamin	0.037845565	1.251106195
Cfhr2	Complement factor H-related 2	0.037880889	1.747255261
Abcc3	Canalicular multispecific organic anion transporter 2	0.038012646	1.289534766
Crym	Ketimine reductase mu-crystallin	0.038264131	0.78772022
Ptpn2	Tyrosine-protein phosphatase non-receptor type 2	0.038790201	1.297441365
Mmp8	Neutrophil collagenase	0.039063961	1.519208605
Rab6a	RCG39700, isoform CRA_d	0.039449312	1.232817869
C9	Complement component C9	0.039450553	1.414654576
Hyi	Putative hydroxypyruvate isomerase	0.039566925	0.812384005
Naaa	N-acylethanolamine-hydrolyzing acid amidase	0.039760391	1.366931352
Ublcp1	Ubiquitin-like domain-containing CTD phosphatase 1	0.039810959	1.229155163
Angptl4	Angiopoietin-related protein 4	0.039859457	1.442830417
Thbs1	Thrombospondin 1	0.040001828	1.624418455
C1qc	Complement C1q subcomponent subunit C	0.040029579	1.29279002
Hck	Tyrosine-protein kinase HCK	0.040130665	1.209816904
Ecm1	Extracellular matrix protein 1	0.040198337	1.40836204
MGC105649	Hypothetical LOC302884	0.040327119	1.596535853
Gstt2	Glutathione transferase	0.040344788	0.812063853
Apoe	Apolipoprotein E	0.040452378	1.444034628
Bgn	Biglycan	0.040498795	0.779261341
Fermt3	Fermitin family member 3	0.04063047	1.453953443
Kidins220	Kinase D-interacting substrate of 220 kDa	0.040667755	1.345098039
Nat1	Arylamine N-acetyltransferase	0.041242089	0.74852071
Pkm	Pyruvate kinase	0.041393295	1.202248235
Apoc2	Apolipoprotein C-II	0.04159402	1.400470172
Trip13	Pachytene checkpoint protein 2 homolog	0.041782297	1.273031826
Far1	Fatty acyl-CoA reductase	0.042165112	1.205340949
Cpne1	Copine-1	0.042273385	1.363584658
Arpc4	Actin-related protein 2/3 complex subunit 4	0.042698393	1.236062746
Itih3	Inter-alpha-trypsin inhibitor heavy chain H3	0.042727181	1.370742233
Stk19	Serine/threonine kinase 19 (Fragment)	0.042739113	1.28515625
Soat1	O-acyltransferase	0.042787949	1.451935081

Supplemental Table 2. continued

Protein	Description	*P* value	Fold Change
Blvra	Biliverdin reductase A	0.043000957	1.417810672
Fgl1	Fibrinogen-like protein 1	0.043039341	2.159038931
Serping1	Plasma protease C1 inhibitor	0.043247763	1.300132278
Camp	Cathelicidin antimicrobial peptide	0.043411165	1.809623431
H4c2	Histone H4	0.043479288	1.211853007
Afm	Afamin	0.043915503	1.317000464
Alox15	Polyunsaturated fatty acid lipoxygenase ALOX15	0.0444	1.39692
Apoc3	Apolipoprotein C-III	0.04444722	1.283686771
Rps6ka6	Non-specific serine/threonine protein kinase	0.044477303	1.343501326
Alox5	Polyunsaturated fatty acid 5-lipoxygenase	0.044529171	1.384967262
Cp	Ceruloplasmin	0.044646895	1.346199549
Stxbp5	Syntaxin-binding protein 5	0.044718801	1.394270339
Arhgap25	Rho GTPase-activating protein 25	0.045004931	1.30567221
Rgs14	Regulator of G-protein signaling 14	0.045444603	1.272886638
Pld6	Phospholipase D family, member 6	0.045772388	0.814091248
Tnxb	Tnxb protein (Fragment)	0.045839015	0.821323584
Anxa1	Annexin A1	0.045897536	1.728770284
Fbln1	Fibulin-1	0.04594123	1.425467614
Igfals	Insulin-like growth factor-binding protein complex acid labile subunit	0.046031733	1.451052699
Ubxn4	UBX domain-containing protein 4	0.046179362	0.808398334
Rpl34	60S ribosomal protein L34	0.046382918	1.274526914
Ncam1	Neural cell adhesion molecule 1	0.046573664	0.808440711
Dis3l2	DIS3-like exonuclease 2	0.046575169	1.305642633
Iffo1	Intermediate filament family orphan 1	0.046605815	1.814543579
Sh3pxd2b	SH3 and PX domains 2B	0.047729973	1.249520961
Faf1	FAS-associated factor 1	0.047845868	1.357539549
Slc9a3r1	Na(+)/H(+) exchange regulatory cofactor NHE-RF1	0.047862002	1.273049645
Adssl1	Adenylosuccinate synthetase isozyme 1	0.047863763	0.767756864
Gpsm3	G-protein-signaling modulator 3	0.047998578	1.489547885
Aco1	Cytoplasmic aconitate hydratase	0.048150024	1.513326752
Clu	Clusterin	0.048155478	1.472195821
Stk3	Serine/threonine-protein kinase 3	0.048187971	1.223949212
Trex1	RCG26064	0.048323503	1.205294705
Cd5l	CD5 antigen-like	0.048408537	1.621287665
Selenop	Selenoprotein P	0.048713829	1.35126957
Btk	Tyrosine-protein kinase	0.04880241	1.21118082
Smc2	Structural maintenance of chromosomes protein	0.048832745	1.250401653
Lrrfip1	Leucine-rich repeat flightless-interacting protein 1	0.048889546	1.214483764
Nckap1l	NCK associated protein 1 like (Predicted)	0.0489884	1.265395527
Fscn1	Fascin	0.048992407	0.73138098
Smarcd2	SWI/SNF-related matrix-associated actin-dependent regulator of chromatin subfamily D member 2	0.049128887	1.208092486

Supplemental Table 2. continued

Protein	Description	*P* value	Fold Change
Mug1	Murinoglobulin-1	0.049171274	1.244647404
Lbh	Protein LBH	0.049222684	1.270945723
Lbp	Lipopolysaccharide-binding protein	0.049498312	1.715342314

Supplemental Table 3. Sequences matched with CIRBP binding motif in the 3' UTR region of *Dhodh* mRNA

Strand	p-value	Matched Sequence
+	0.00307	CAAATCCCATTGTCACTCCCTAGATCTAAATCCTGGGATTGATCAGTATCA
+	0.005	CAGAAGGACATTGGCTTCTTGGGAGGAAAAATCGTGGAGAAAATAAAGCCA
+	0.00766	AGTGAAGAACTTCTCTAATCACTTGAGGAGACCACAAATCCCATTGTCACT
+	0.008	CCATTGGAGCAGATCATCGGAGGTGACCATACGTGCCAGAAGTCCCATCCA

Supplemental Table 4. Antibodies used in immunofluorescence staining.

Target antigen	Vendor	Catalog#	Concentration
Rabbit anti-Myeloperoxidase	Abcam	ab208670	1:200
Rabbit anti-COX2	Abcam	ab179800	1:200
Rabbit anti-CIRBP antibody	Proteintech	10209-2-AP	1:200
Rabbit anti-SP1 antibody	Proteintech	21962-1-AP	1:200
Mouse anti-Actinin	Abcam	ab9465	1:200
Rabbit anti-GSDMD	Proteintech	20770-1-AP	1:200
Rabbit anti-4 Hydroxynonenal	Bioss	bs-6313R	1:200
Mouse anti-Vimentin	Proteintech	60330-1-Ig	1:200

Supplemental Table 5. Antibodies used in western blot.

Target antigen	Vendor	Catalog#	Concentration
Rabbit anti-β-tubulin antibody	Abcam	ab179513	1:1000
Rabbit anti-Actin antibody	Abcam	ab185058	1:10000
Rabbit anti-β-actin antibody	Abcam	ab8227	1:5000
Rabbit anti-PTGS2 antibody	CST	12282S	1:1000
Rabbit anti-ACSL4 antibody	Abcam	ab155282	1:1000
Rabbit anti-CIRBP antibody (for Rat)	Proteintech	10209-2-AP	1:1000
Rabbit anti-CIRBP antibody (for Human)	Abcam	ab191885	1:1000
Rabbit anti-SP1 antibody	Proteintech	21962-1-AP	1:1000
Rabbit anti-DHODH antibody	Proteintech	14877-1-AP	1:1000
Rabbit anti-Histone H3 antibody	Abcam	ab176842	1:1000
Rabbit anti-Caspase 1 antibody	Proteintech	22915-1-AP	1:1000
Rabbit anti-Caspase 3 antibody	CST	9662S	1:1000
Rabbit anti-Cleaved caspase 3 antibody	CST	9664T	1:1000
Rabbit anti-NOX2 antibody	Abcam	ab129068	1:1000
Rabbit anti-ALOX5 antibody	Abcam	ab169755	1:1000
Rabbit anti-ALOX15 antibody	Abcam	ab244205	1:1000
Rabbit anti-GPX4 antibody	Abcam	ab125066	1:1000
