# Supplementary material for: Cirbp suppression compromises DHODH-mediated ferroptosis defense and attenuates hypothermic cardioprotection in an aged donor transplantation model
Source: J Clin Invest. 2024 Mar 12;134(9):e175645. doi: 10.1172/JCI175645 (PMC11060748; doi:10.1172/JCI175645)
Supplement: Unedited blot and gel images [file jci-134-175645-s037.pdf]

Figure 2E

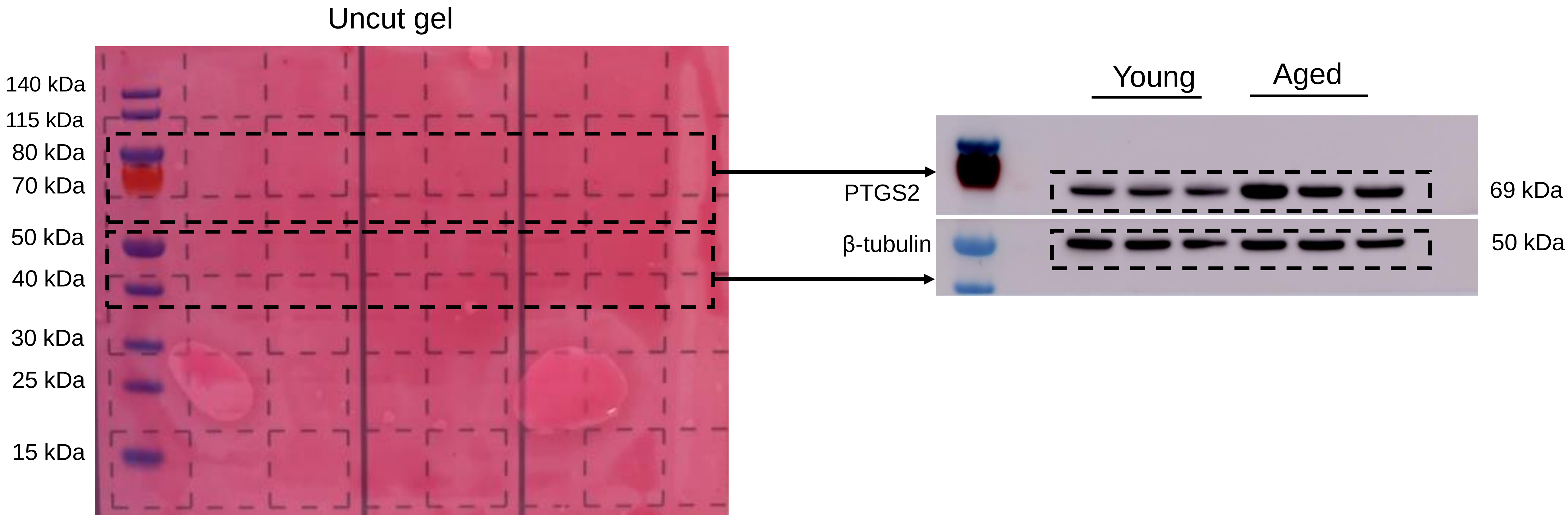

Uncut gel
140 kDa
115 kDa
80 kDa
70 kDa
50 kDa
40 kDa
30 kDa
25 kDa
15 kDa
Young
Aged
PTGS2
β-tubulin
69 kDa
50 kDa

Figure 3J

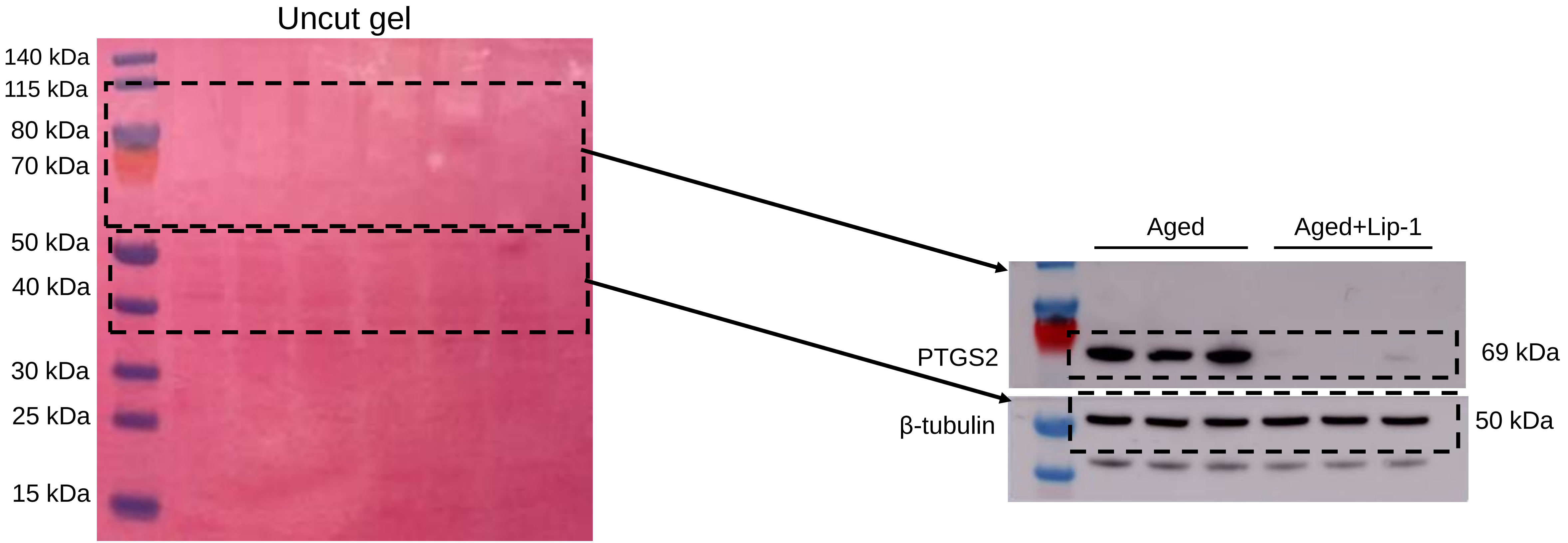

Uncut gel
140 kDa
115 kDa
80 kDa
70 kDa
50 kDa
40 kDa
30 kDa
25 kDa
15 kDa
Aged
Aged+Lip-1
PTGS2
β-tubulin
69 kDa
50 kDa

Figure 4B

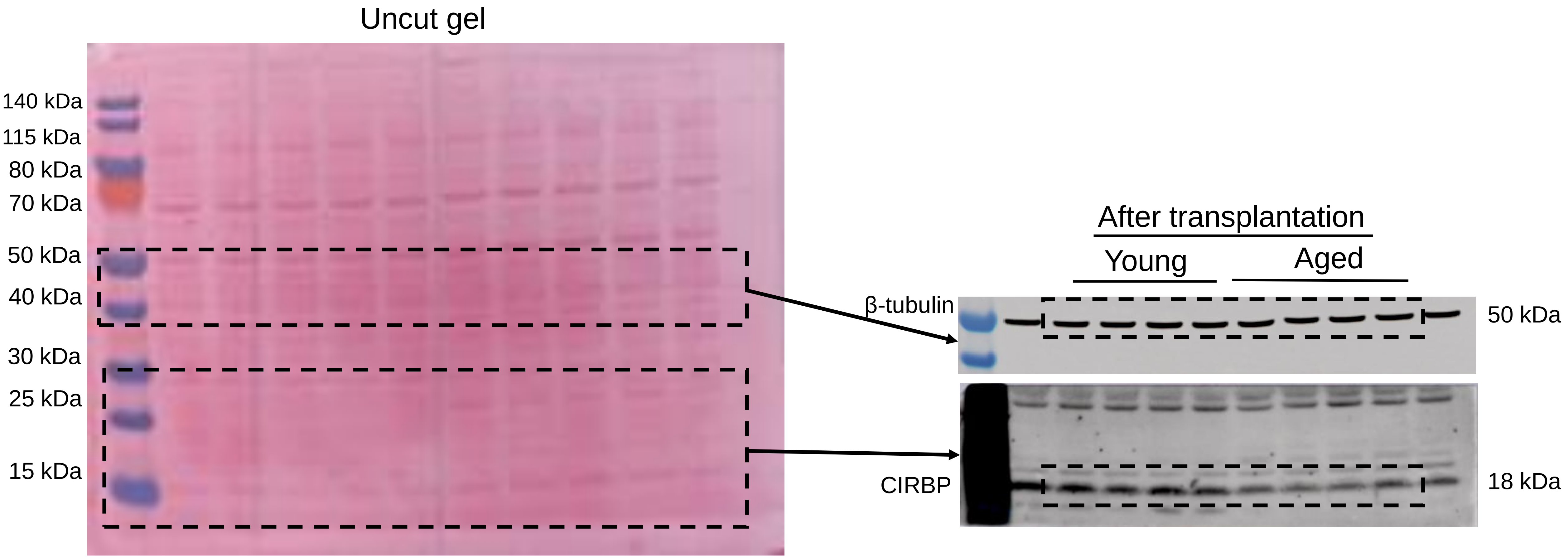
Uncut gel
140 kDa
115 kDa
80 kDa
70 kDa
50 kDa
40 kDa
30 kDa
25 kDa
15 kDa
After transplantation
Young
Aged
β-tubulin
50 kDa
CIRBP
18 kDa

Figure 4D

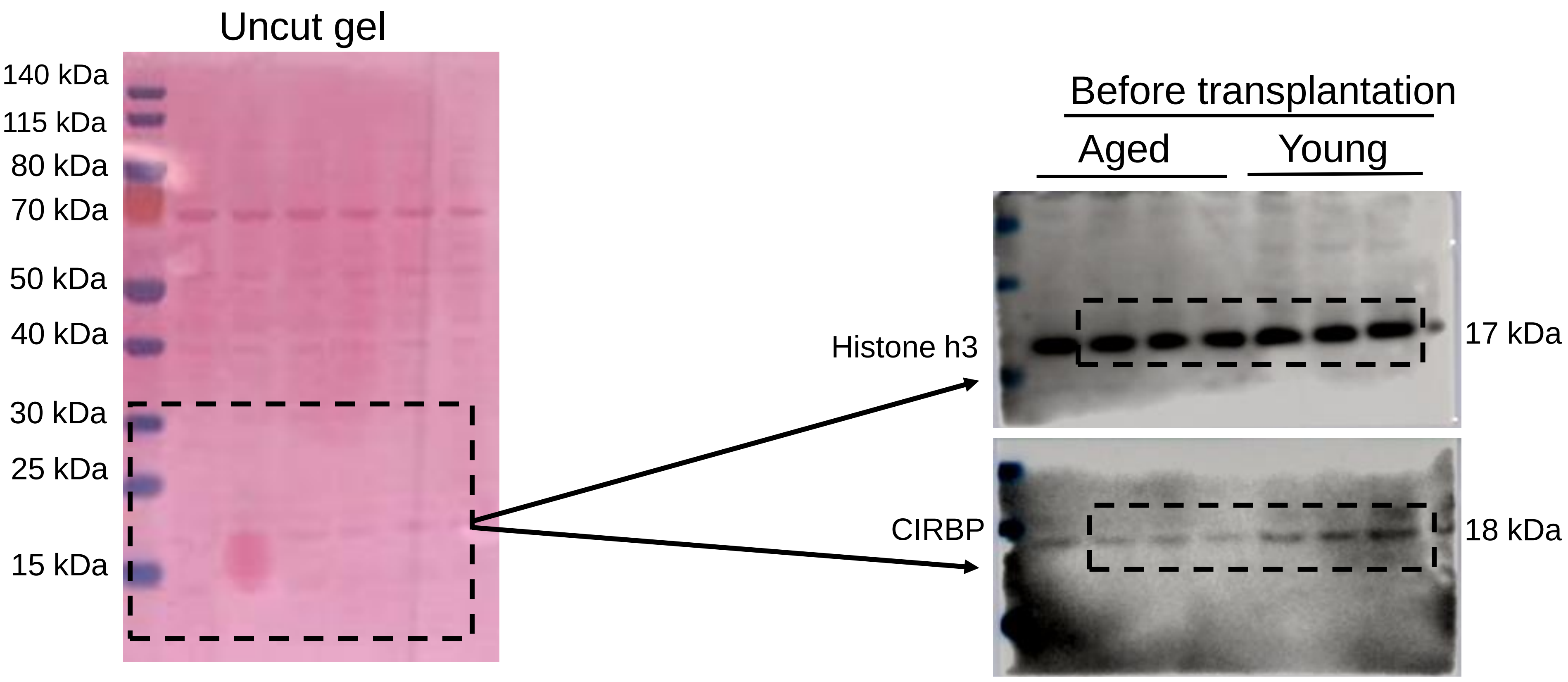
Uncut gel
140 kDa
115 kDa
80 kDa
70 kDa
50 kDa
40 kDa
30 kDa
25 kDa
15 kDa
Before transplantation
Aged
Young
Histone h3
17 kDa
CIRBP
18 kDa

Figure 4F

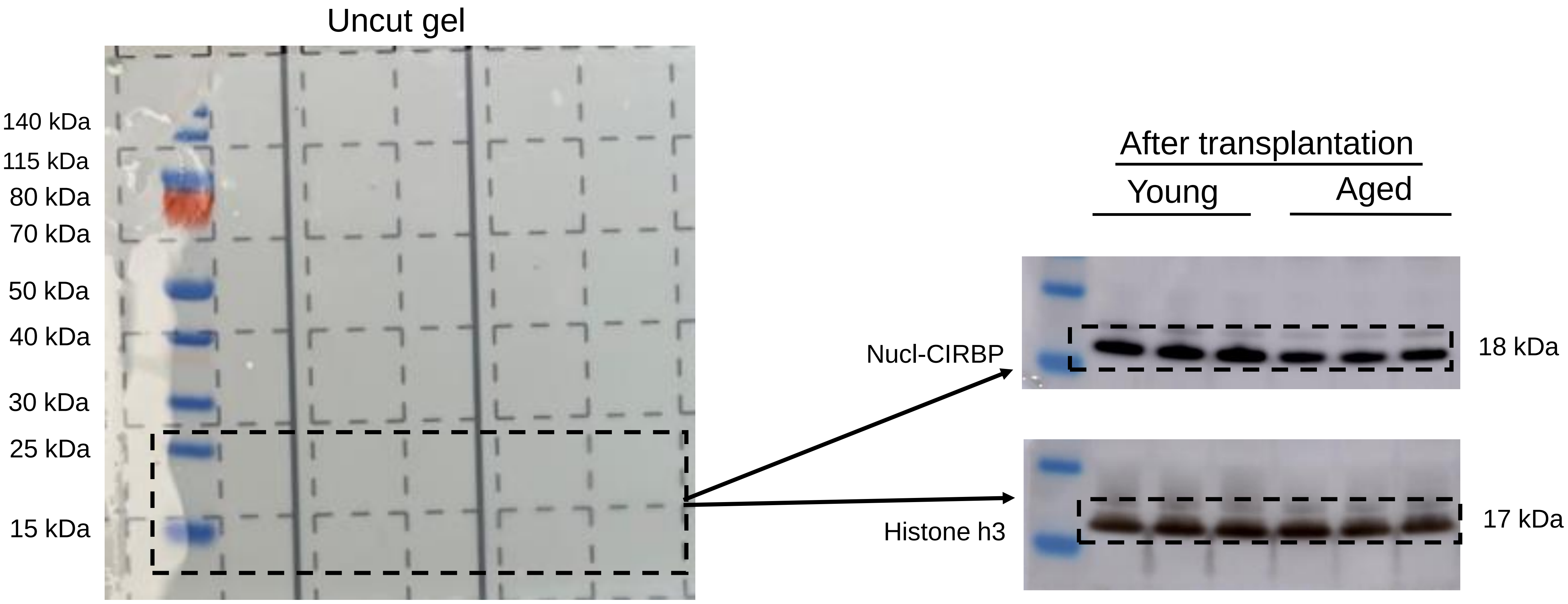

Uncut gel
140 kDa
115 kDa
80 kDa
70 kDa
50 kDa
40 kDa
30 kDa
25 kDa
15 kDa
After transplantation
Young
Aged
Nucl-CIRBP
18 kDa
Histone h3
17 kDa

Figure 4G

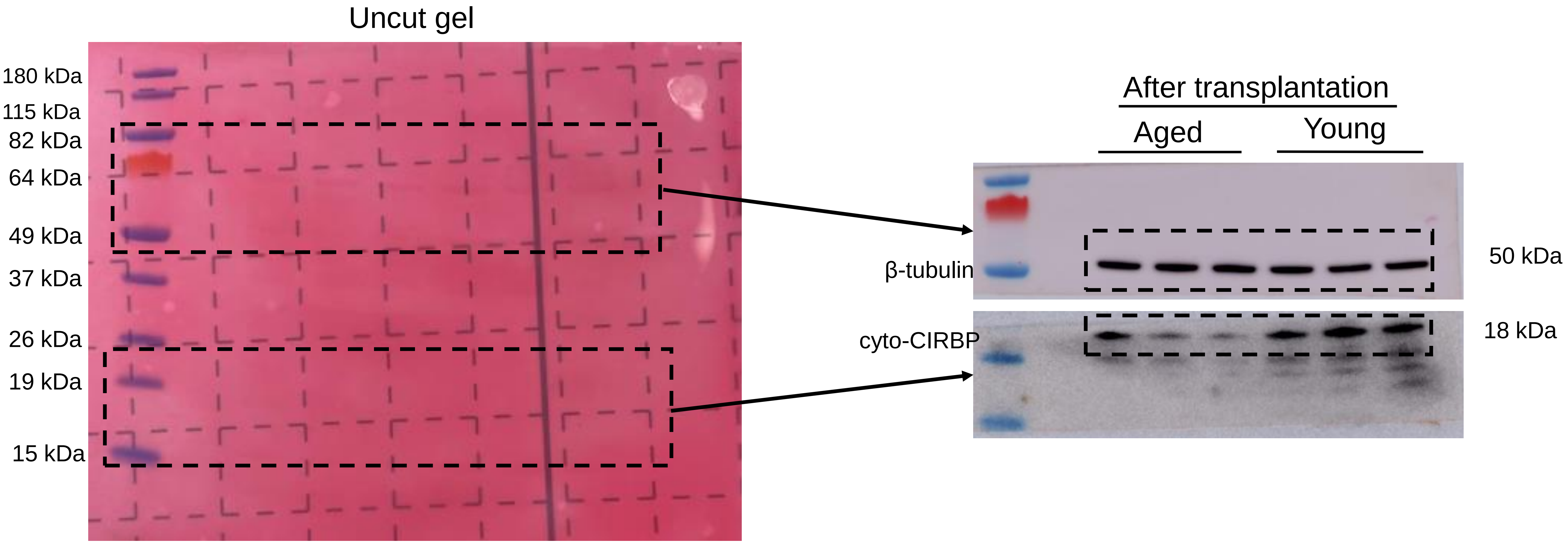
Uncut gel
180 kDa
115 kDa
82 kDa
64 kDa
49 kDa
37 kDa
26 kDa
19 kDa
15 kDa
After transplantation
Aged
Young
β-tubulin
cyto-CIRBP
50 kDa
18 kDa

Figure 4I

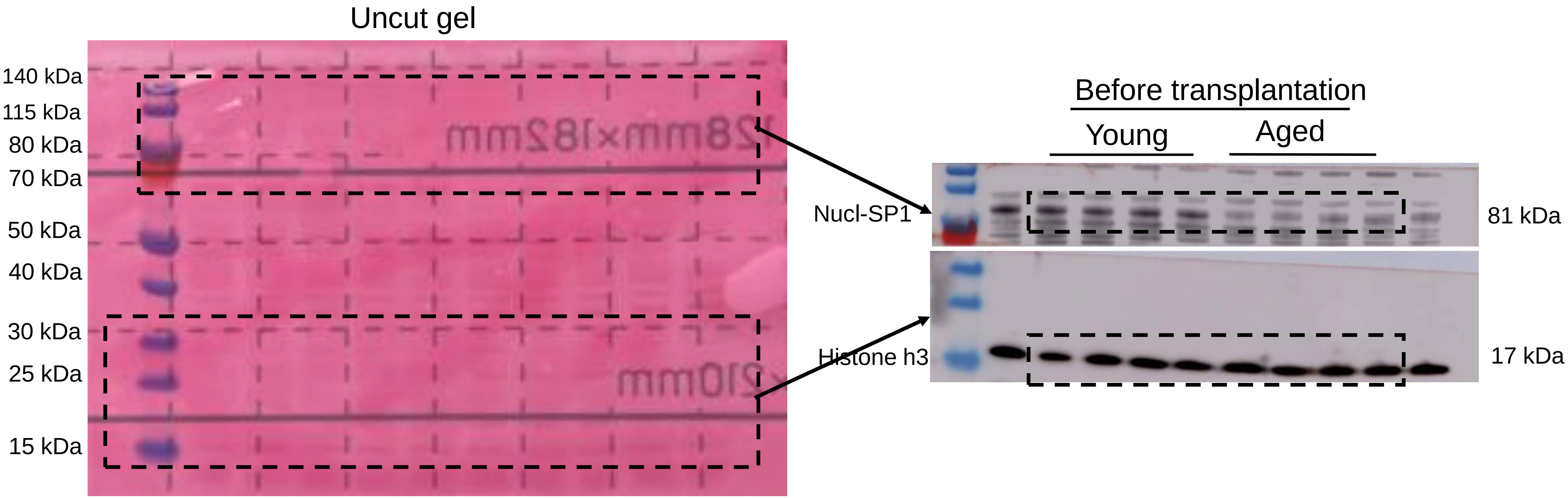

Uncut gel
140 kDa
115 kDa
80 kDa
70 kDa
50 kDa
40 kDa
30 kDa
25 kDa
15 kDa
Before transplantation
Young
Aged
Nucl-SP1
Histone h3
81 kDa
17 kDa

Figure 5B

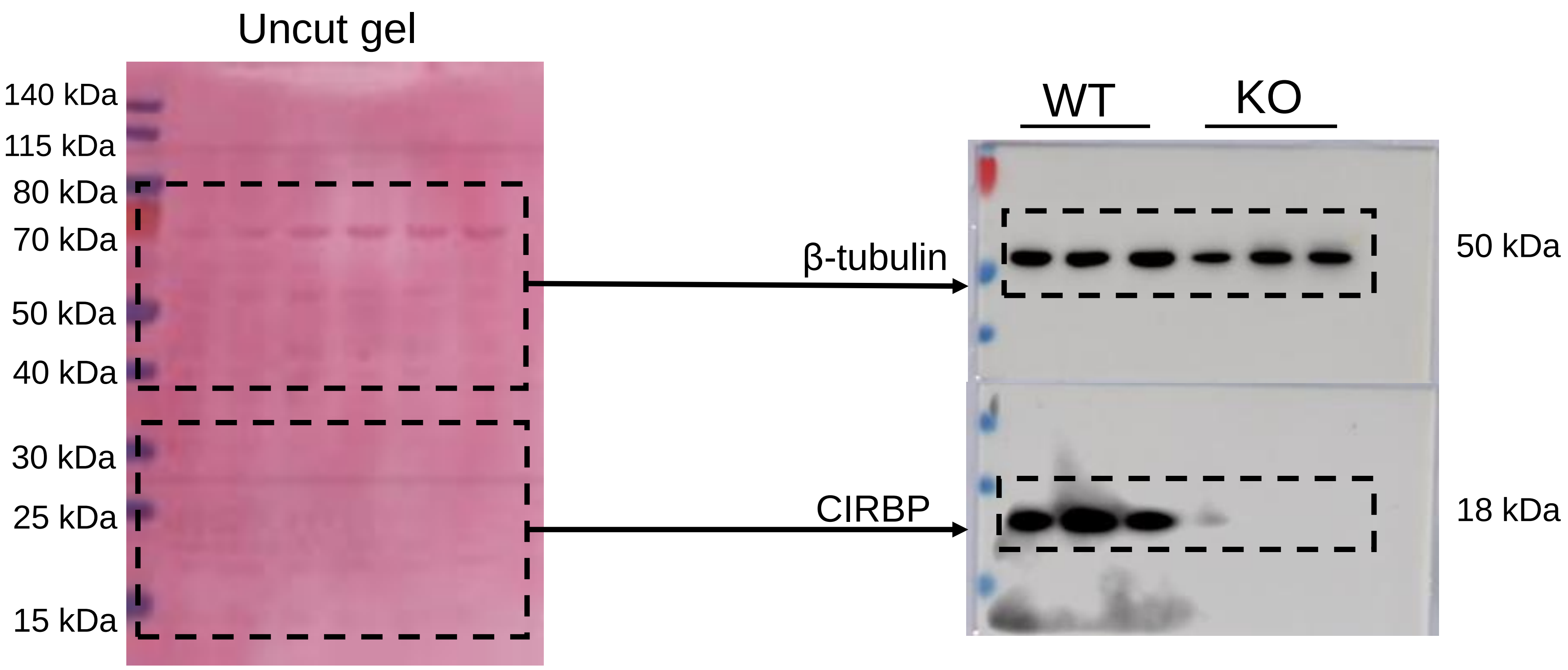
Uncut gel
140 kDa
115 kDa
80 kDa
70 kDa
50 kDa
40 kDa
30 kDa
25 kDa
15 kDa
β-tubulin
CIRBP
WT
KO
50 kDa
18 kDa

Figure 5L

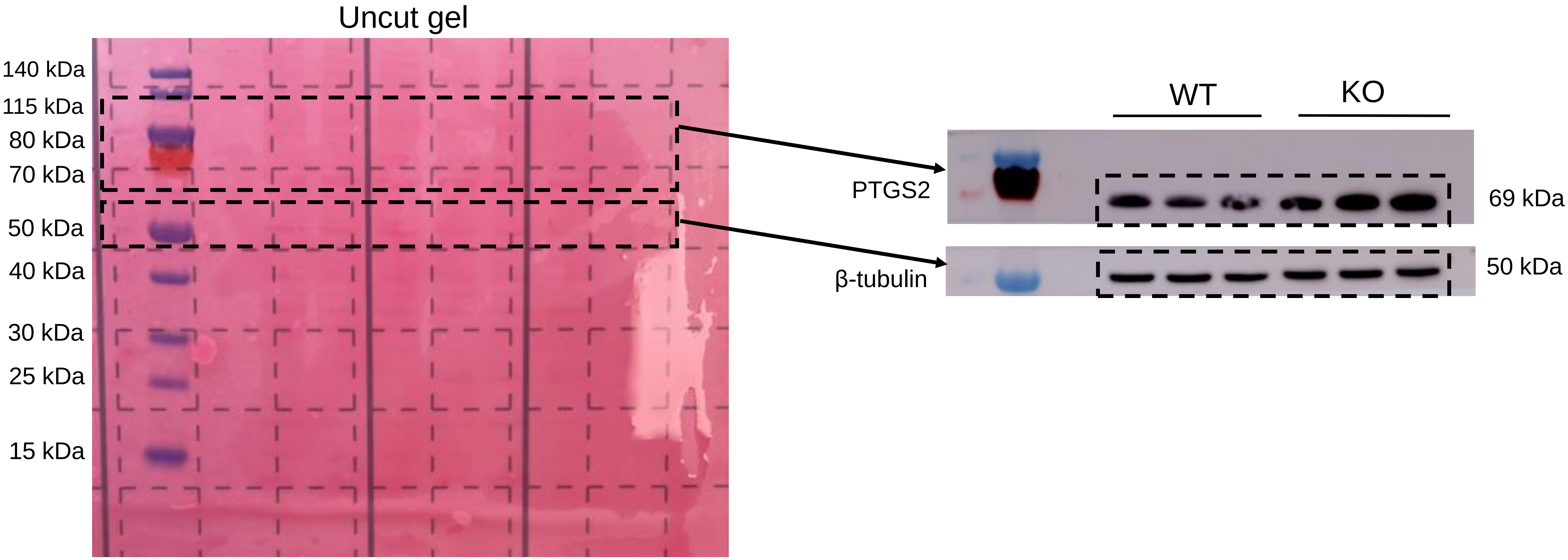
Uncut gel
140 kDa
115 kDa
80 kDa
70 kDa
50 kDa
40 kDa
30 kDa
25 kDa
15 kDa
WT
KO
PTGS2
β-tubulin
69 kDa
50 kDa

Figure 6E

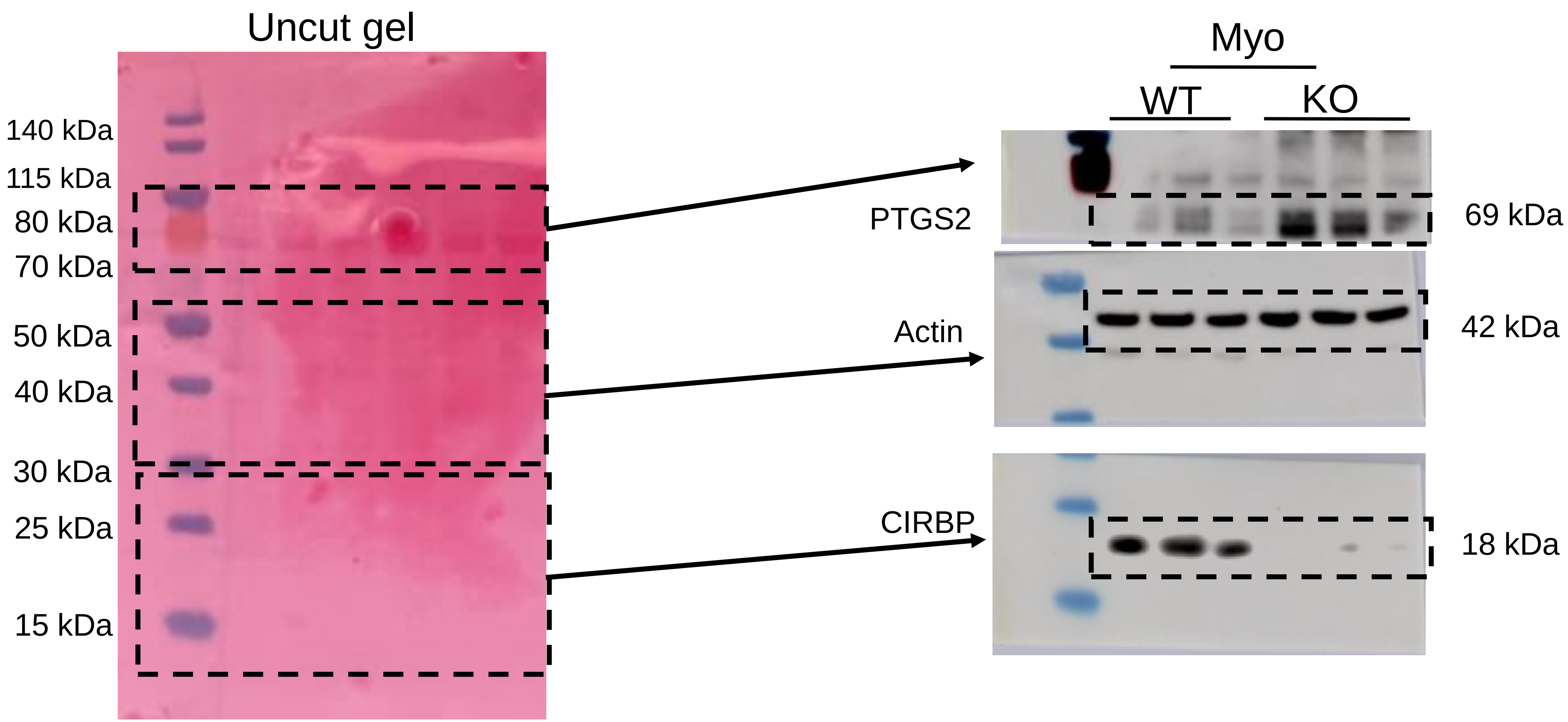

Uncut gel
140 kDa
115 kDa
80 kDa
70 kDa
50 kDa
40 kDa
30 kDa
25 kDa
15 kDa
Myo
WT
KO
PTGS2
Actin
CIRBP
69 kDa
42 kDa
18 kDa

Figure 6E

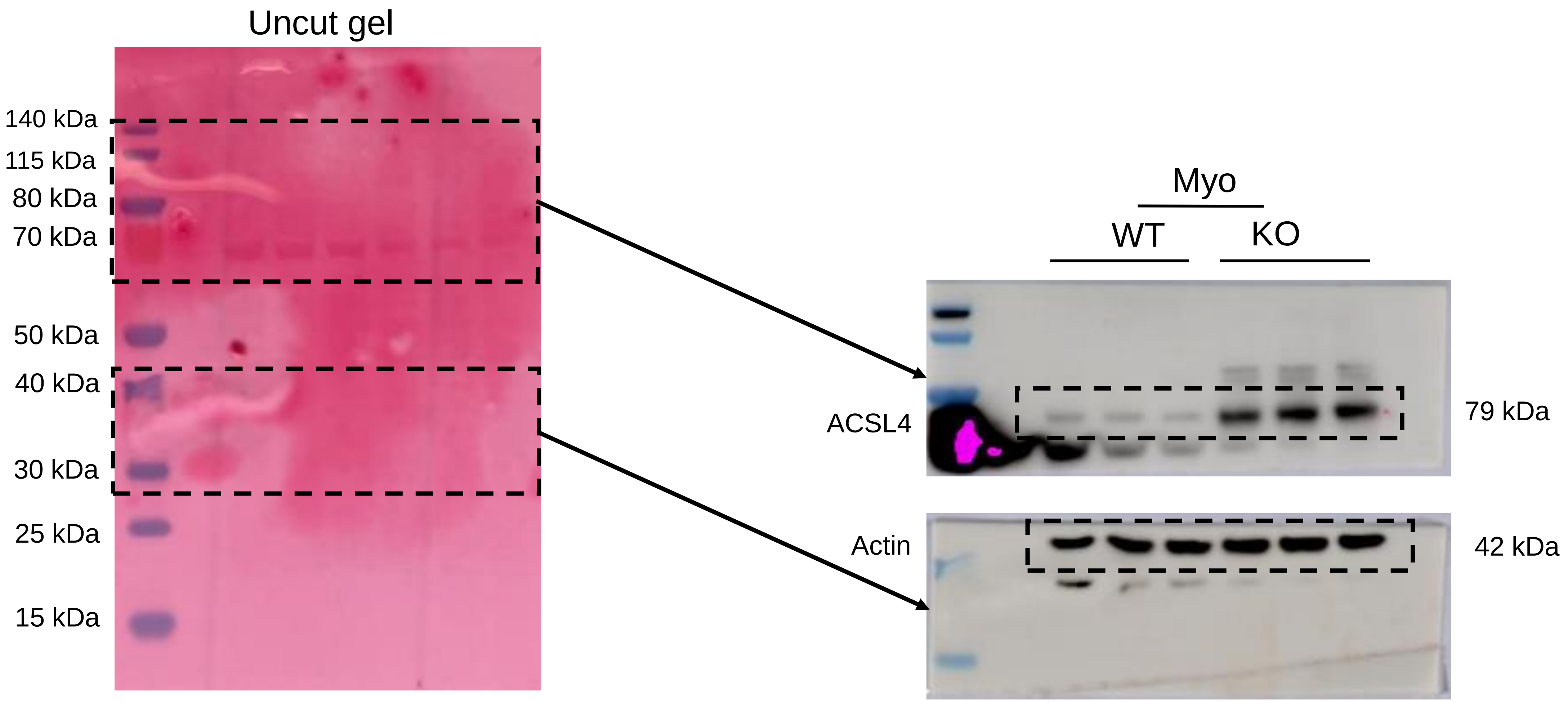

Uncut gel
140 kDa
115 kDa
80 kDa
70 kDa
50 kDa
40 kDa
30 kDa
25 kDa
15 kDa
Myo
WT
KO
ACSL4
79 kDa
Actin
42 kDa

Figure 6M

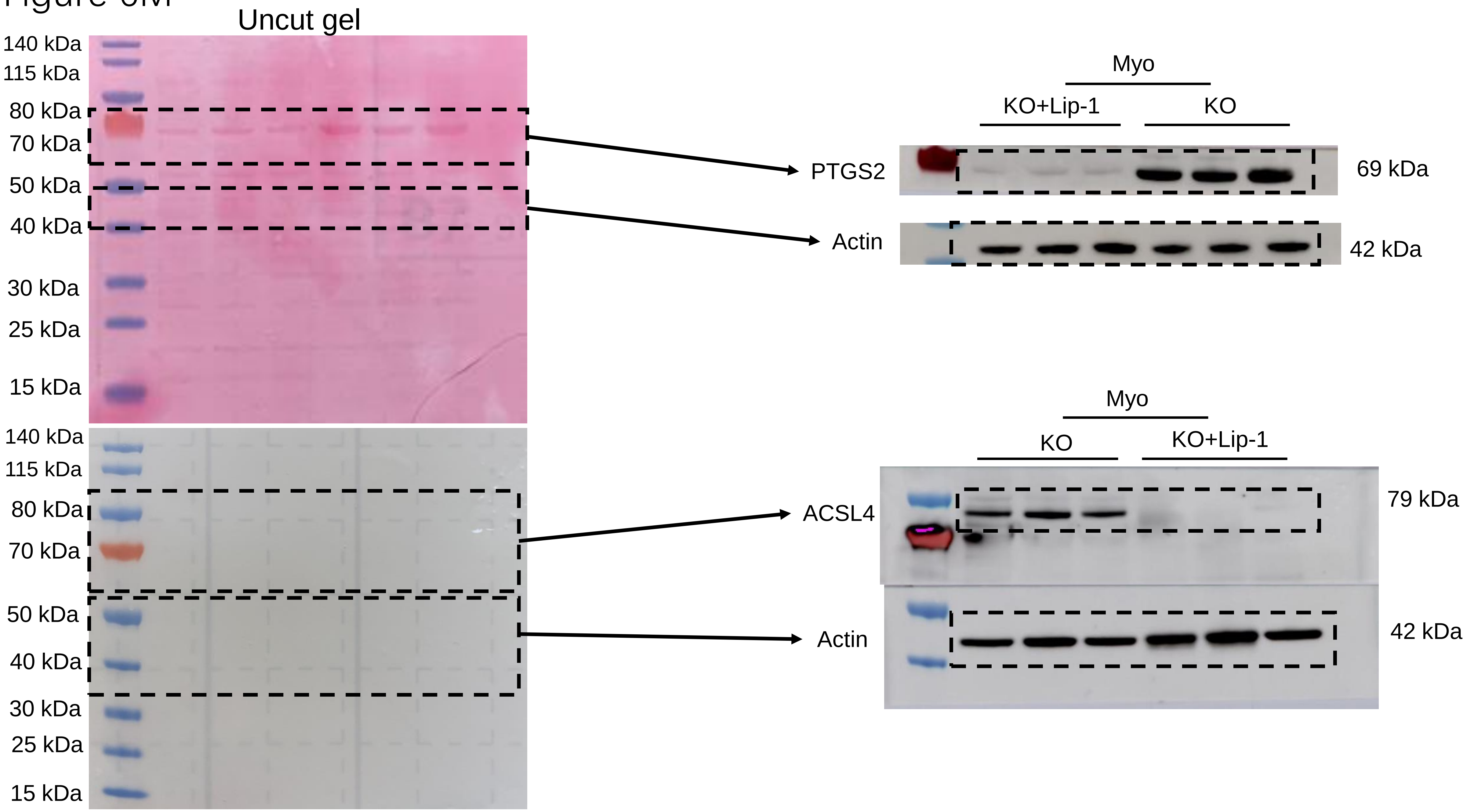

Uncut gel
140 kDa
115 kDa
80 kDa
70 kDa
50 kDa
40 kDa
30 kDa
25 kDa
15 kDa
Myo
KO+Lip-1
KO
PTGS2
69 kDa
Actin
42 kDa
140 kDa
115 kDa
80 kDa
70 kDa
50 kDa
40 kDa
30 kDa
25 kDa
15 kDa
Myo
KO
KO+Lip-1
ACSL4
79 kDa
Actin
42 kDa

Figure 7K

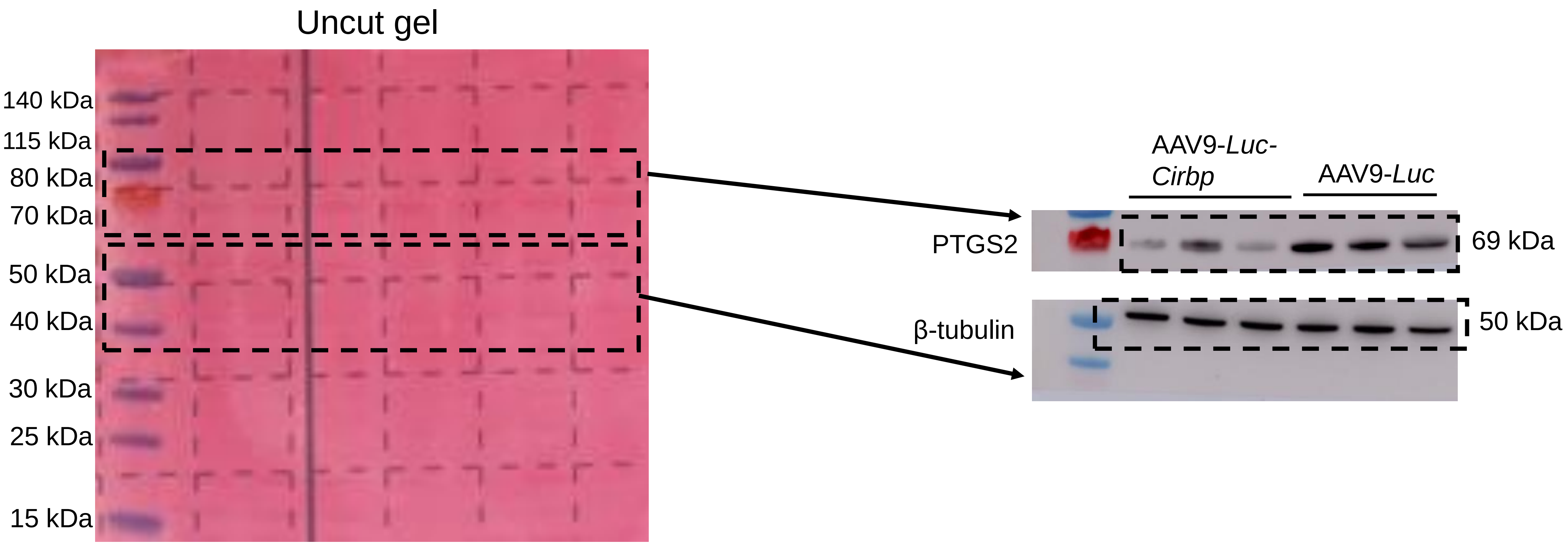
Uncut gel
140 kDa
115 kDa
80 kDa
70 kDa
50 kDa
40 kDa
30 kDa
25 kDa
15 kDa
AAV9-*Luc*-*Cirbp*
AAV9-*Luc*
PTGS2
69 kDa
β-tubulin
50 kDa

Figure 8C

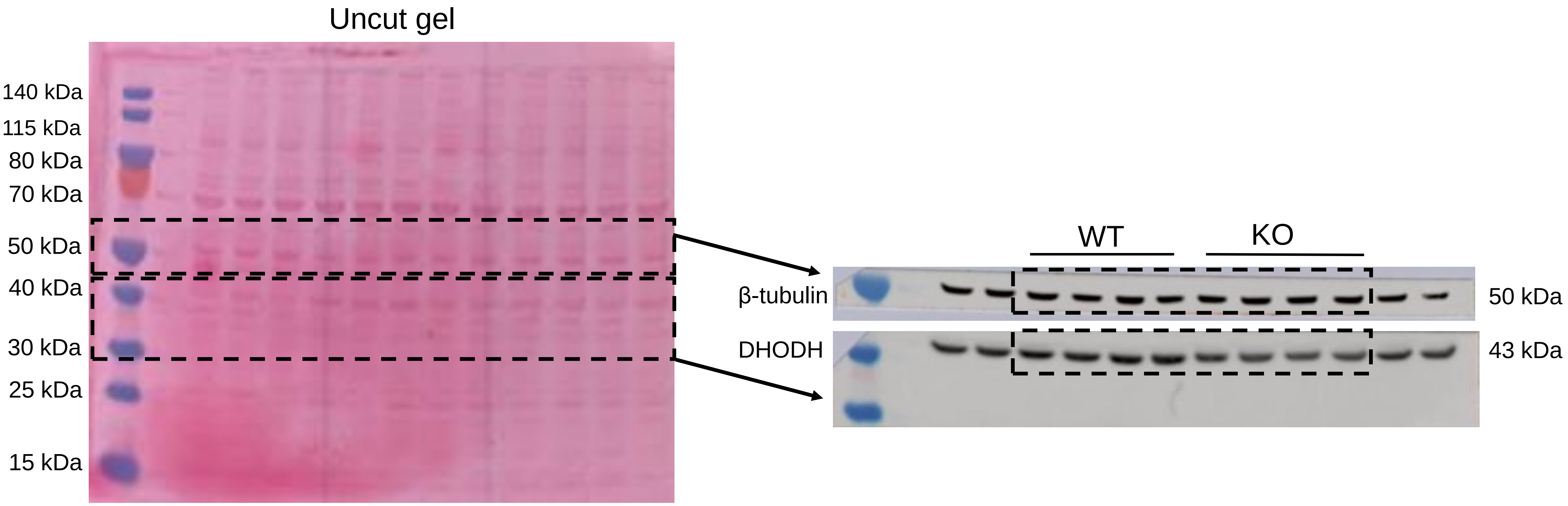

Uncut gel
140 kDa
115 kDa
80 kDa
70 kDa
50 kDa
40 kDa
30 kDa
25 kDa
15 kDa
WT
KO
β-tubulin
DHODH
50 kDa
43 kDa

Figure 8C

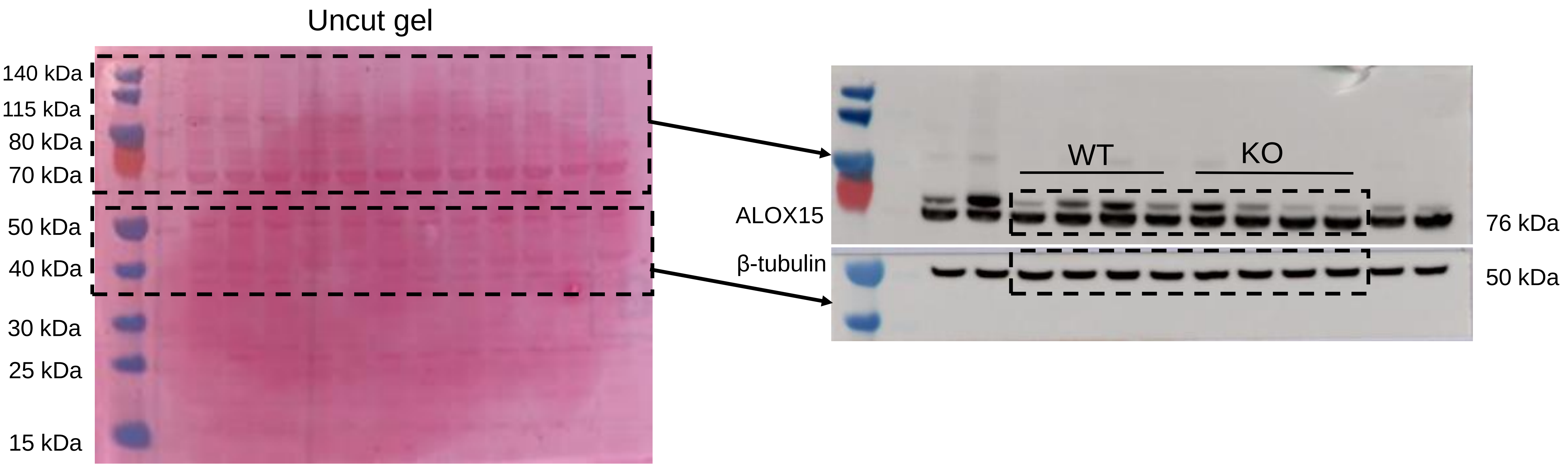
Uncut gel
140 kDa
115 kDa
80 kDa
70 kDa
50 kDa
40 kDa
30 kDa
25 kDa
15 kDa
WT
KO
ALOX15
β-tubulin
76 kDa
50 kDa

Figure 8C

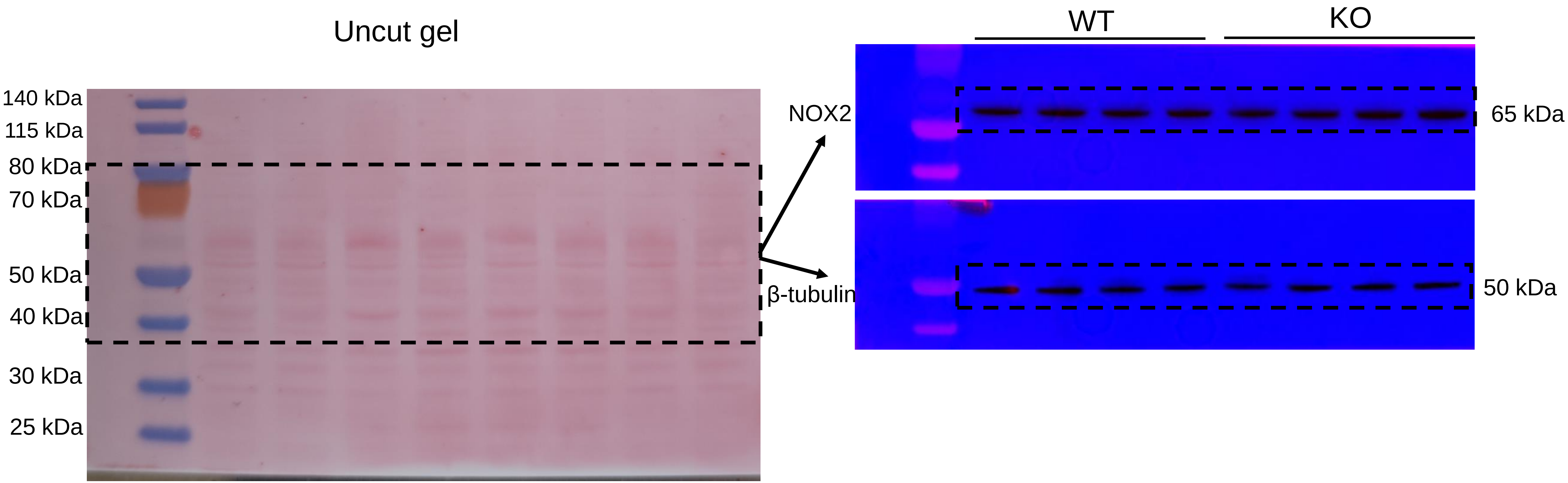

Uncut gel
140 kDa
115 kDa
80 kDa
70 kDa
50 kDa
40 kDa
30 kDa
25 kDa
NOX2
β-tubulin
WT
KO
65 kDa
50 kDa

Figure 8C

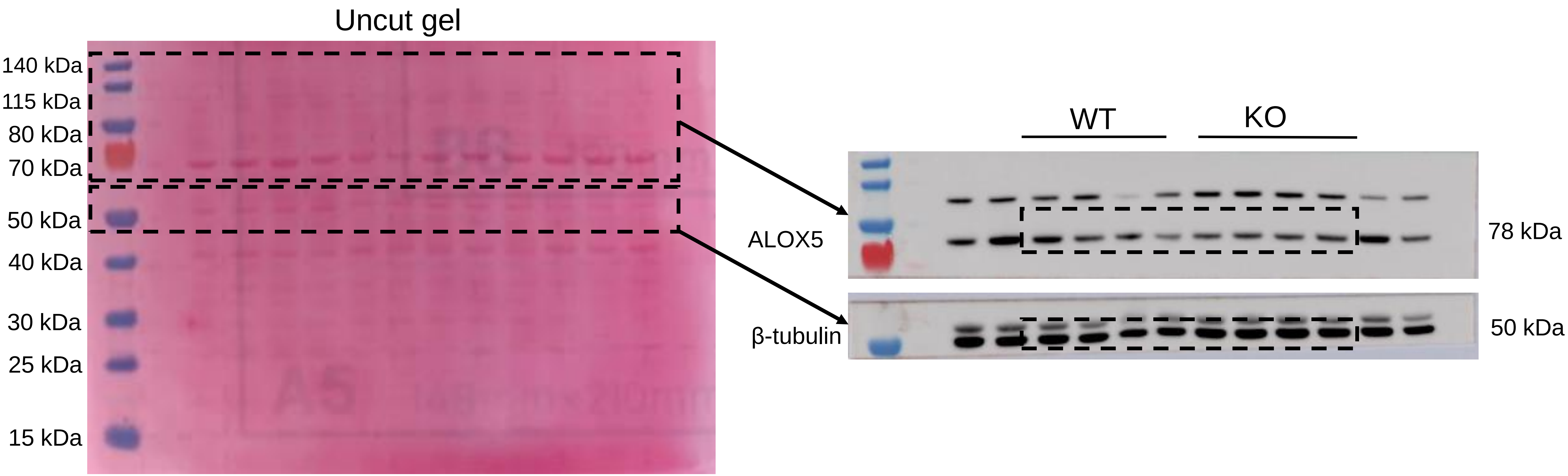

Uncut gel
140 kDa
115 kDa
80 kDa
70 kDa
50 kDa
40 kDa
30 kDa
25 kDa
15 kDa
WT
KO
ALOX5
β-tubulin
78 kDa
50 kDa

Figure 8F

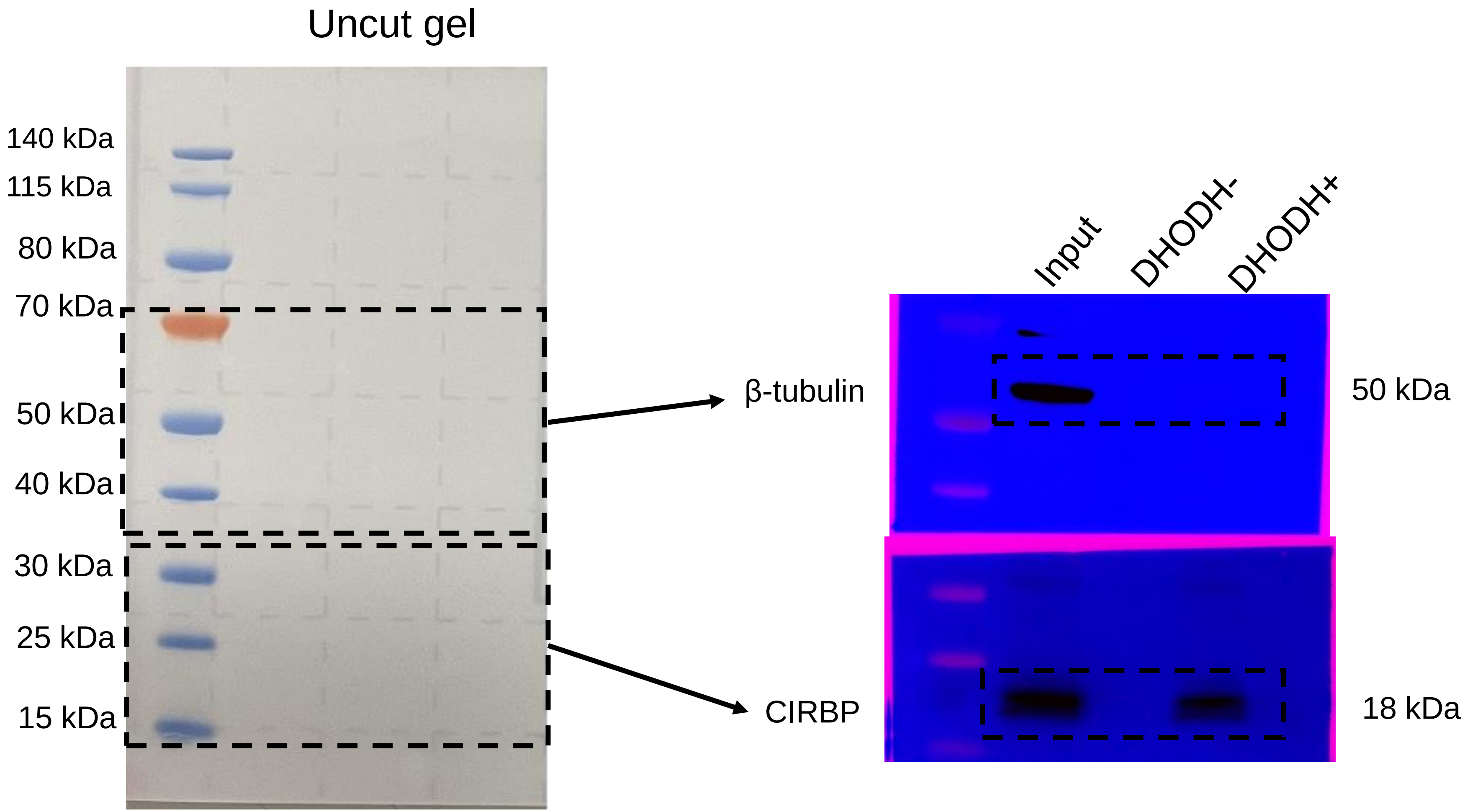

Uncut gel
140 kDa
115 kDa
80 kDa
70 kDa
50 kDa
40 kDa
30 kDa
25 kDa
15 kDa
β-tubulin
CIRBP
Input
DHODH-
DHODH+
50 kDa
18 kDa

Figure 8I

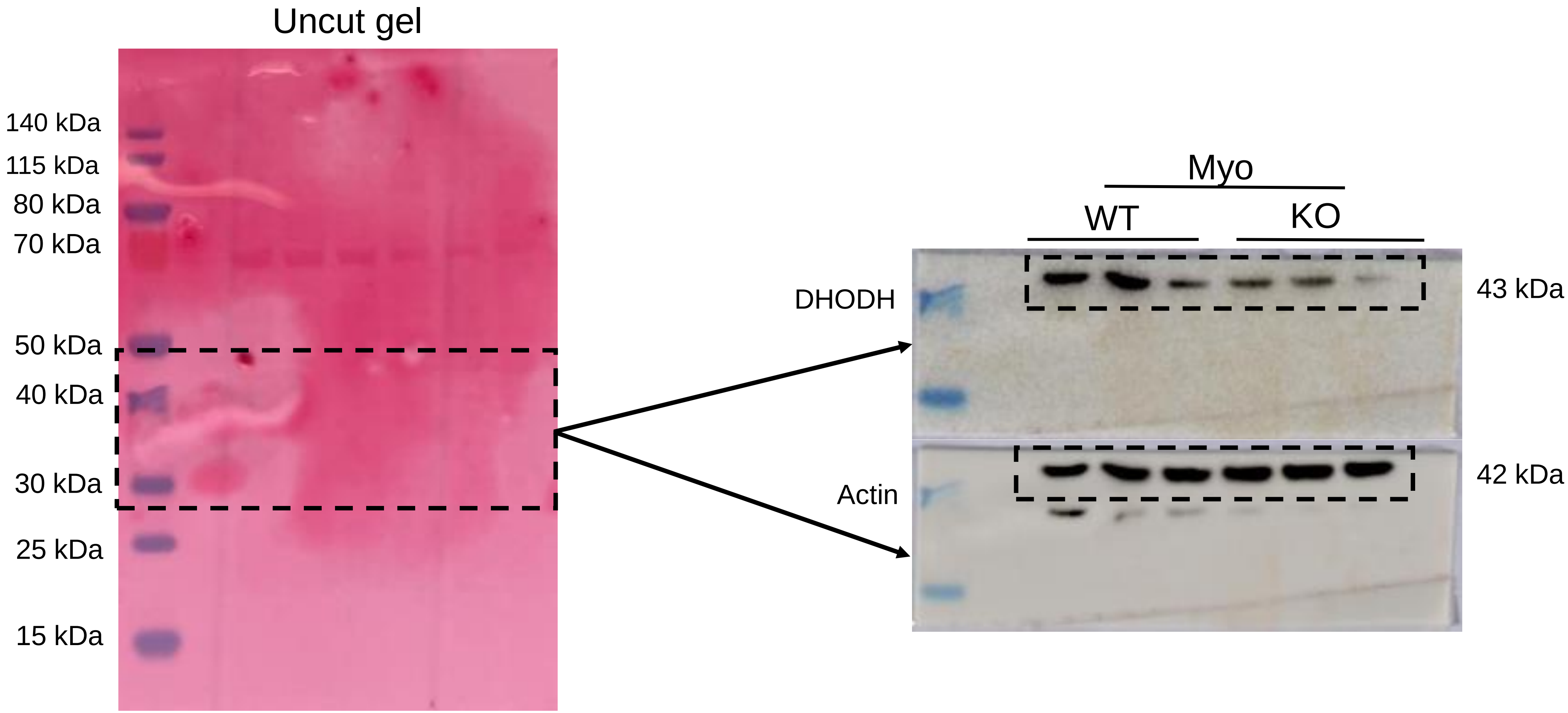
Uncut gel
140 kDa
115 kDa
80 kDa
70 kDa
50 kDa
40 kDa
30 kDa
25 kDa
15 kDa
Myo
WT
KO
DHODH
43 kDa
Actin
42 kDa

Figure 8K

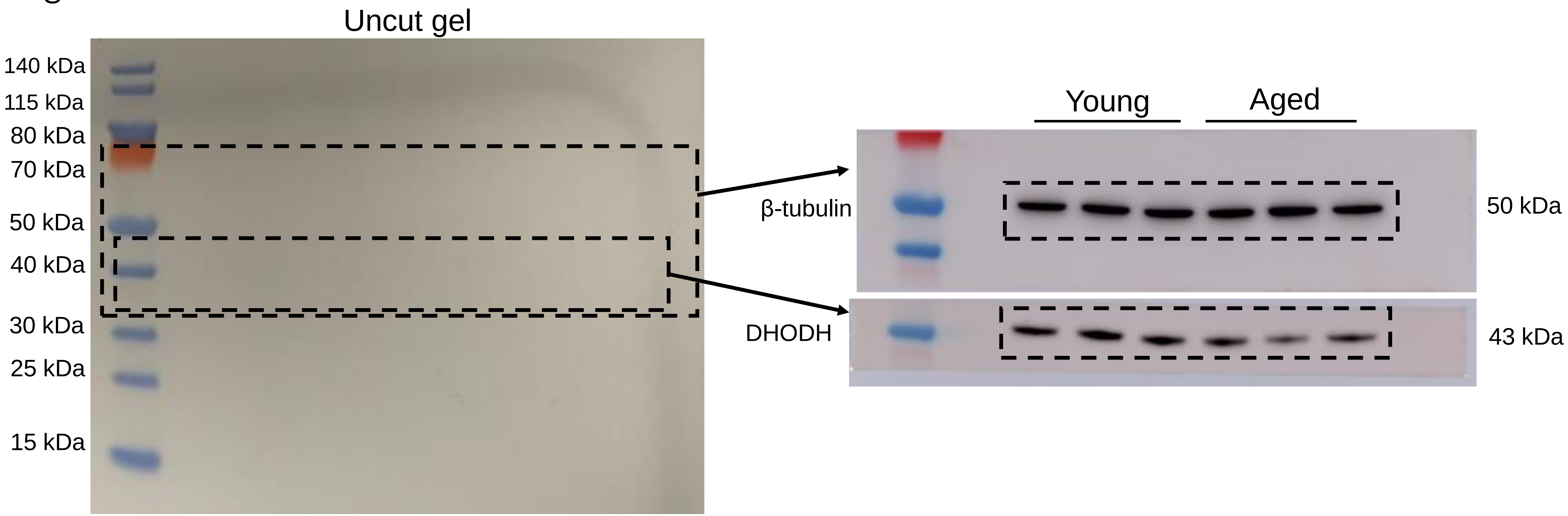

Uncut gel
140 kDa
115 kDa
80 kDa
70 kDa
50 kDa
40 kDa
30 kDa
25 kDa
15 kDa
Young
Aged
β-tubulin
50 kDa
DHODH
43 kDa

Figure 9B

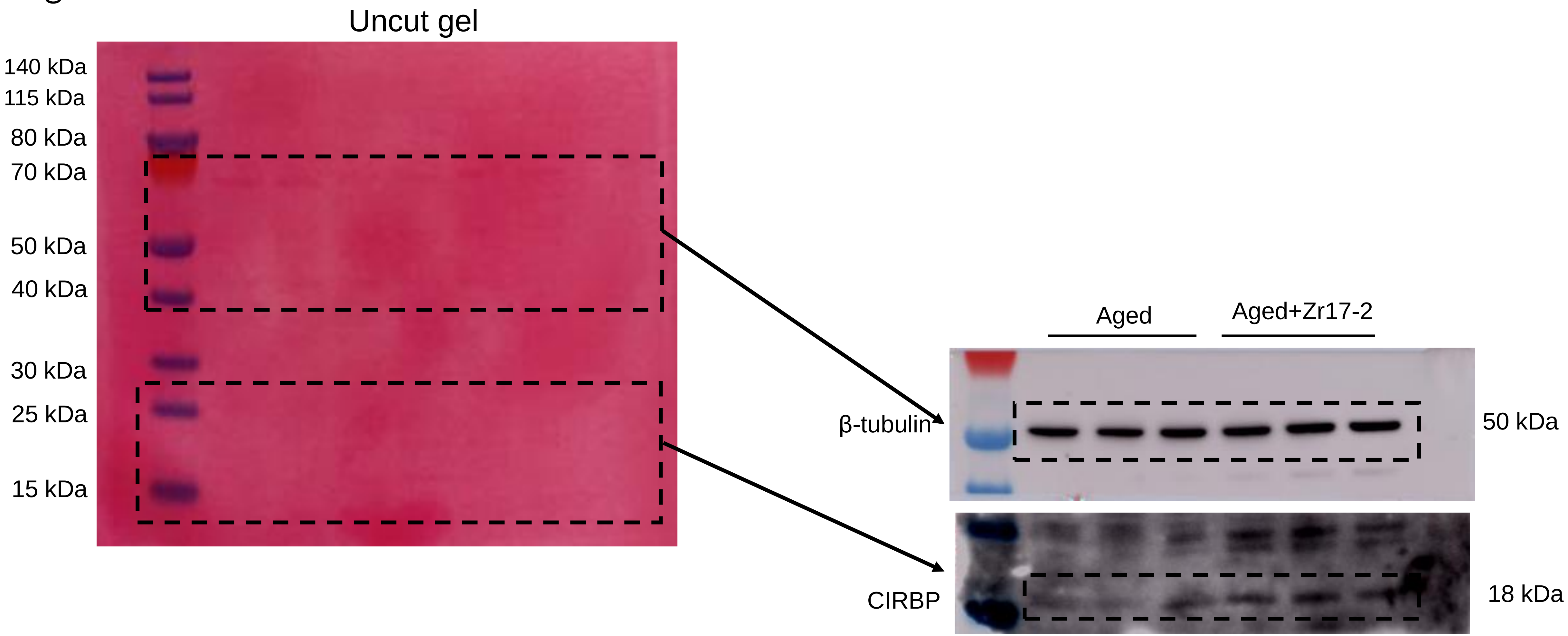

Uncut gel
140 kDa
115 kDa
80 kDa
70 kDa
50 kDa
40 kDa
30 kDa
25 kDa
15 kDa
Aged
Aged+Zr17-2
β-tubulin
50 kDa
CIRBP
18 kDa

Figure 9I

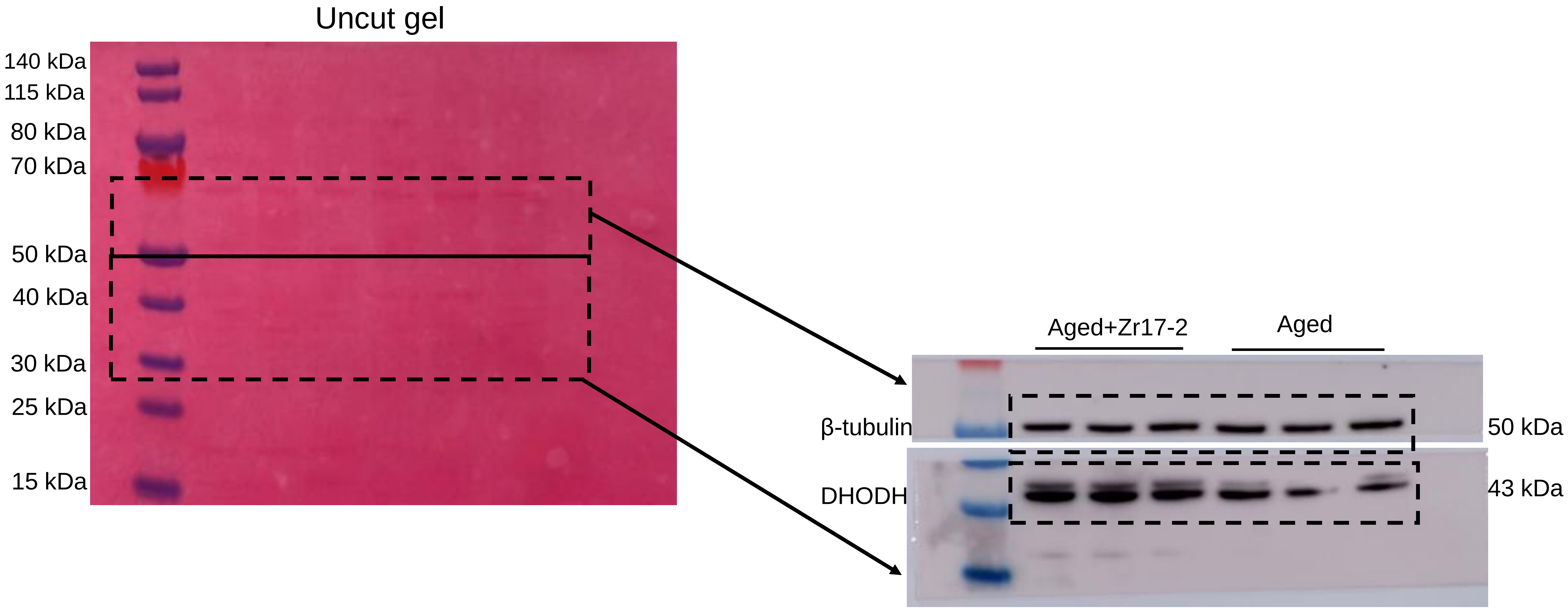
Uncut gel
140 kDa
115 kDa
80 kDa
70 kDa
50 kDa
40 kDa
30 kDa
25 kDa
15 kDa
Aged+Zr17-2
Aged
β-tubulin
DHODH
50 kDa
43 kDa

Figure 9N

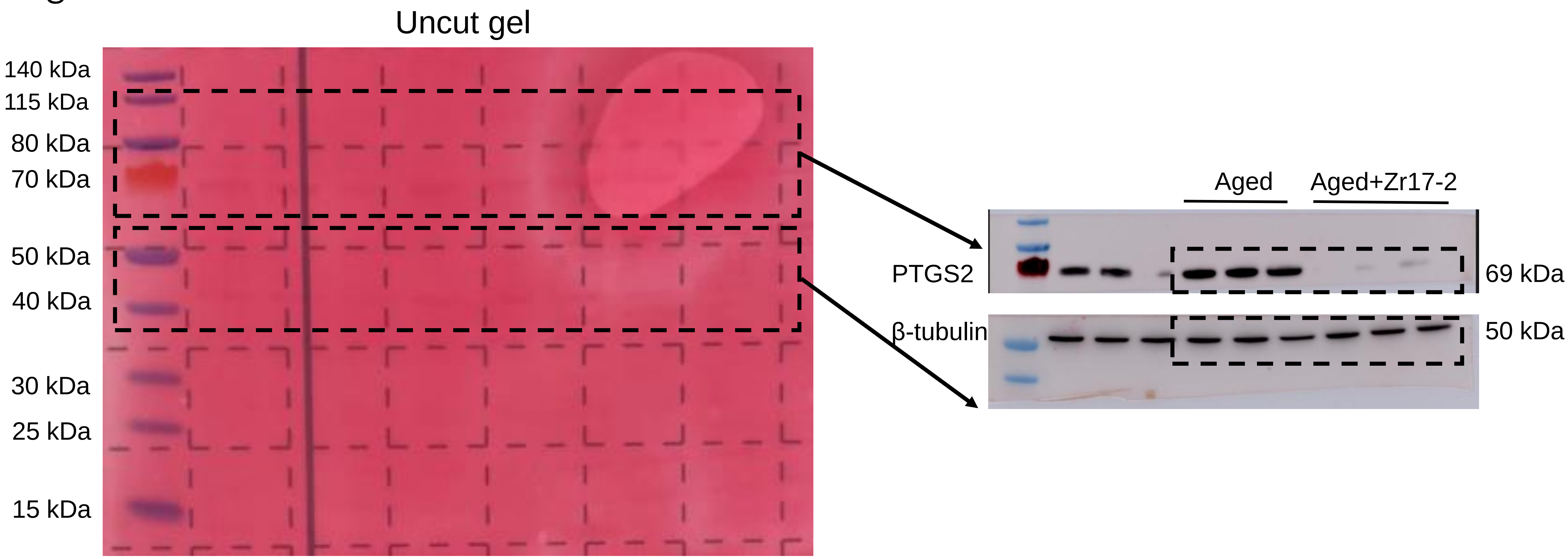
Uncut gel
140 kDa
115 kDa
80 kDa
70 kDa
50 kDa
40 kDa
30 kDa
25 kDa
15 kDa
Aged
Aged+Zr17-2
PTGS2
69 kDa
β-tubulin
50 kDa

Figure S6B

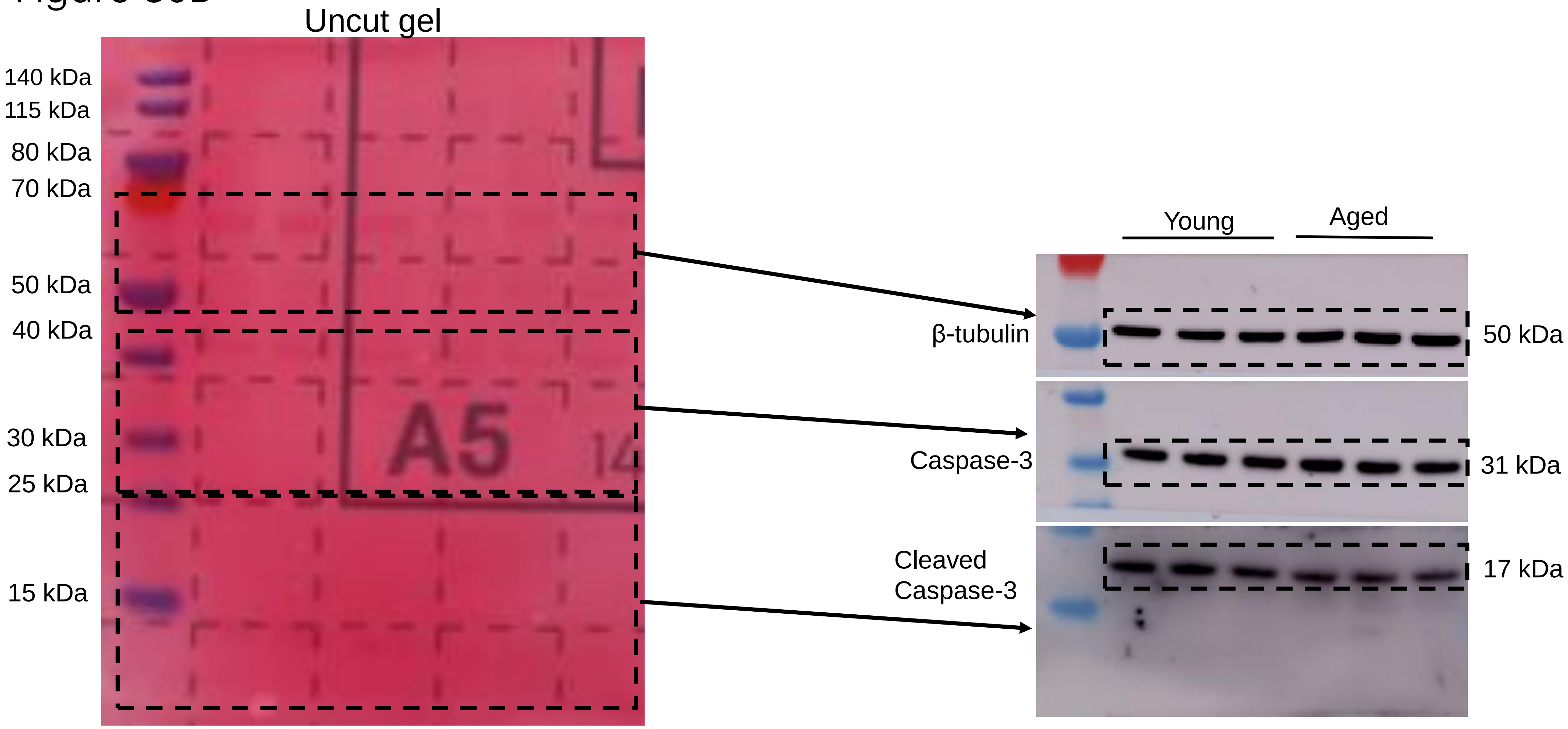
Uncut gel
140 kDa
115 kDa
80 kDa
70 kDa
50 kDa
40 kDa
30 kDa
25 kDa
15 kDa
A5
Young
Aged
β-tubulin
50 kDa
Caspase-3
31 kDa
Cleaved
Caspase-3
17 kDa

Figure S6D

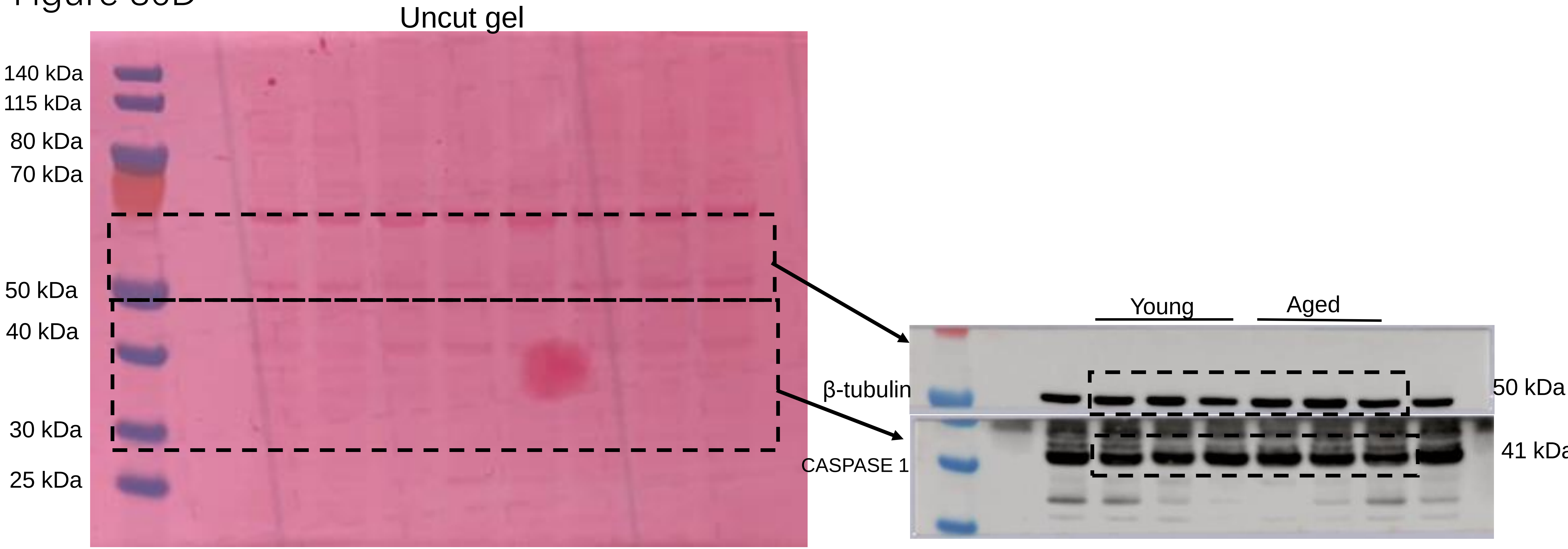

Uncut gel
140 kDa
115 kDa
80 kDa
70 kDa
50 kDa
40 kDa
30 kDa
25 kDa
Young
Aged
β-tubulin
CASPASE 1
50 kDa
41 kDa

Figure S7J

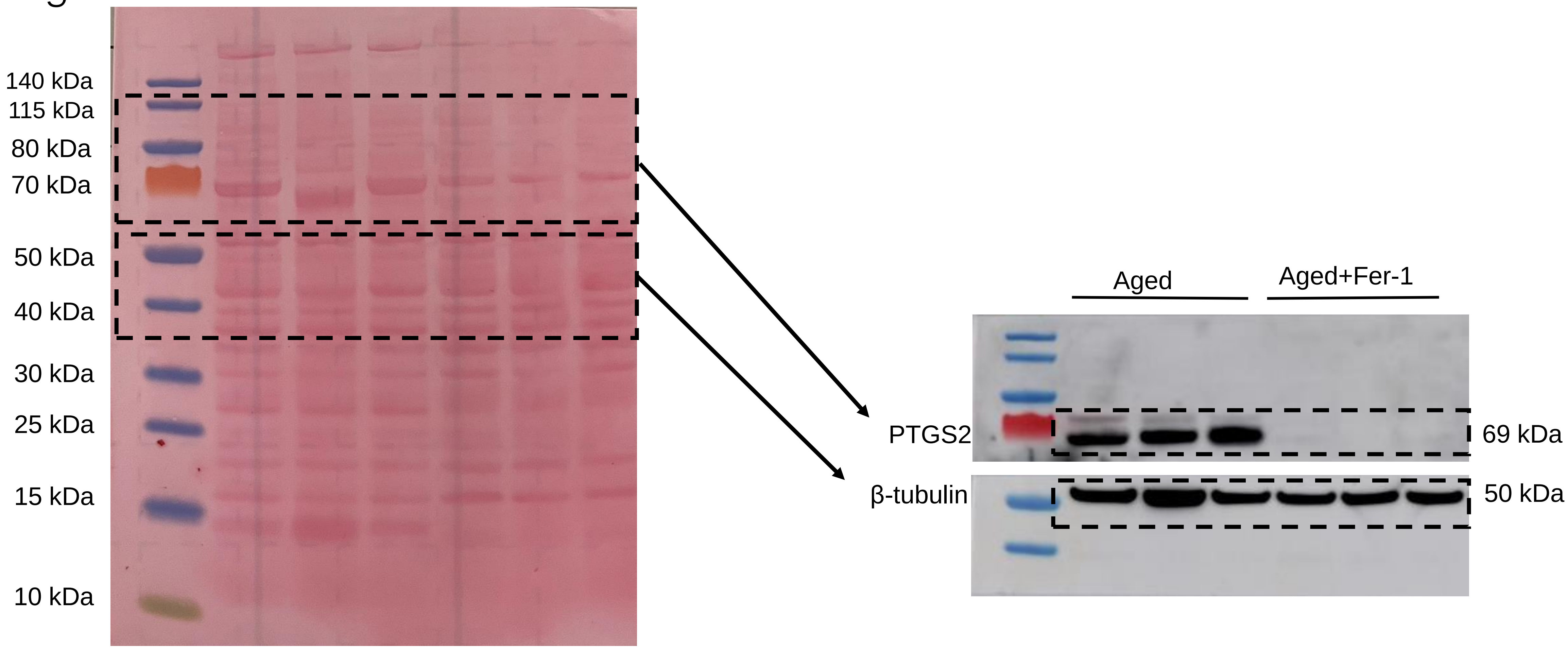
140 kDa
115 kDa
80 kDa
70 kDa
50 kDa
40 kDa
30 kDa
25 kDa
15 kDa
10 kDa
Aged
Aged+Fer-1
PTGS2
69 kDa
β-tubulin
50 kDa

Figure S8B

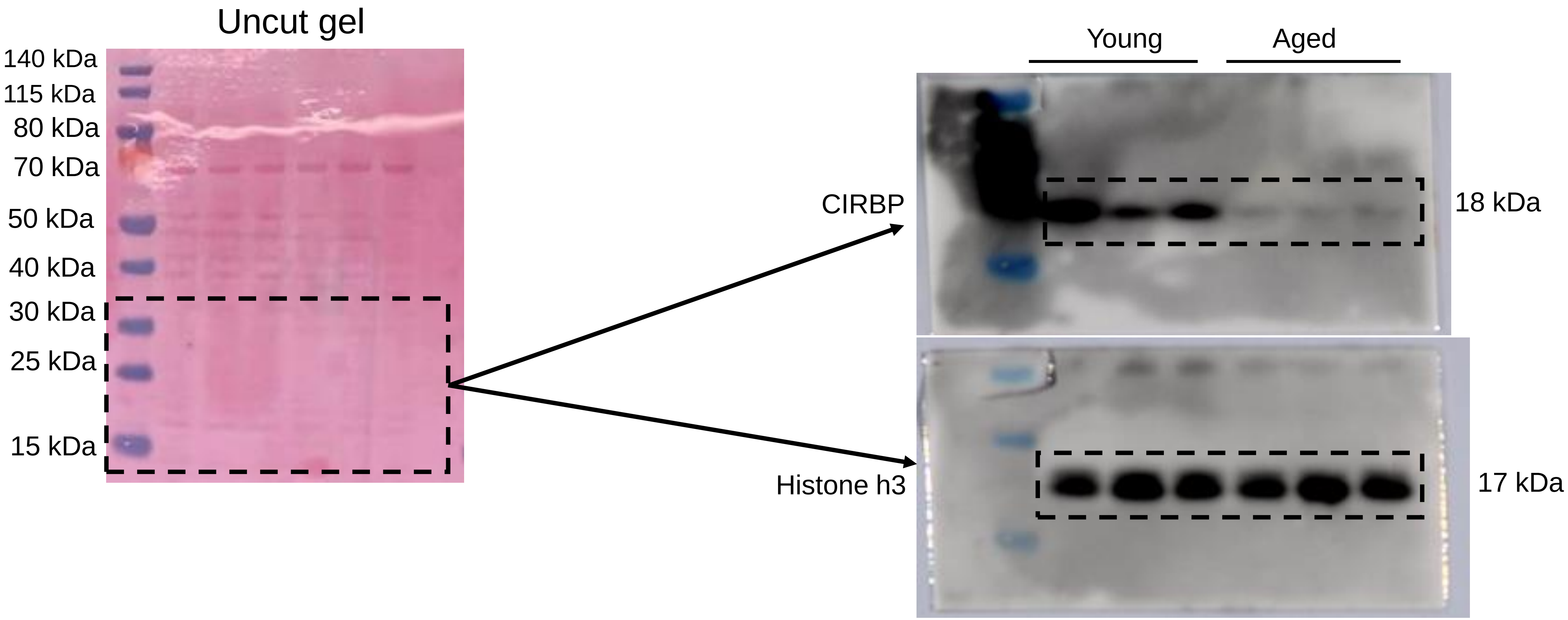
Uncut gel
140 kDa
115 kDa
80 kDa
70 kDa
50 kDa
40 kDa
30 kDa
25 kDa
15 kDa
Young
Aged
CIRBP
18 kDa
Histone h3
17 kDa

Figure S8D

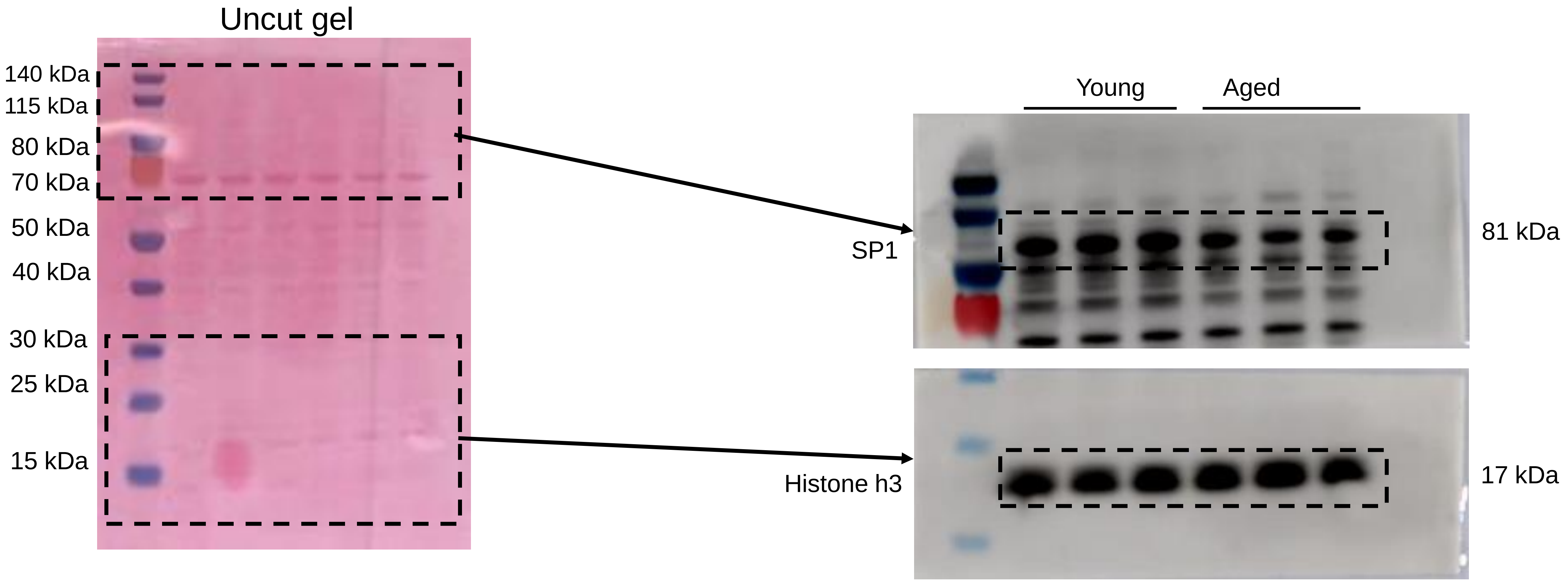
Uncut gel
140 kDa
115 kDa
80 kDa
70 kDa
50 kDa
40 kDa
30 kDa
25 kDa
15 kDa
Young
Aged
SP1
81 kDa
Histone h3
17 kDa

Figure S9B

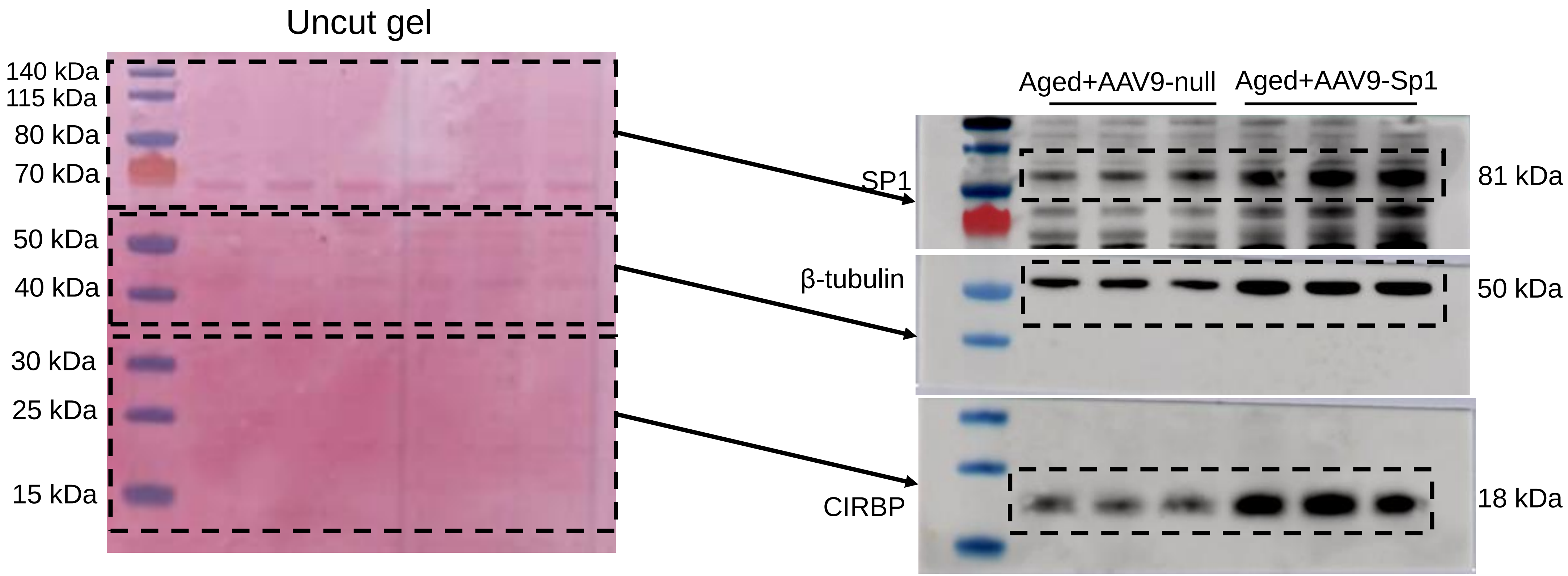

Uncut gel
140 kDa
115 kDa
80 kDa
70 kDa
50 kDa
40 kDa
30 kDa
25 kDa
15 kDa
Aged+AAV9-null
Aged+AAV9-Sp1
SP1
81 kDa
β-tubulin
50 kDa
CIRBP
18 kDa

Figure S9K

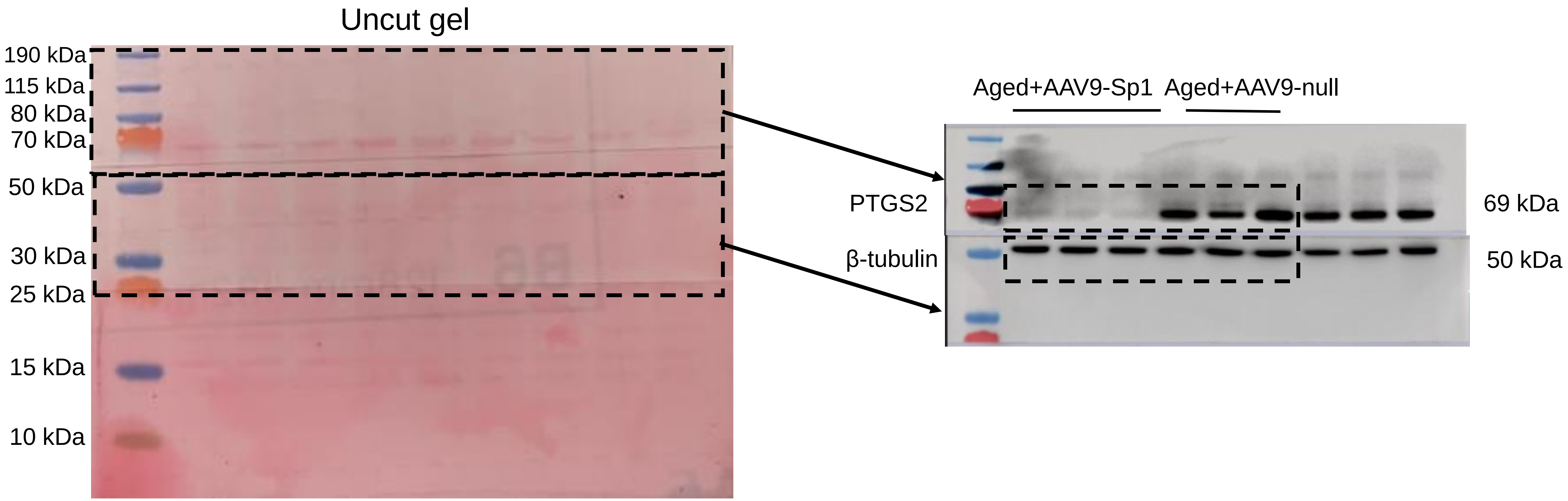
Uncut gel
190 kDa
115 kDa
80 kDa
70 kDa
50 kDa
30 kDa
25 kDa
15 kDa
10 kDa
Aged+AAV9-Sp1
Aged+AAV9-null
PTGS2
β-tubulin
69 kDa
50 kDa

Figure S10

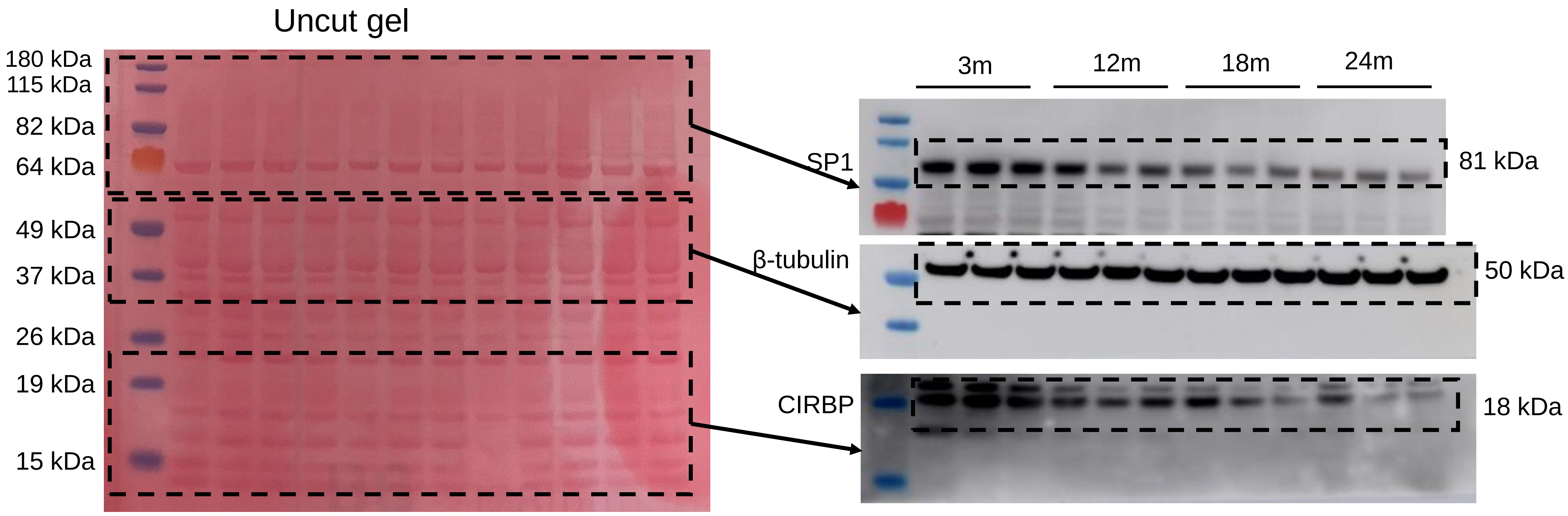
Uncut gel
180 kDa
115 kDa
82 kDa
64 kDa
49 kDa
37 kDa
26 kDa
19 kDa
15 kDa
3m
12m
18m
24m
SP1
81 kDa
β-tubulin
50 kDa
CIRBP
18 kDa

Figure S13B

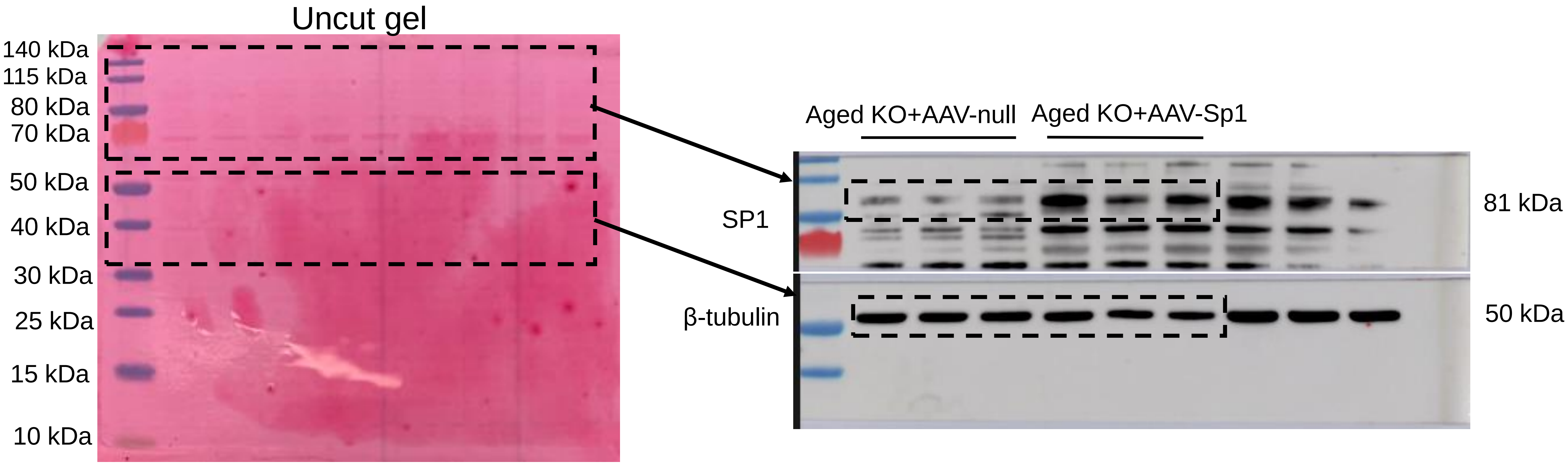

Uncut gel
140 kDa
115 kDa
80 kDa
70 kDa
50 kDa
40 kDa
30 kDa
25 kDa
15 kDa
10 kDa
Aged KO+AAV-null
Aged KO+AAV-Sp1
SP1
β-tubulin
81 kDa
50 kDa

Figure S13K

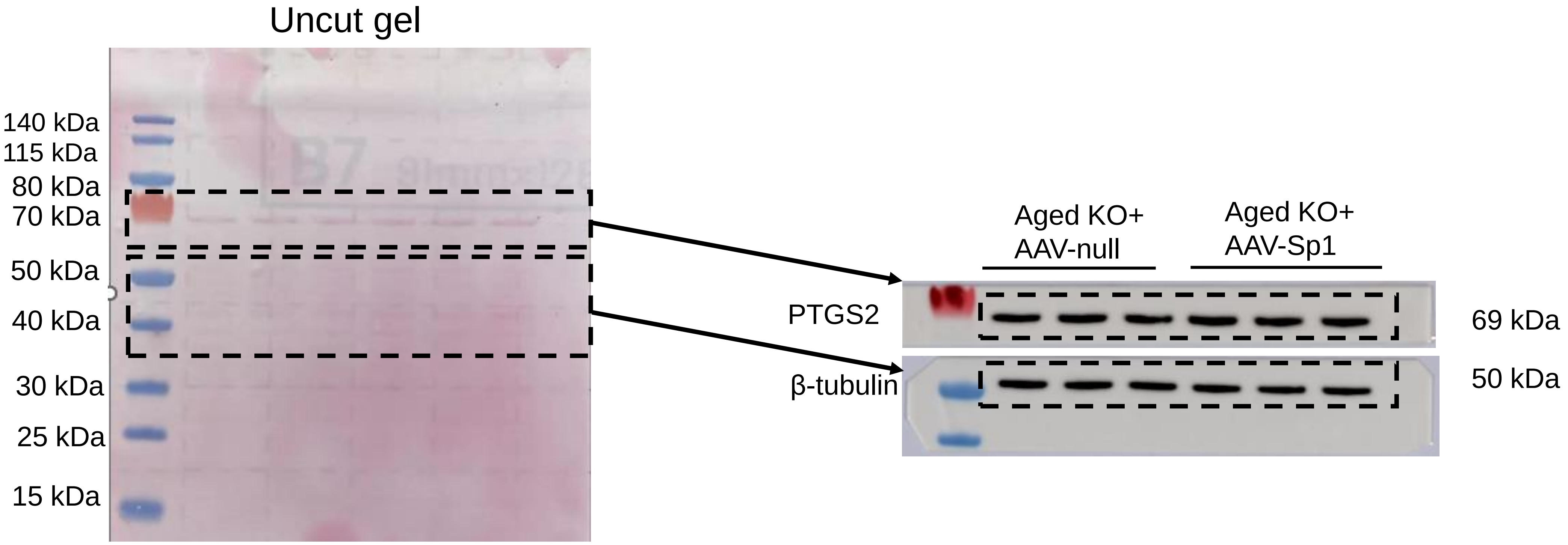
Uncut gel
140 kDa
115 kDa
80 kDa
70 kDa
50 kDa
40 kDa
30 kDa
25 kDa
15 kDa
Aged KO+
AAV-null
Aged KO+
AAV-Sp1
PTGS2
β-tubulin
69 kDa
50 kDa

Figure S14E

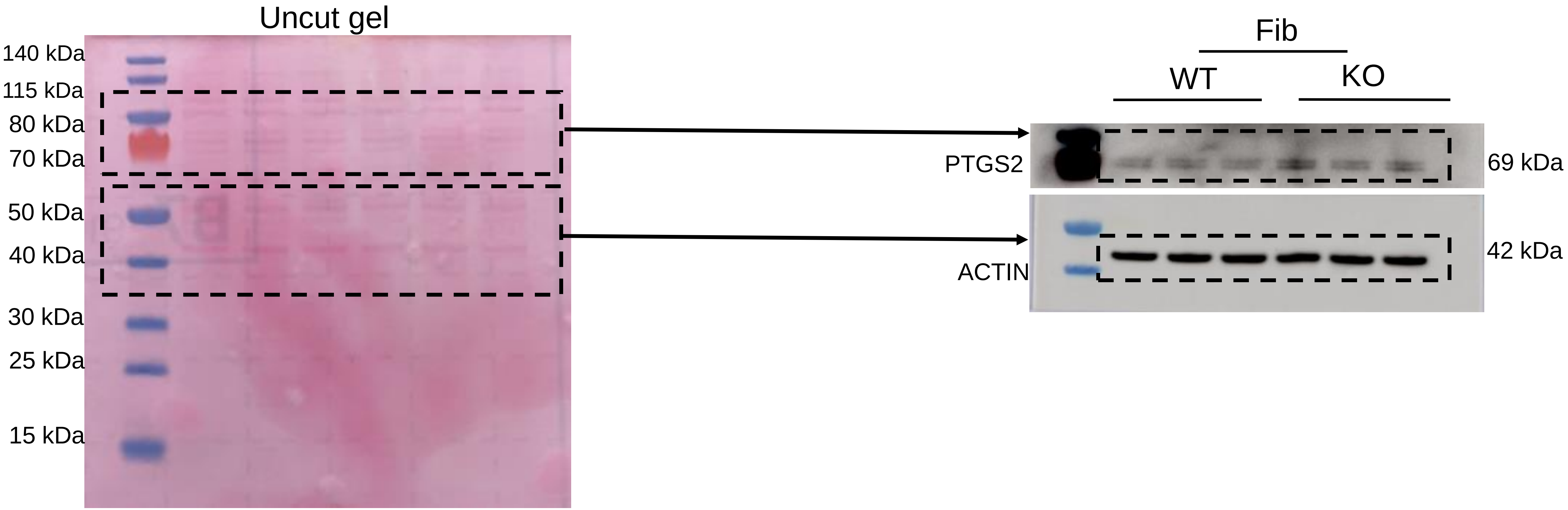
Uncut gel
140 kDa
115 kDa
80 kDa
70 kDa
50 kDa
40 kDa
30 kDa
25 kDa
15 kDa
Fib
WT
KO
PTGS2
69 kDa
ACTIN
42 kDa

Figure S14E

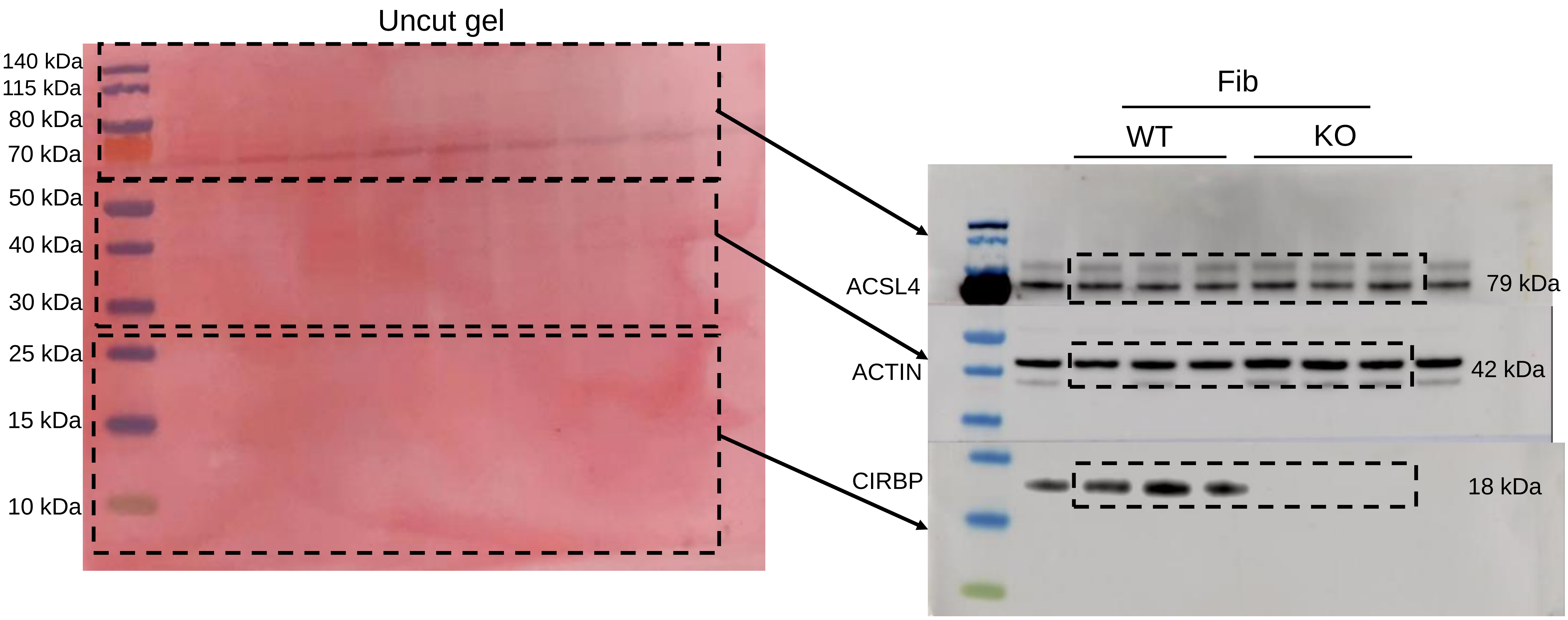

Uncut gel
140 kDa
115 kDa
80 kDa
70 kDa
50 kDa
40 kDa
30 kDa
25 kDa
15 kDa
10 kDa
Fib
WT
KO
ACSL4
ACTIN
CIRBP
79 kDa
42 kDa
18 kDa

Figure S17B

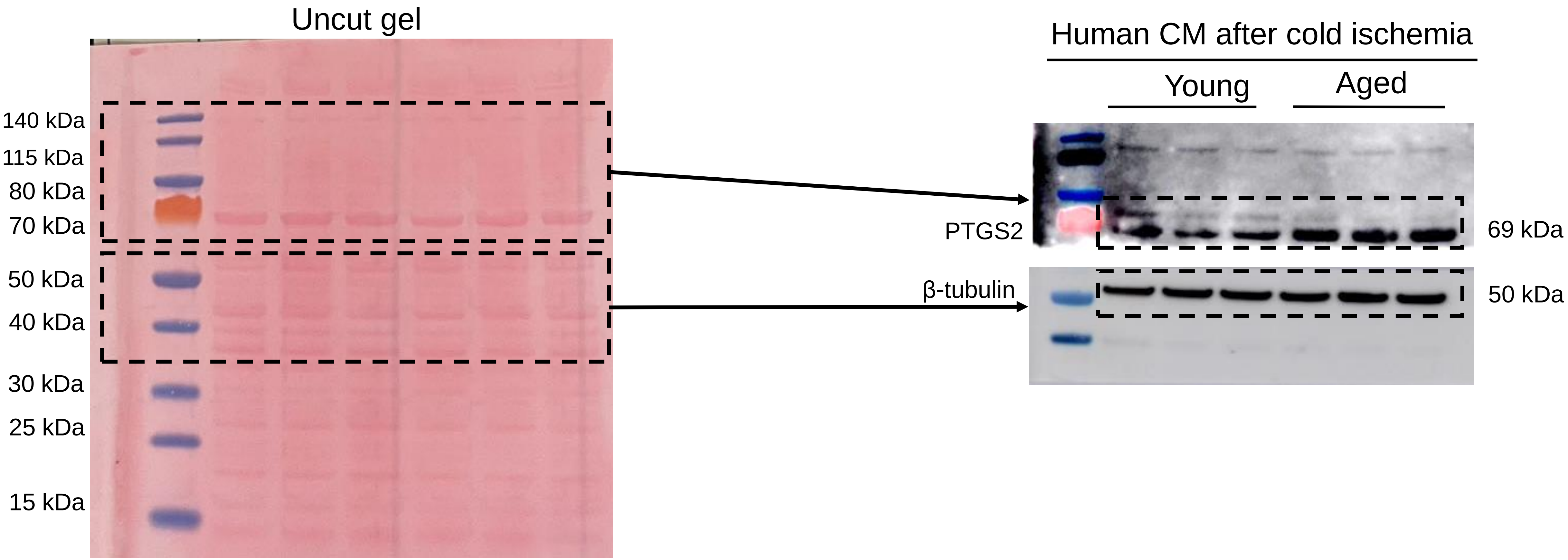

Uncut gel
140 kDa
115 kDa
80 kDa
70 kDa
50 kDa
40 kDa
30 kDa
25 kDa
15 kDa
Human CM after cold ischemia
Young
Aged
PTGS2
69 kDa
β-tubulin
50 kDa

Figure S17C

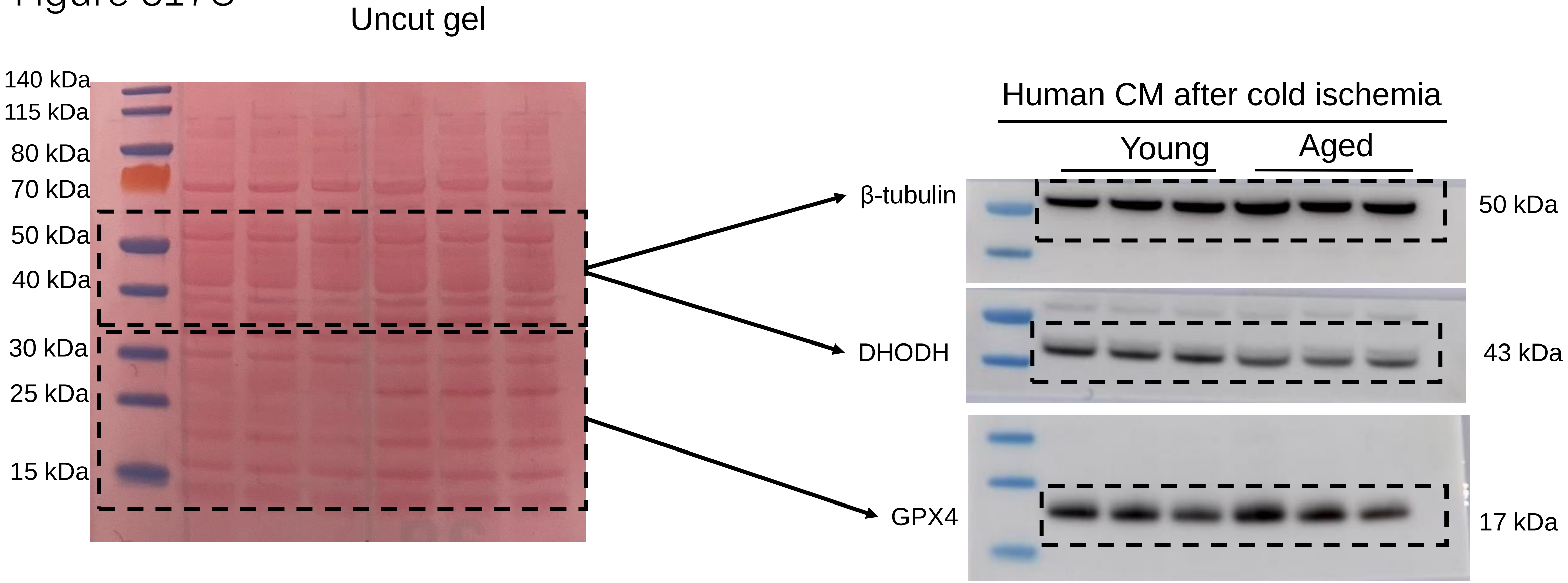
Uncut gel
140 kDa
115 kDa
80 kDa
70 kDa
50 kDa
40 kDa
30 kDa
25 kDa
15 kDa
Human CM after cold ischemia
Young
Aged
β-tubulin
DHODH
GPX4
50 kDa
43 kDa
17 kDa

Figure S18A

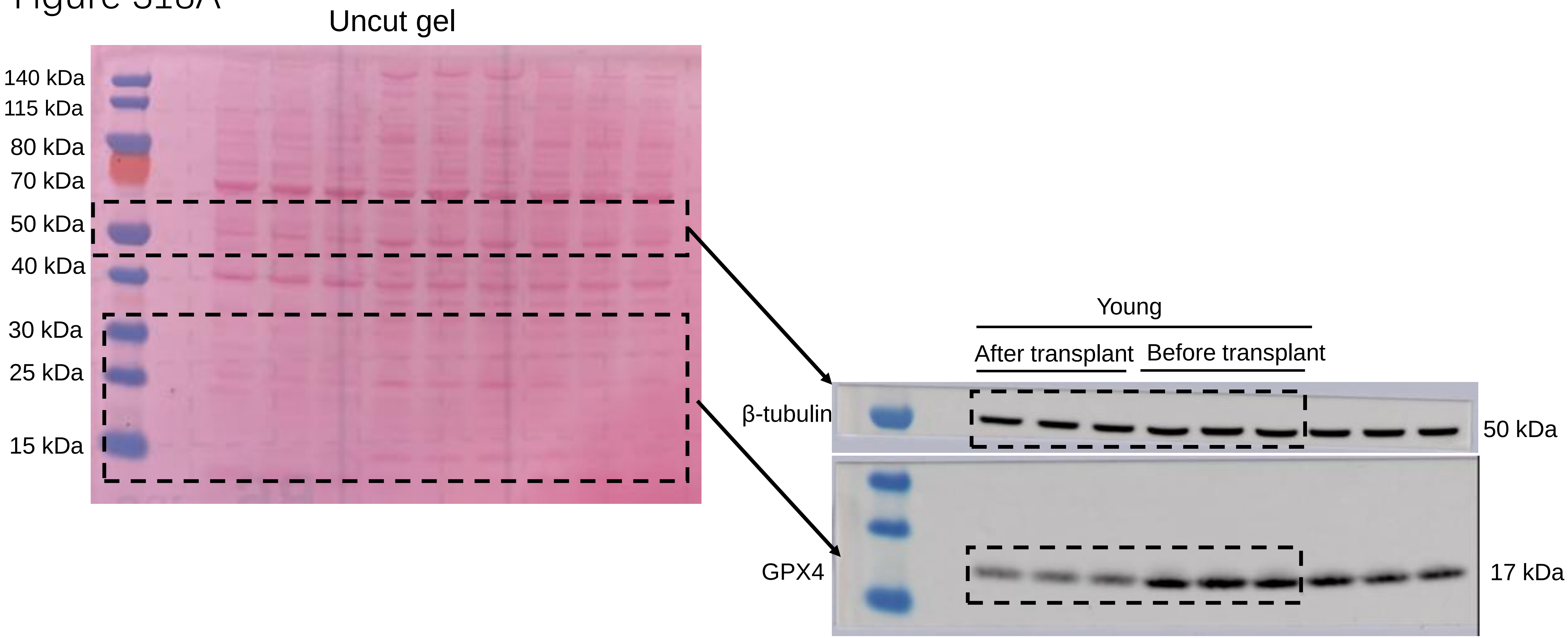
Uncut gel
140 kDa
115 kDa
80 kDa
70 kDa
50 kDa
40 kDa
30 kDa
25 kDa
15 kDa
Young
After transplant
Before transplant
β-tubulin
50 kDa
GPX4
17 kDa

Figure S18B

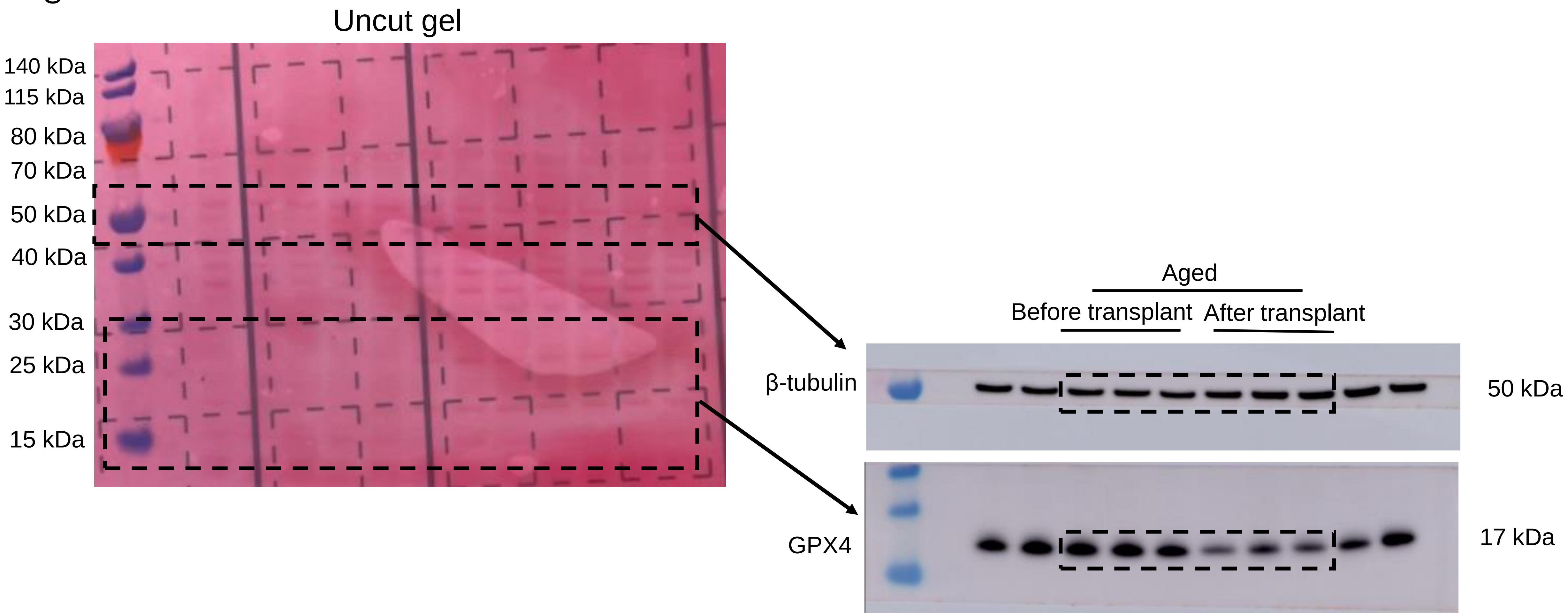
Uncut gel
140 kDa
115 kDa
80 kDa
70 kDa
50 kDa
40 kDa
30 kDa
25 kDa
15 kDa
Aged
Before transplant
After transplant
β-tubulin
50 kDa
GPX4
17 kDa

Figure S18C

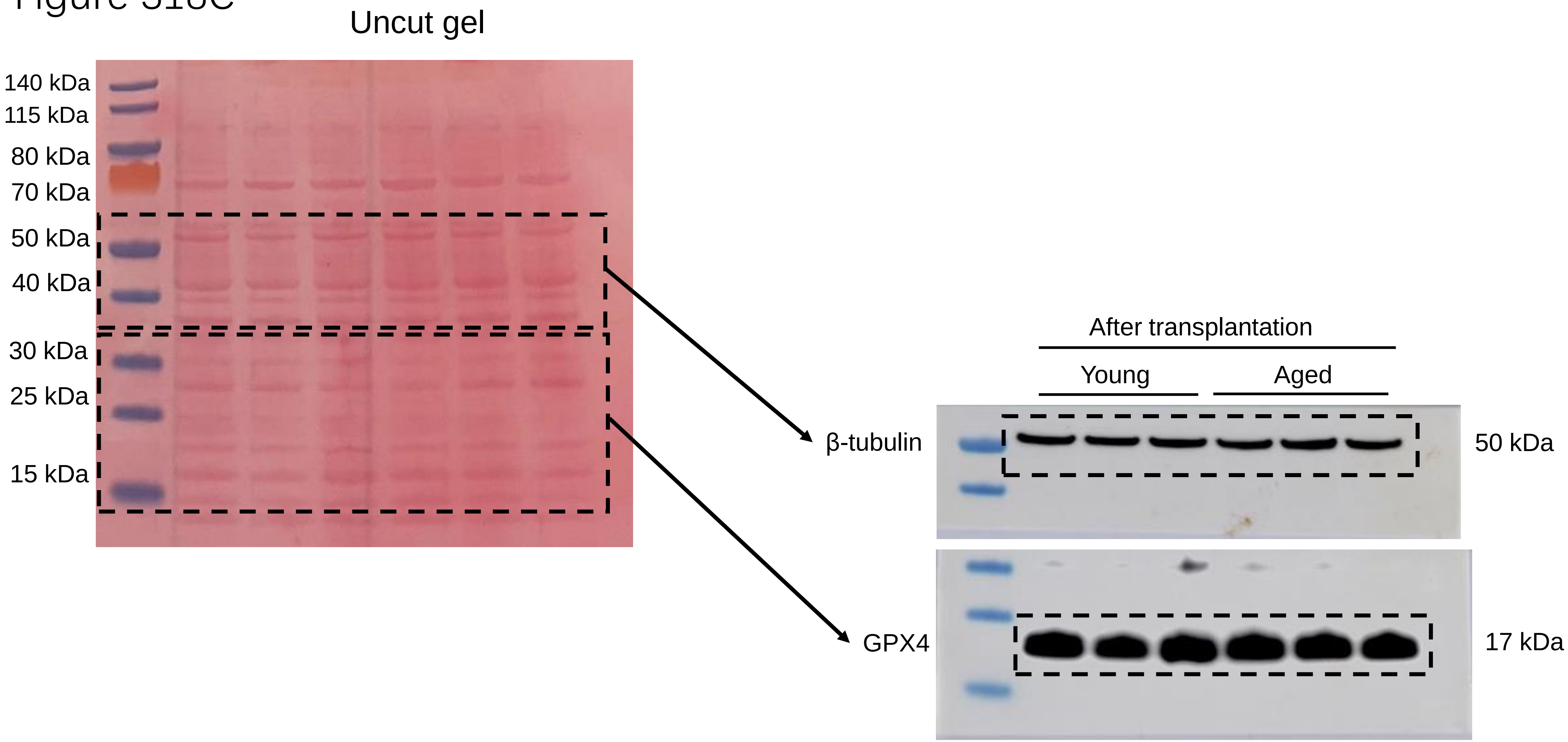
Uncut gel
140 kDa
115 kDa
80 kDa
70 kDa
50 kDa
40 kDa
30 kDa
25 kDa
15 kDa
After transplantation
Young
Aged
β-tubulin
50 kDa
GPX4
17 kDa

Figure S18D

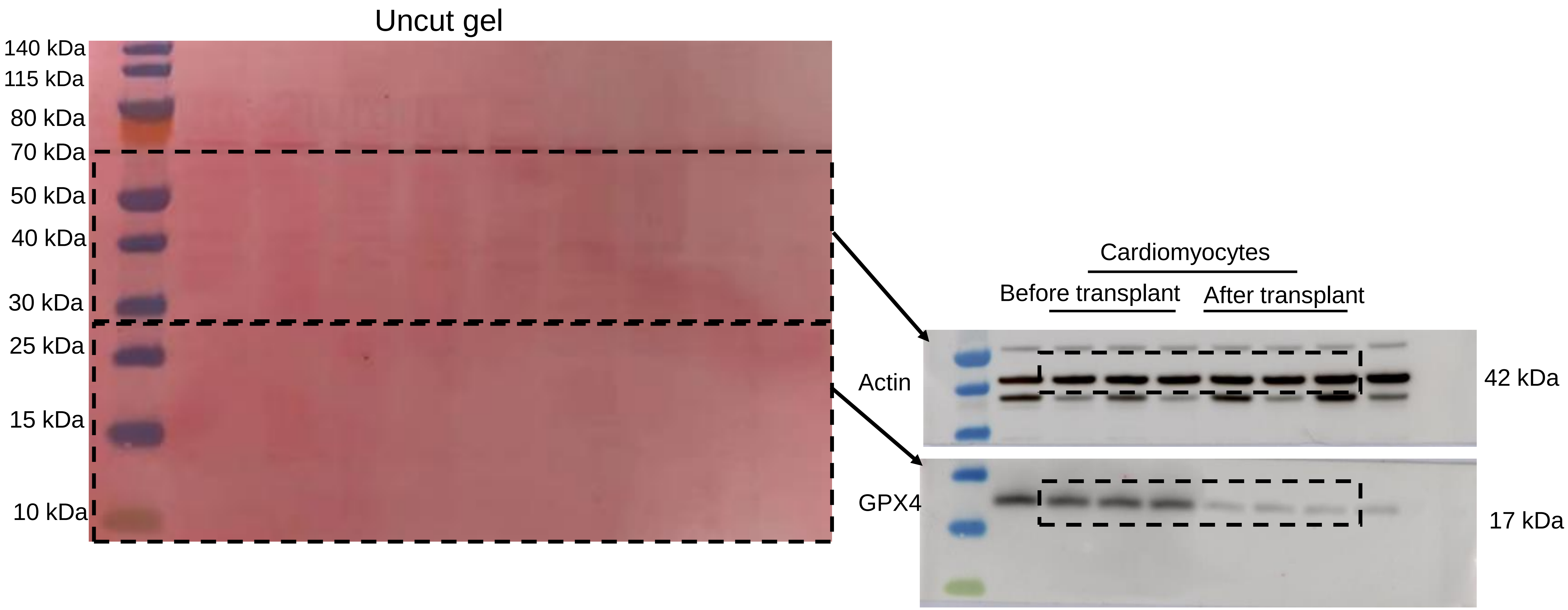

Uncut gel
140 kDa
115 kDa
80 kDa
70 kDa
50 kDa
40 kDa
30 kDa
25 kDa
15 kDa
10 kDa
Cardiomyocytes
Before transplant
After transplant
Actin
GPX4
42 kDa
17 kDa

Figure S19C

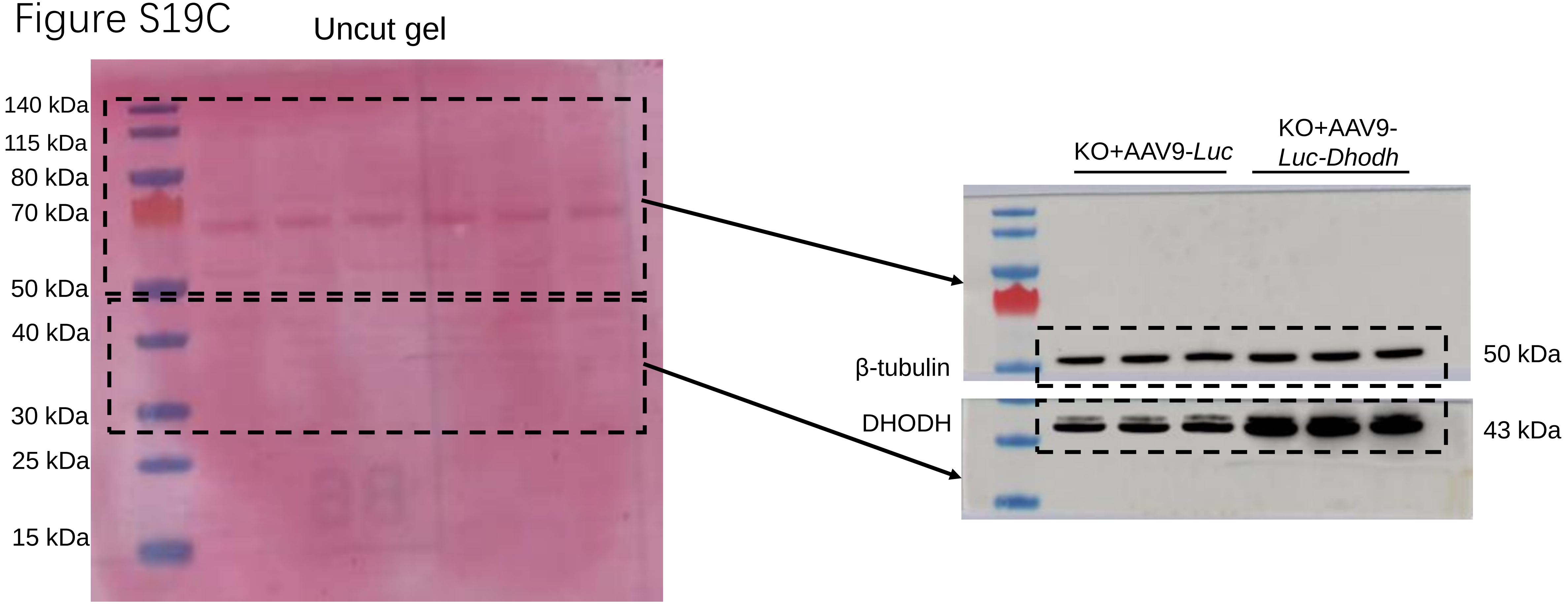
Uncut gel
140 kDa
115 kDa
80 kDa
70 kDa
50 kDa
40 kDa
30 kDa
25 kDa
15 kDa
KO+AAV9-Luc
KO+AAV9-Luc-Dhodh
β-tubulin
DHODH
50 kDa
43 kDa

Figure S19L

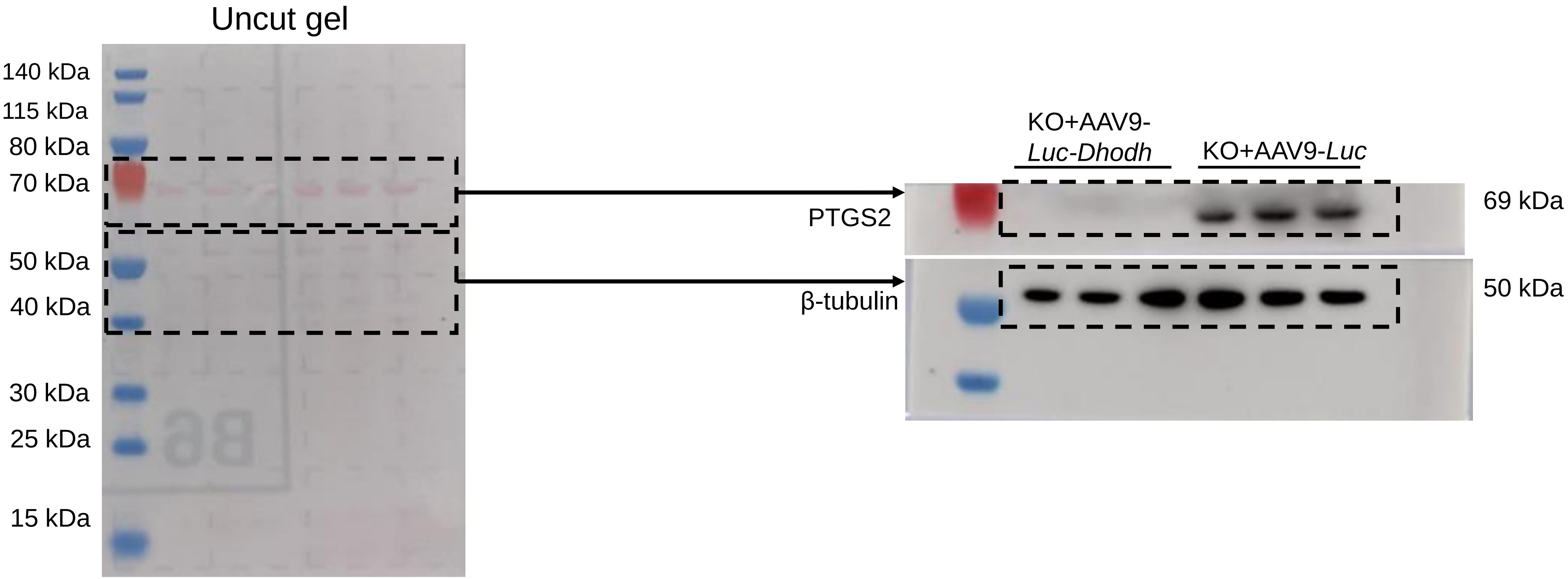
Uncut gel
140 kDa
115 kDa
80 kDa
70 kDa
50 kDa
40 kDa
30 kDa
25 kDa
15 kDa
KO+AAV9-Luc-Dhodh
KO+AAV9-Luc
PTGS2
β-tubulin
69 kDa
50 kDa

Figure S20A

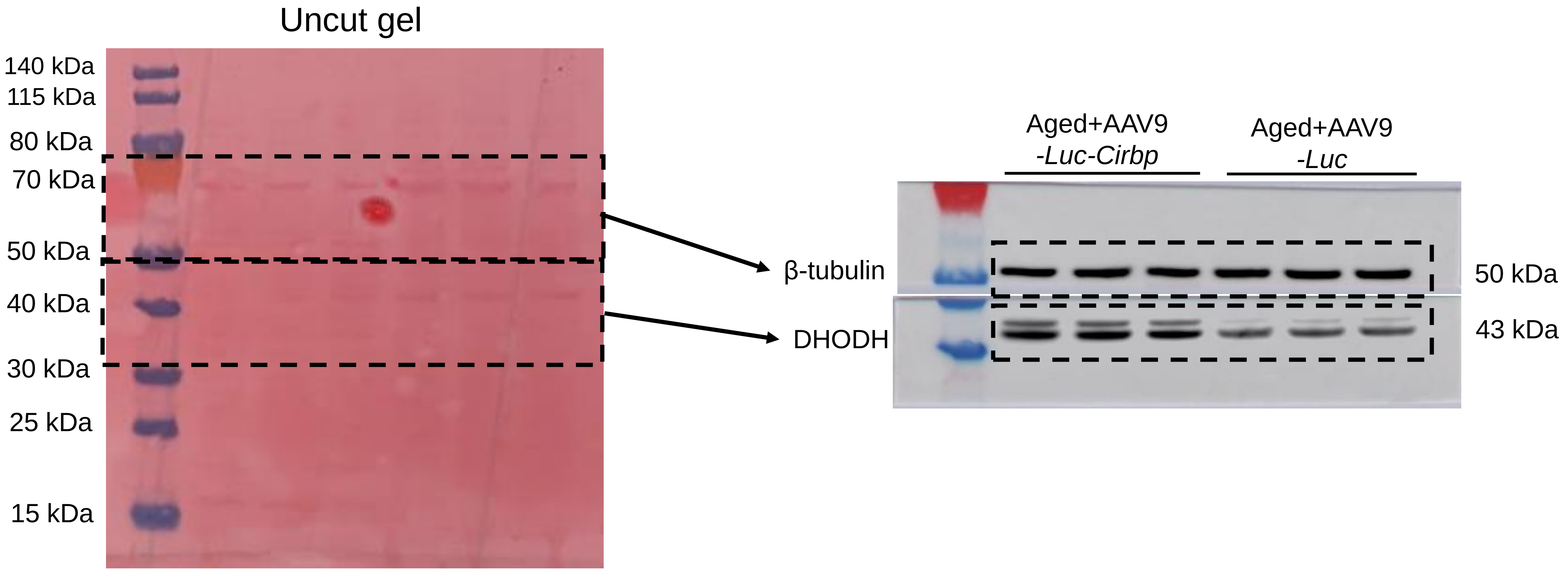
Uncut gel
140 kDa
115 kDa
80 kDa
70 kDa
50 kDa
40 kDa
30 kDa
25 kDa
15 kDa
β-tubulin
DHODH
Aged+AAV9 -Luc-Cirbp
Aged+AAV9 -Luc
50 kDa
43 kDa
